# Misregulation of T-box transcription factors drives BNP–NPR1 signalling underlying chronic itch

**DOI:** 10.1038/s41467-026-75726-x

**Published:** 2026-07-17

**Authors:** Xiaolong Dai, Michael Kitching, Wenke Cheng, Rengang Luo, Lianlian Li, Renkai Zhu, Chunxu Shan, Weiwei Chen, Timo Buhl, Andrea Szegedi, Jorg Buddenkotte, Shouming Xu, Jiafu Wang, Jianghui Meng

**Affiliations:** 1 School of Life Sciences, Henan University, Kaifeng, Henan China; 2 School of Biotechnology, Faculty of Science and Health, Dublin City University, Glasnevin, Dublin, Ireland; 3 Immuno Biotech Co. Ltd, Zhengzhou, Henan China; 4 Department of Dermatology, Venereology and Allergology, University Medical Center Göttingen, Göttingen, Germany; 5 Department of Dermatology, Centre of Excellence, Faculty of Medicine, University of Debrecen, Debrecen, Hungary; 6 HUN-REN-DE Allergology Research Group, Debrecen, Hungary; 7 Department of Dermatology and Venereology, Hamad Medical Corporation, Doha, Qatar; 8 Translational Research Institute, Academic Health System, Hamad Medical Corporation, Doha, Qatar; 9 Dermatology Institute, Academic Health System, Hamad Medical Corporation, Doha, Qatar

**Keywords:** Pruritus, Skin diseases, Transcriptional regulatory elements

## Abstract

Chronic itch poses a significant clinical burden, with activation of the brain natriuretic peptide (BNP) pathway as a hallmark, though its transcriptional control remains unclear. We identify T-box transcription factor 20 (TBX20) as upregulated in skin of atopic dermatitis patients, with increased expression in NPR1^+^ keratinocytes. ChIP peak calling, ChIP-qPCR and dual-luciferase assays show TBX20 activates the NPR1 promoter. In mice, keratinocyte-specific TBX20 knockout (TBX20^fl/fl^; K14-Cre) reduces scratching, IL-4 and Oncostatin M levels, basophil markers, and decreases cutaneous NPR1 expression. TBX20 is also expressed in a subset of NPR1^+^ sensory neurons, and neuron-specific deletion similarly alleviates itch, basophil infiltration, and barrier dysfunction. RNA-seq identifies lipocalin-2 as a downstream effector of the TBX20-NPR1 axis, and its deletion reduces itch. Conversely, AAV-mediated TBX20 rescue exacerbates symptoms. Neuregulin 1 suppresses TBX20, attenuates IL-13-driven NPR1 signalling, and reduces itch. These findings define a TBX20-NPR1 axis as a critical regulator of chronic itch and skin dysfunction.

## Abstract (web summary)

Here, the authors show that TBX20 helps drive long-lasting itch by boosting BNP signalling in skin and nerves. In mice, blocking this pathway reduced itching, inflammation, and skin damage, suggesting a potential treatment target for chronic itch.

## Introduction

Chronic itch is a persistent, debilitating condition associated with various dermatological and systemic diseases^1–4^. Current treatments are limited and often fail to address the root cause due to an insufficient understanding of its pathogenesis. Brain Natriuretic Peptide (BNP), a 32 aa cyclic peptide encoded by the *Nppb* gene, primarily binds to natriuretic peptide receptor 1 (NPR1) and receptor 3 (NPR3), and to a lesser extent NPR2^5,6^. BNP is expressed in skin, a subset of sensory neurons, and is known to activate spinal cord neurons involved in both histaminergic and non-histaminergic itch^5–9^. Intrathecal (*i.t*.) injection of BNP induces robust itch in mice, while *Nppb* KO mitigates scratching responses to multiple pruritogens^7^. To date, the pruritogenic effect of BNP has been demonstrated in mouse models, in intravenous^10^, systemic^10^ and intradermal (*i.d*.) injections^11^.

BNP levels correlate with the severity of chronic itch, but not pain conditions^12^, and are responsive to pruritogens^5^. Enhanced *Nppb* mRNA is observed in the sensory ganglia of PAR2 overexpressing (OE) mice treated with house dust mite (HDM) in a manner dependent on disease severity^13^. IL-31 increases BNP transcription and secretion from sensory neurons, which orchestrates the release of itch-related cytokines in the skin^13^. Similarly, sensory neuronal BNP synthesis is upregulated by the pruritogen ET-1^14^. Elevated *Nppb* levels are noted in the skin of IL-31 OE mice^13^, in human lesional atopic dermatitis (AD)^13^, psoriasis^15^, and prurigo nodularis^5^, indicating a disease-regulated upregulation mechanism. In addition to BNP, its receptor NPR1 is also upregulated in lesional AD skin^13,16^. BNP activation of keratinocyte-NPR1 leads to upregulation of TRPV3 (Transient receptor potential vanilloid-3), increasing Serpin E1 release, which directly triggers sensory neurons to generate an itch response in mouse cheek models^8^. Therapeutic targeting of NPR1 via antagonists has shown efficacy in murine models of chronic pruritus^9,17^. Knockout of *Npr1* or silencing its expression impairs itch responses to *i.d*. administration of the non-histaminergic pruritogen chloroquine^6^. Conversely, increased *Npr1* expression in the spinal cord in models of allergic contact dermatitis (ACD) suggests a role in central sensitisation^18,19^. Thus, understanding the upregulation of BNP-NPR1 signalling and its role in itch amplification is critical for controlling this disease.

T-box transcription factor TBX20 is a key regulatory protein in development, particularly critical in heart development^20,21^. It regulates the proliferation and differentiation of cardiomyocytes, the formation of the atria, and the development of vascular endothelium during embryonic development^22^. The non-cardiac function of TBX20 is unclear, but has been related to neural function, embryonic tissue development, cancer progression, and potentially immune regulation^23–26^. Although TBX20 expression has been reported in sensory neurons^27^, motor neurons^26^ and in the skin^16^, and is related to regulation of channel proteins and receptors^21,28,29^, its function in sensory transduction and skin pathology remains unexplored. TBX20 conserved binding site^21^ along with the TBX20 cooperative factor, GATA4^30^, has been identified in the NPR1 gene. Our ChIP-peak analysis revealed potential TBX20 binding sites in the upstream regulatory region of the NPR1 gene. These findings provide a theoretical basis for further research into the functional relationship between TBX20 and its regulation of BNP-NPR1 signalling in chronic itch. We hypothesise that TBX20 can activate the BNP/NPR1 signalling pathway by enhancing NPR1 expression, leading to excessive secretion of cutaneous inflammation and itch-related factors in the skin. This activation may cause abnormal changes in skin-neural signalling pathways, ultimately exacerbating the “itch-scratch” vicious cycle.

In this study, we investigated the role of TBX20 in keratinocytes and sensory neurons by selectively knocking out the gene in models of AD and ACD. Complementary gain- and loss-of-function approaches revealed that TBX20 is a critical regulator of itch, immune cell infiltration, and epidermal barrier integrity, identifying it as a promising therapeutic target in chronic pruritic conditions.

## Results

### Epidermal TBX20 protein levels are elevated in human AD skin, with increased transcripts observed in itch conditions

**Fig. 1:**
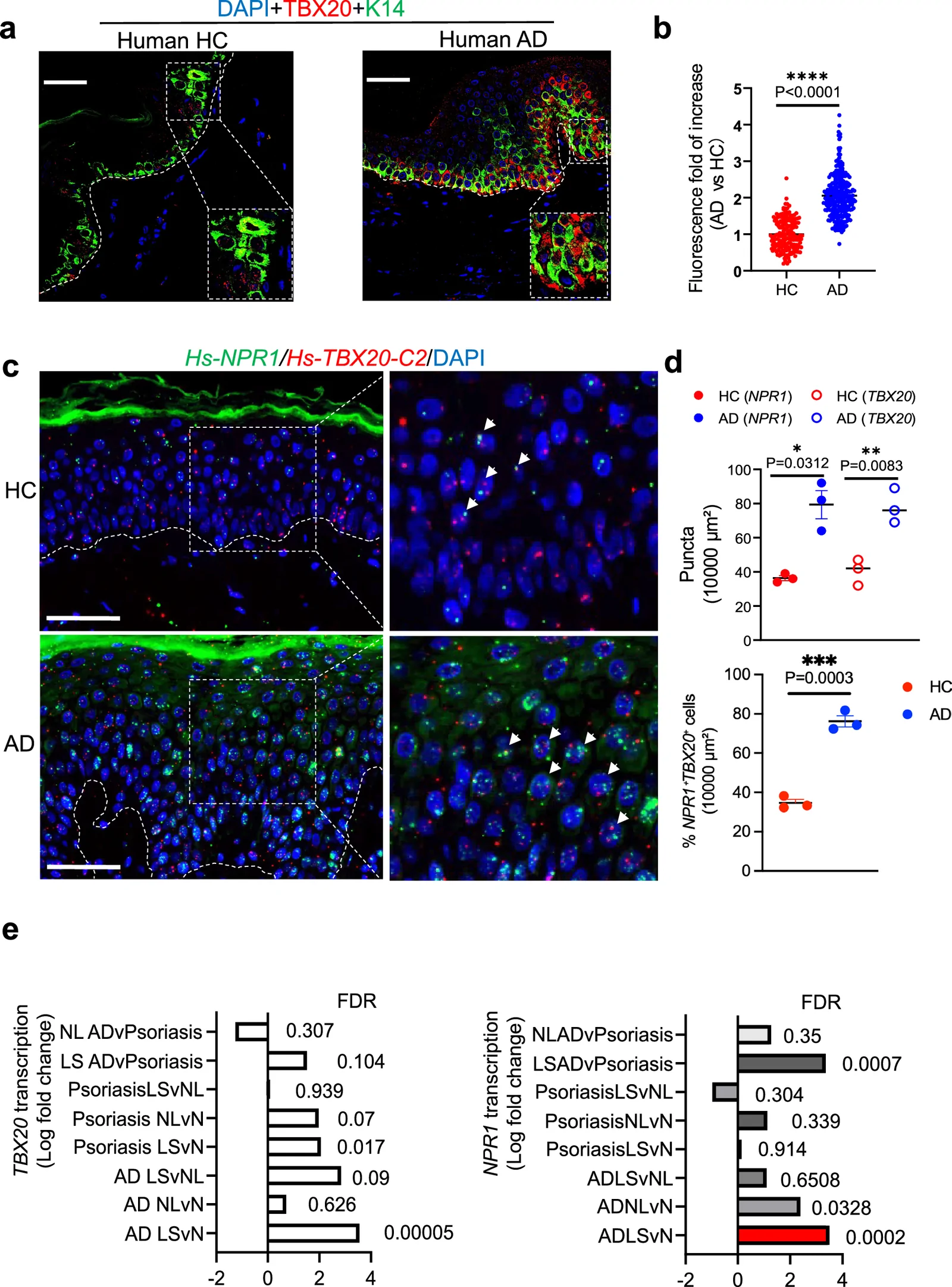
Immunohistochemical analysis of TBX20,RNAscope and transcriptomic profiling of *TBX20* and *NPR1* in skin from healthy individuals and patients with chronic itch. Confocal immunohistochemistry images (**a**) and fluorescence intensity analysis (**b**) of skin samples from healthy control (HC) and patients with lesional AD. Fluorescence intensity was quantified in randomly selected K14^+^ cells. Scale bar = 50 μm. Representative RNAscope images (**c**) and quantification of the numbers of puncta representing *TBX20* and *NPR1* transcripts in marker-positive cells (top), as well as frequency of keratinocytes co-expressing *TBX20* and *NPR1* transcripts (bottom), in LAD epidermis compared with HC (**d**). Scale bar = 50 μm. Arrowheads indicate cells containing both red and green puncta. **e** Relative transcription levels of *TBX20* and *NPR1* in the RNA-seq datasets from skin-stripped samples of patients with lesional (LS), non-lesional (NL) AD or psoriasis, and normal (N) skin. The dataset used is reanalysed from reference ^16^. Data are presented as Log2fold change, with the FDR (false discovery rate) indicated. Data **b** and **d** presented as Mean ± SEM; three donors per condition were analysed. A two-tailed, unpaired Student’s *t*-test was used for analyses in (**b**) and (**d**), with Welch’s correction applied in (**d**). ^*^P < 0.05, ^**^P < 0.01, ^***^P < 0.001, ^****^P < 0.0001. Source data (**b**, **d** and **e**) are provided as a Source Data file.

To investigate whether TBX20 expression changes in pruritic lesions, we assessed both protein and mRNA levels in human lesional AD and healthy skin samples. Confocal imaging revealed markedly elevated TBX20 protein expression in the basal K14^+^-keratinocytes of lesional AD (LAD) skin compared with healthy controls (HC), with partial nuclear localization (Fig. 1a). Quantification showed a 2-fold increase in TBX20 signal in randomly selected K14^+^ keratinocytes (Fig. 1b). RNAscope detected *TBX20* and *NPR1* transcripts predominantly in the epidermis of both LAD and HC samples (Fig. 1c). Assay quality was validated using positive and negative control probes (Supplementary Fig. 1). Quantitative analysis revealed that the numbers of puncta for both *TBX20* and *NPR1* transcripts were elevated in LAD epidermis compared with HC (Fig. 1d, **top**). Additionally, keratinocytes co-expressing *TBX20* and *NPR1* transcripts were also increased (Fig. 1d, **bottom**), suggesting coordinated epidermal upregulation of these genes in LAD.

Our previous report demonstrated that NPR1 protein level is also elevated in human keratinocytes in the skin of AD patients^13^. To determine whether changes in *NPR1* transcription parallel those of *TBX20* in itch conditions, we reanalysed published RNA-seq datasets from skin-stripped samples of AD and psoriasis patients, comparing lesional (LS) and non-lesional (NL) skin to normal (N) skin^16^. This analysis revealed a similar upregulation of *TBX20* and *NPR1* transcription levels in AD lesional skin compared to normal skin (N) (Fig. 1e). Based on this, we hypothesised that TBX20 and NPR1 are functionally connected or co-regulated in the context of itch skin.

### ChIP peak calling identified TBX20 protein–NPR1 DNA interaction, which was confirmed by ChIP-qPCR and dual-luciferase reporter analysis in keratinocytes in response to BNP treatment

To computationally predict putative TBX20 binding sites regulating NPR1 expression in human keratinocytes, we interrogated epigenetic datasets generated by the ENCODE project. All analyses were performed in RStudio (v 4.4.1). ChIP-seq datasets were from ENCODE and included H3K4me1 (foreskin keratinocyte, ENCSR780KNM), H3K4me2 (foreskin keratinocyte, ENCSR926BCX), H3K4me3 (keratinocyte male, ENCSR113UDS), and H3K27ac (female keratinocyte, ENCSR000ALK).

We prioritized analysis of an H3K27ac-marked regulatory region proximal to the *NPR1* locus based on its potential regulatory relevance. This region showed enrichment for enhancer- and promoter-associated histone modifications (H3K4me1, H3K4me2, and H3K4me3) and exhibited moderate evolutionary conservation based on Phylop and Phast scores. These, along with PhyloP and PhastCons conservation data, were analysed and visualised using the GenomicScores and Gviz packages, respectively^31,32^. The reference genome assembly used was Ensembl Hg19, spanning chromosome 1: 153,621,113 to 153,666,468.

**Fig. 2:**
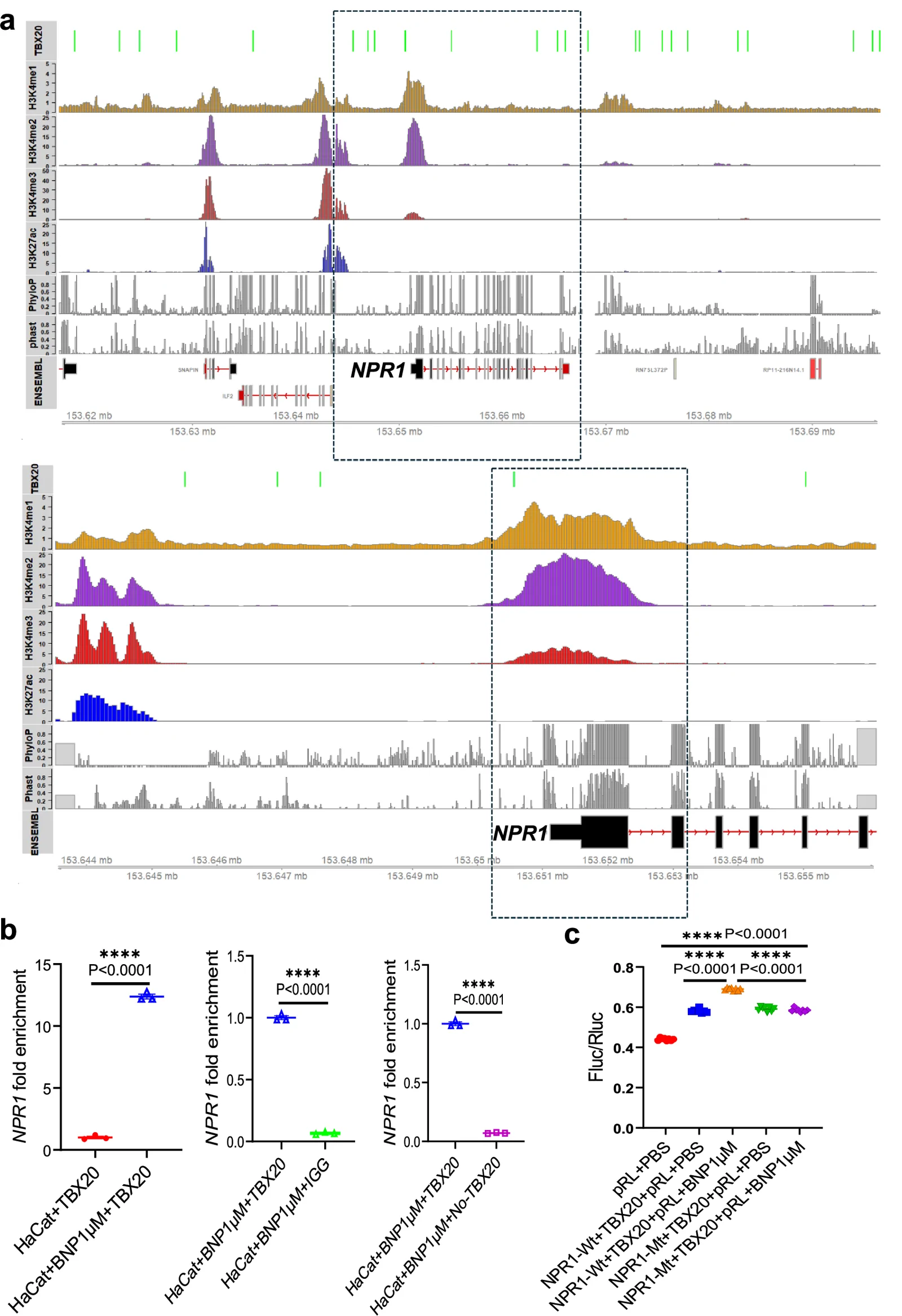
NPR1 gene contains multiple conserved TBX20 DNA-binding motifs in its upstream and promoter regions. The interaction of the TBX20 protein with the NPR1 gene is enhanced in response to BNP stimulation in human keratinocytes. **a** ChIP peak calling analysis revealed that the human NPR1 gene contains multiple conserved TBX20 DNA-binding motifs. ChIP-seq data from ENCODE, along with PhylopP and PhastCons data, were analysed and visualised using the GenomicScores and Gviz packages, respectively. The datasets are from references^31,32^. **b** ChIP-qPCR demonstrated the interaction of TBX20 protein with the NPR1 gene in response to BNP stimulation in the HaCaT cells. TBX20: anti-TBX20 antibody; IGG: non-immune IgG controls; no-TBX20: no anti-TBX20 antibody and no IgG controls. Data are presented as Mean ± SEM. *N* = 3 independent cultures were analysed. **c** Fluc/Rluc reporter activity was measured in BNP-induced HaCaT cells transfected with NPR1-Wt or NPR1-Mt promoter construct (sequences provided in Supplementary Fig. 2), a TBX20 overexpression plasmid (pcDNA3.1-TBX20), and the Renilla luciferase plasmid pRL-TK as an internal control. Firefly luciferase (Fluc) activity was normalized to Renilla luciferase (Rluc), and the Fluc/Rluc ratio was calculated. Cells transfected with pRL-TK alone served as the baseline control. Data are presented as mean ± SEM; *n* = 8 independent repeats. A two-tailed unpaired Student’s t-test (**b**) and two-way ANOVA followed by Tukey’s multiple comparisons test (**c**) were used. ****P<0.0001. Source data (**a**–**c**) are provided as a Source Data file.

Within this region, we searched for the TBX20 consensus binding motif (5’ AGGTGTG 3’), in sequences enriched for enhancer-associated marks (H3K4me1 & H3K4me2), active regulatory histone modifications (H3K4me2 & H3K27Ac), and signs of evolutionary sequence conservation (PhyloP/PhastCons score [denoted PhyloP & Phast, respectively]). Our focus on the region immediately upstream of *NPR1* revealed multiple putative TBX20 binding sites (enclosed by a box in Fig. 2a). One region, in particular, exhibited modest levels of H3K4me1, H3K4me2, and H3K4me3, along with a relatively high PhyloP score (Fig. 2a**, lower panel**). In addition, the upstream and promoter regions of the human *NPR1* gene (NCBI Homo sapiens genome data version 109.20190905) contain multiple conserved TBX20 DNA-binding motifs (AGGTGTG)^21^. This integrative analysis supports our hypothesis that TBX20 acts as a transcriptional regulator of *NPR1*, thereby amplifying BNP signalling and contributing to pruritic responses.

To experimentally assess TBX20 binding to the NPR1gene, we performed a ChIP assay (EpiQuik) in the human keratinocyte cell line HaCaT. Cells were treated with BNP for 24 hours, after which the immunoprecipitated TBX20-chromatin complex was isolated. Cross-linked DNA was subsequently subjected to qPCR analysis. BNP-treated HaCaT cells showed increased enrichment of *NPR1* DNA in TBX20-immunoprecipitated chromatin compared to vehicle-treated cells or negative controls (non-immune IgG and no-antibody samples) (Fig. 2b).

To investigate whether TBX20 directly regulates *NPR1* transcription, a dual-luciferase reporter assay was performed. The human *NPR1* promoter region (−2000 to +200 bp relative to the transcription start site [TSS]; NC_000001:153676688–153678887) was cloned upstream of the firefly luciferase gene in the pGL3-Basic vector to generate the wild-type reporter construct (Supplementary Fig. 2).

Four putative TBX20-binding sites within the NPR1 promoter were predicted using JASPAR (MA0689.1; relative score > 0.8, score > 8), located at positions 1333–1343 bp (0.94; 14.7), 1433–1443 bp (0.87; 10.5), 1625–1635 bp (0.85; 9.6), and 1854–1864 bp (0.81; 7.4). To assess the functional relevance of these sites, a mutant NPR1 promoter fragment harbouring simultaneous mutations at all four predicted TBX20-binding sites was synthesized (Supplementary Fig. 2) and cloned into the same reporter vector.

HaCaT cells were co-transfected with either the wild-type or mutant NPR1 promoter construct, a TBX20 overexpression plasmid (pcDNA3.1-TBX20), and the Renilla luciferase plasmid pRL-TK as an internal control. Promoter activity was determined by calculating the ratio of firefly to Renilla luciferase activity (Fluc/Rluc).

Dual-luciferase assays showed that TBX20 significantly enhanced *NPR1* promoter activity following BNP stimulation in HaCaT cells transfected with the wild-type construct. In contrast, the synthesized mutant promoter construct abolished this activation, indicating that TBX20 regulates *NPR1* transcription through these specific binding sites (Fig. 2c). Similar results were observed in HEK293 cells (Supplementary Fig. 3a).

Together, these findings demonstrate that TBX20 interacts with the NPR1 gene in response to BNP, suggesting a mechanism for the transcriptional activation of NPR1 observed under itch and AD conditions.

To determine whether NPR1 regulates TBX20 expression, HaCaT cells were treated with the NPR1 antagonist A71915 (1 µM for 24 h; dose selected based on previous study^33^, and corresponding to the reported IC₅₀ for human NPR1^9^) or vehicle control, in the presence of BNP (1 µM). *TBX20* expression was quantified by RT–qPCR. BNP significantly increased TBX20 expression, and this effect was attenuated by A71915 (Supplementary Fig. 3b), indicating that BNP-mediated NPR1 activation regulates *TBX20* transcription in human keratinocytes.

### Keratinocyte-selective TBX20 knockout attenuates BNP-induced itch and reduces inflammatory gene expression

**Fig. 3:**
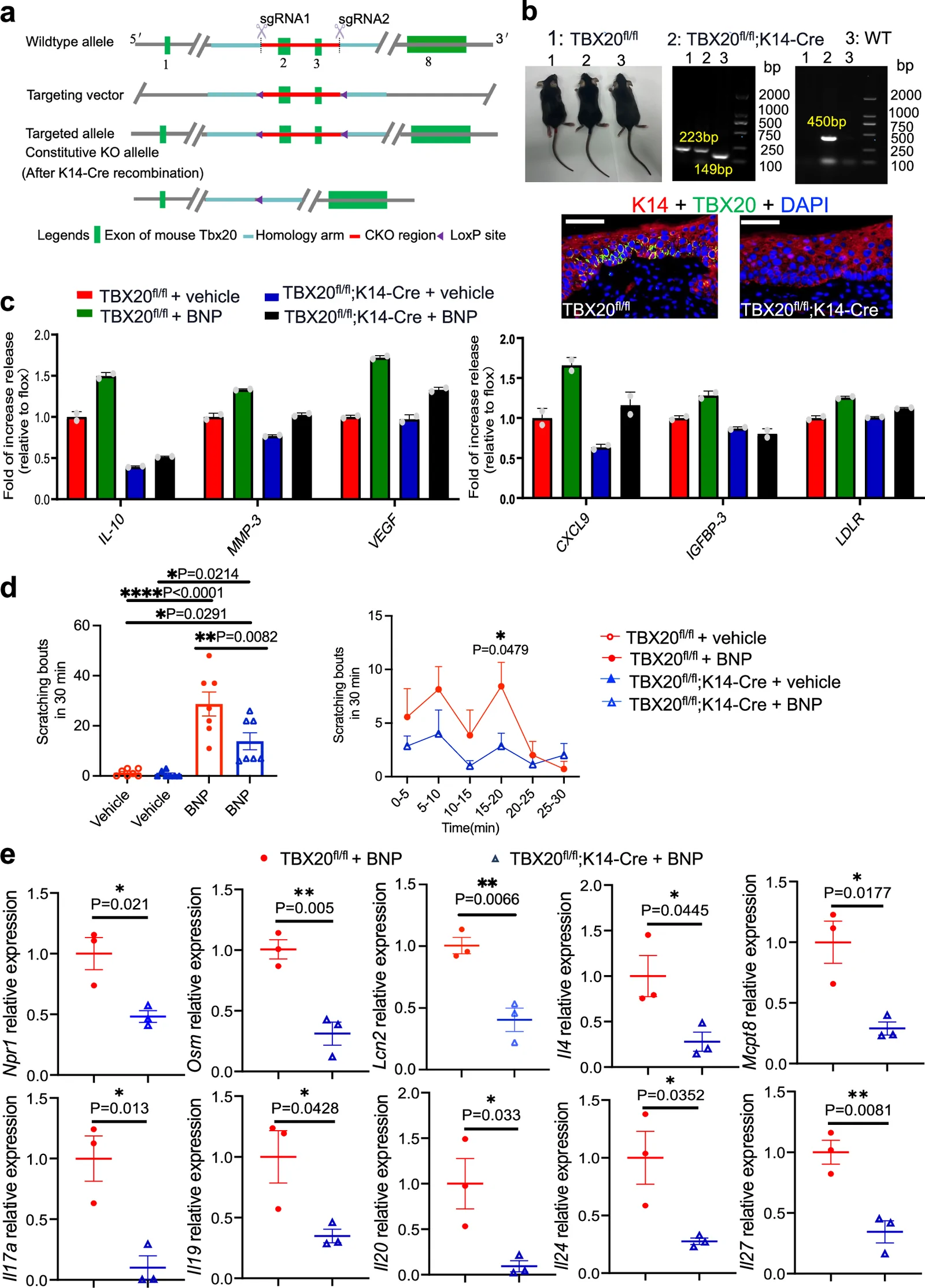
TBX20^fl/fl^;K14-Cre mice exhibited reduced cytokine release and BNP-induced cheek itch, associated with downregulation of *Npr1* and itch-related transcripts. **a** Strategy for generating cKO mice (TBX20^fl/fl^;K14-Cre mice). The map shows the WT mouse *Tbx20* gene locus within genomic DNA, including the targeted exon 2 and 3, and the mutant *Tbx20* allele after Cre-loxP mediated deletion of these exons. Illustrations in **a** were created in BioRender. Meng, J. (2026) https://BioRender.com/q7b654v. **b** Phenotypic analysis of age-matched TBX20^fl/fl^;K14-Cre mice, their littermate controls (TBX20^fl/fl^), and age-matched WT mice for comparison. Genotyping of TBX20^fl/fl^;K14-Cre mice was performed by PCR to detect the *Tbx20* floxed allele and the K14-Cre transgene. Amplification of the *Tbx20* locus yielded a 223 bp product for the floxed allele and a 149 bp product for the WT allele, while amplification of the K14-Cre transgene produced a 450 bp amplicon. Immunohistochemical co-staining demonstrates that TBX20 is present in the skin of TBX20^fl/fl^ mice but absent in TBX20^fl/fl^;Avil-Cre mice, while K14 expression remains unchanged. Representative data and images are shown in the figures, and the experiments were independently repeated three times with similar results. Scale bar = 50 μm. **c** Cytokine array results on supernatant from cultured keratinocytes harvested from TBX20^fl/fl^;K14-Cre mice compared to their littermate controls (TBX20^fl/fl^). All the values are expressed as fold change relative to that of the TBX20^fl/fl^ + vehicle. Cells were stimulated with either BNP or its vehicle. Cytokine profiling was performed in a single experiment and is presented descriptively, as no independent biological replicates were included. Each cytokine was measured in duplicate spots, and signal intensity was calculated as the mean of the duplicate spots. Error bars reflect technical variation only; no inferential statistics were performed. **d** TBX20^fl/fl^;K14-Cre mice exhibited reduced BNP-induced scratching behaviours following *i.d*. injection in the cheek model compared to TBX20^fl/fl^ mice, including both total bouts and time-resolved bouts. *N* = 7 mice/group were analysed. **e** RT-qPCR analysis of itch-related transcripts from cheek skin after *i.d*. BNP injection; *n* = 3 mice/group analysed. Data presented as Mean ± SEM; a two-way ANOVA followed by Tukey’s multiple comparisons test (**d**, left panel) or a two tailed unpaired Student’s *t*-test (**d**, right panel and **e**) was used. *P < 0.05, **P < 0.01, ****P < 0.0001. Source data (**c**–**e**) are provided as a Source Data file.

To investigate the role of TBX20 in skin inflammation and itch, we generated mice with keratinocyte-specific TBX20 depletion by crossing TBX20^fl/fl^ mice with K14-Cre mice (Fig. 3a). The expression of Cre recombinase under the control of the K14 promoters leads to deletion of Exons 2 and 3 of the TBX20 gene specifically in keratinocytes, here named TBX20^fl/fl^;K14-Cre mice. These mice exhibited normal growth, survival, and overall phenotype compared to TBX20^fl/fl^ littermate controls and age-matched wild-type (WT) mice, making them suitable for behavioural assessments (Fig. 3b). TBX20 floxed alleles yielded 223 bp (floxed), 149 bp (wild-type), or both (heterozygotes). The K14-Cre transgene produced a 450 bp amplicon, allowing reliable identification of TBX20^fl/fl^;K14-Cre mice (Fig. 3b). TBX20 ablation in these mice was subsequently confirmed by immunofluorescence staining (Fig. 3b).

To assess whether TBX20 deletion affects the release of inflammatory and pruritogenic mediators, cytokine secretion profiles were assessed using the Proteome Profiler Murine XL Cytokine Array in primary keratinocytes from TBX20^fl/fl^;K14-Cre mice and TBX20^fl/fl^ littermate control mice following BNP (1 µM, 24 h), with conditioned media collected for analysis. In TBX20^fl/fl^ keratinocytes, BNP treatment appeared to upregulate multiple inflammatory and itch-associated mediators, including IL-10 (T_H_2 immunity and eosinophilic inflammation in allergic dermatitis^34^), MMP-3 (elevated in AD and promotes barrier disruption and inflammation^35^), VEGF (vascular endothelial growth factor driving vascular changes in AD lesions^36^), CXCL9 (T-cell recruitment and associated with chronic AD lesions^37^), IGFBP-3 (insulin-like growth factor-binding protein 3, associated with allergic disease^38^), and LDLR (low-density lipoprotein receptor regulating lipid metabolism and inflammatory homoeostasis^39^) (Fig. 3c). In contrast, TBX20 knockout appeared to reduce the BNP-induced release of these cytokines (Fig. 3c), suggesting that TBX20 mediates the BNP-stimulated secretion of inflammatory and pruritic factors from murine keratinocytes.

To verify that TBX20 mediates BNP-induced cytokine release from human keratinocytes, NHEK cells were cultured and infected with lentiviral TBX20 shRNA or non-targeted control viral particles prior to analysis of BNP-induced cytokines in the culture supernatant using Proteome Profiler Human XL Cytokine Array kit. Although the detection profiles differed from those observed in mouse samples, TBX20 knockdown, verified by immunoblot (Supplementary Fig. 4a), appeared to reduce multiple cytokines, including IL-19, IL-4, IL-24, IL-3 and IL-5 (Supplementary Fig. 4b), which are known to contribute to T_H_2-driven immune activation and keratinocyte-mediated cutaneous inflammation.

To determine whether keratinocyte-specific TBX20 deletion impacts acute itch in vivo, we intradermally (*i.d*.) injected BNP (10 µg) into the mice cheek, a method known to elicit itch-related scratching behaviour^11^, likely through direct activation of sensory neurons. Consistent with this mechanism, cultured trigeminal ganglionic neurons (TGNs) exhibited rapid calcium responses to BNP (1 µM), with a subset displaying delayed activation (Supplementary Fig. 5a).

TBX20^fl/fl^;K14-Cre mice exhibited significantly fewer scratching bouts compared to TBX20^fl/fl^ controls within 30 mins post-injection (Fig. 3d). Time-course analysis revealed a gradual decline in scratching between 0-25 mins, with statistically significant reductions observed between 15-20 mins (Fig. 3d). These findings indicate that TBX20 contributes to BNP-induced acute itch responses in vivo.

To further explore the molecular basis of this reduced itch response, we performed RT-qPCR to analyze gene expression in BNP-injected skin from TBX20^fl/fl^;K14-Cre and TBX20^fl/fl^ mice. TBX20^fl/fl^;K14-Cre mice showed significant downregulation of *Npr1* (Fig. 3e), consistent with the role of TBX20 in regulating *Npr1* transcription. In addition, several itch and inflammation-associated genes, including *Osm*, *Lcn2*, *Il4*, *Mcpt8*, *Il17a*, *Il19*, *Il20*, *Il24*, *Il27*, also significantly downregulated in knockout mice (Fig. 3e**)**. Additionally, RNAseq analysis indicated reduced *Il1α* and *Il1β* expression by FPKM analysis but not by count-based analysis, whereas Il33, *Il25*, *Tslp (Thymic Stromal Lymphopoietin)*, *Ccl5*, *Ccl27a* and *Ccl27b* were unchanged based on both count-based analysis and FPKM comparisons (Supplementary Fig. 5b). These transcriptional changes suggest that the diminished BNP-induced itch in TBX20^fl/fl^;K14-Cre mice may result, at least in part, from reduced expression of NPR1 and other itch- and inflammation-related genes.

To examine keratinocytes-sensory neuron crosstalk, keratinocytes isolated from TBX20^fl/fl^;K14-Cre and TBX20^fl/fl^ mice were stimulated with BNP for 24 hours, and the resulting conditioned media were applied to TGNs isolated from TBX20^fl/fl^ mice. Calcium imaging revealed that conditioned media from BNP-stimulated TBX20^fl/fl^;K14-Cre keratinocytes elicited significantly reduced peak of neuronal calcium responses compared with media from TBX20^fl/fl^ keratinocytes (Supplementary Fig. 6a–c). Because neurons were not directly exposed to BNP and conditioned media were generated under identical stimulation conditions, these differences likely reflect TBX20-dependent changes in keratinocyte-derived soluble factors, supporting a paracrine mechanism by which keratinocytes enhance sensory neuron excitability.

### LCN-2 acts downstream of TBX20-NPR1, and its knockout results in a deficit in MC903- and DNFB-induced itch response

**Fig. 4:**
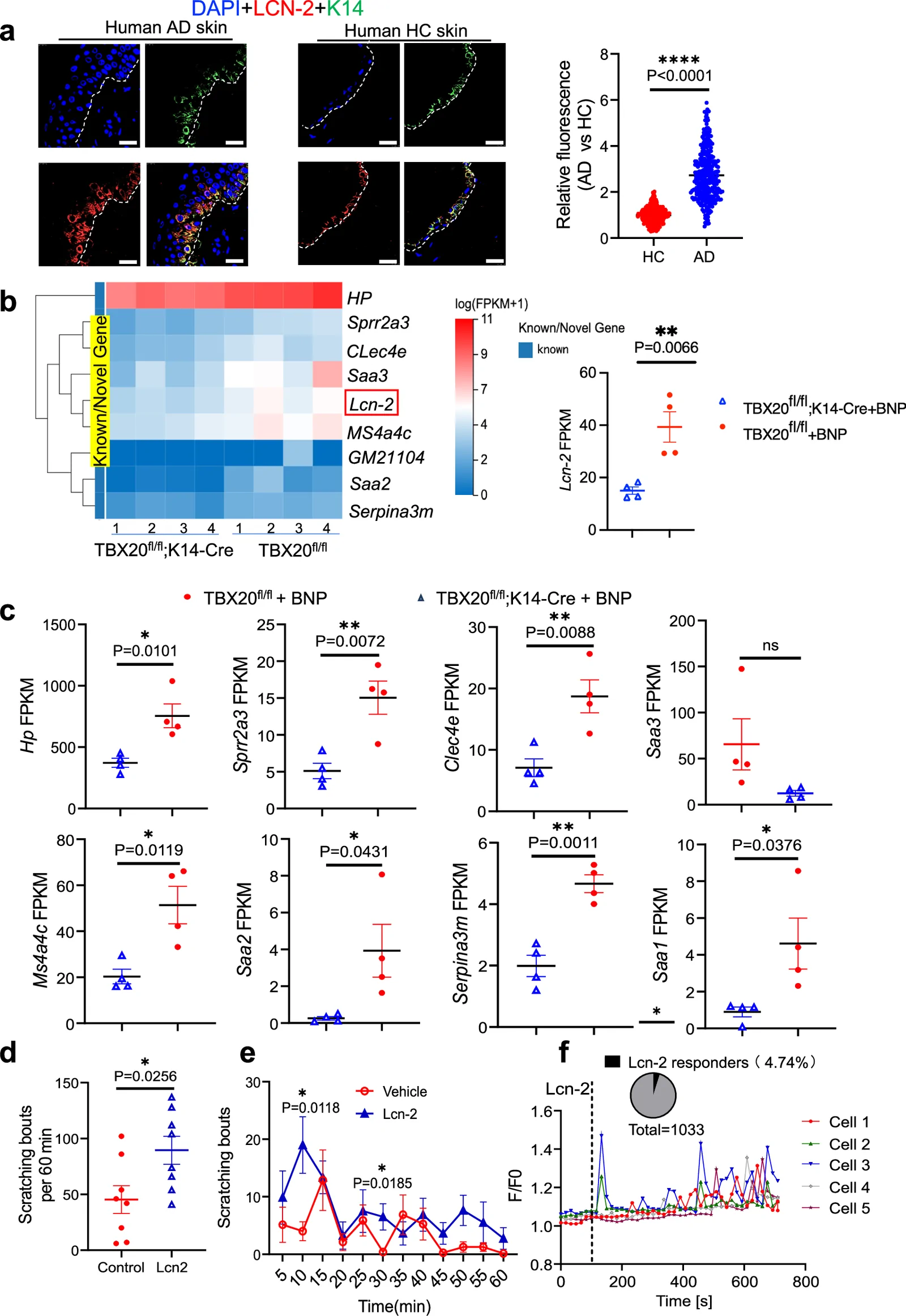
LCN-2 is upregulated in the human lesional AD skin; intradermal BNP injection-induced upregulation of *Lcn-2* is reduced in TBX20^fl/fl^; K14-Cre mice. LCN-2 induces scratching behaviours in the mouse cheek model. **a** Representative confocal immunofluorescence images and quantitation analysis of LCN-2 fluorescence intensity in skin samples from patients with lesional AD compared to HC. Fluorescence intensity was measured at the single-cell level from three independent donors, with cells nested within each experiment; individual cells are shown for visualization. Scale bar = 25 μm. **b** Heatmap of gene expression for known inflammatory and stress-related genes across samples. Rows represent individual genes, clustered by expression pattern, and columns represent *n* = 4 mice per strain: BNP-injected TBX20^fl/fl^; K14-Cre^+^ mice and TBX20^fl/fl^ mice. Red indicates high expression, and blue indicates low expression. FPKM values for *Lcn-2* are compared between the two groups. **c** Comparison of FPKM values for genes highlighted in the heatmap between BNP-injected TBX20^fl/fl^; K14-Cre mice and TBX20^fl/fl^ mice. *N* = 4 mice per strain. **d, e**. Analysis of scratching bouts in normal WT mice after intradermal injection of LCN-2 or vehicle on the cheek： (**d**) total scratching bouts and (**e**) time-resolved scratching behaviour (*n* = 8 mice/group). **f** Effect of LCN-2 on cultured murine TGNs from WT mice; representative traces for calcium imaging using Fluo-4 AM (time versus F/F0) following LCN-2 stimulation (break line indicated). Temporal subpopulation analysis is shown as a Venn diagram, with the curve representing the response profile of a single neuron. 1033 neurons were analysed in total. Data presented as Mean ± SEM; a two-tailed unpaired Student’s t-test (**a**–**e**) was used. ^ns^*P*>0.05, *P < 0.05, **P < 0.01, ****P < 0.0001. Source data (**a**–**f**) are provided as a Source Data file.

In our BNP-induced cheek itch model, *Lcn-2* transcription was significantly reduced in TBX20^fl/fl^;K14-Cre mice, indicating a potential regulatory link between BNP signalling and LCN-2 regulation. Supporting this hypothesis, immunohistochemical co-staining confocal images revealed a pronounced increase in LCN-2 expression in basal K14^+^-keratinocytes of human LAD skin compared with HC (Fig. 4a). In BNP-injected mouse cheek skin, keratinocyte-specific deletion of TBX20 significantly downregulated *Lcn-2* (Log2fc = -1.6; q-value = 0.0028) (Fig. 4b). Several inflammatory and stress-associated genes were also identified by count-based analysis, including *Hp* (log₂FC = −1.2; *q* = 0.0008), *Sprr2a3* (log₂FC = −1.8; *q* = 0.006), *Clec4e* (log₂FC = −1.7; *q* = 0.02), *Ms4a4c* (log₂FC = −1.6; *q* = 0.01), *Saa2* (log₂FC = −4.4; *q* = 0.0004), and *Serpina3m* (log₂FC = −1.4; *q* = 0.01). *Saa3* (log₂FC = −2.58; *q* = 0.006) and *GM21104* (log₂FC = −21.73; *q* = 2.65 × 10⁻⁶) were also decreased based on count-based analysis; however, their corresponding FPKM values did not show statistically significant differences (Fig. 4c and Supplementary Fig. 5b). In contrast, *Saa1* (log₂FC = −2.67; *q* = 0.07) was not significantly altered in the count-based analysis, although FPKM values indicated significant downregulation. *Hp*, *Saa2*, *Saa3*, *Clec4e*, *Ms4a4c*, *Sprr2a3*, and *serpina3m* have been reported to reflect innate immune activation, acute-phase inflammatory signalling, and compensatory epidermal barrier responses in skin disease; however, their roles in AD remain unclear^40–43^. In our dataset, these genes were downregulated, suggesting reduced innate immune and acute-phase responses.

Lcn-2 has been implicated in the chronic itch-scratch cycle^44^ and correlates with itch severity in psoriasis^45^; however, its role in the periphery and in AD remains poorly defined. Notably, functional interactions between Lcn-2 and BNP have been described in other pathological settings, such as sepsis-induced myocardial injury^46^, further supporting the existence of a TBX20-BNP-NPR1 axis in the regulation of itch.

We next investigated whether Lcn-2 directly contributes to peripheral itch. Intradermal injection of recombinant Lcn-2 into the mouse cheek induced a significant increase in itch-like behaviour compared with vehicle controls (Fig. 4d), with scratching peaking approximately 10 min post-injection (Fig. 4e). To determine whether Lcn-2 can directly activate sensory neurons, we applied Lcn-2 to cultured murine TGNs, which elicited calcium influx in a distinct subset of neurons (~4.74%) (Fig. 4f).

**Fig. 5:**
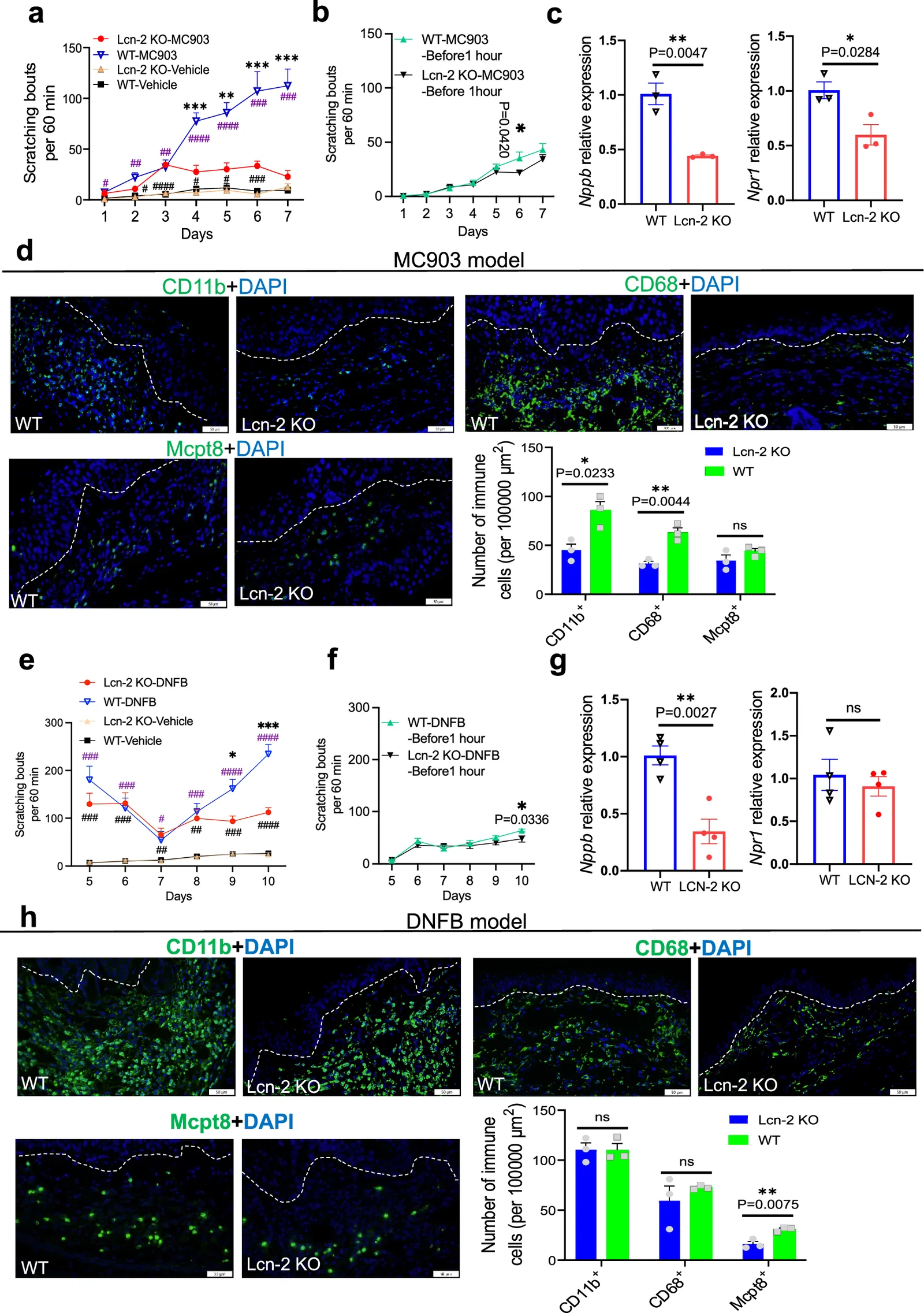
MC903- and DNFB-induced itch is reduced in TBX20^fl/fl^;K14-Cre mice, accompanied by decreased inflammatory cell infiltration. Scratching bouts in Lcn-2 KO mice and WT controls induced by MC903 or vehicle (**a**, WT-MC903: *n* = 7 mice/group; Lcn-2 KO-MC903: *n* = 7 mice/group) or baseline scratching monitored one hour prior to MC903 administration (**b**, *n* = 6 mice/group) in the ear model; for (**a**), ^*^, WT-MC903 *vs* Lcn-2 KO-MC903; black #, Lcn-2 KO-MC903 *vs* Lcn-2 KO-vehicle； purple #, WT-MC903 *vs* WT-vehicle. for (**b**), * is indicated for day 6. RT-qPCR quantification of *Nppb* and *Npr1* transcription in MC903-treated ear skin from WT and Lcn-2 KO mice (**c**) and analysis of immune cell infiltration in the MC903-ear (**d**); representative images shown. Scale bar = 50 μm. *N* = 3 mice/group were analysed. Scratching bouts induced by DNFB or vehicle (**e**, *n* = 6 mice/group) or baseline scratching monitored one hour prior to DNFB application (**f**, *n* = 6 mice/group) in the ear of Lcn-2 KO mice and WT controls; for (**e**), ^*^, WT-DNFB *vs* Lcn-2KO-DNFB; black #, Lcn-2 KO-DNFB *vs* Lcn-2 KO-vehicle; purple #, WT-DNFB *vs* WT-vehicle. for **f**, * is indicated for day 10. **g** RT-qPCR quantification of *Nppb* and *Npr1* transcription in DNFB-treated ear skin from WT and Lcn-2 KO mice. *N* = 4 mice/group were analysed. **h** Immune cell infiltration in DNFB-treated ear skin (*n* = 3 mice/group); representative images shown. Scale bar = 50 μm. Data presented as Mean ± SEM; a two tailed unpaired Student’s *t*-test (**a**–**h**) was used. ^ns^*P* > 0.05, *P < 0.05, **P < 0.01, ***P < 0.001. Source data (**a**–**h**) are provided as a Source Data file.

Consistent with these findings, Lcn-2 knockout significantly reduced MC903-induced ear scratching compared with WT mice, without affecting the minimal scratching observed in vehicle-treated controls (Fig. 5a). Baseline scratching, assessed one hour before MC903 application, was minimally affected (Fig. 5b). In left ear skin, transcription of *Nppb* and *Npr1* was reduced in Lcn-2 knockout mice (Fig. 5c), indicating that Lcn-2 positively regulates the Nppb–Npr1 axis and NPR1 signalling. Lcn-2 deficiency further reduced MC903-induced infiltration of CD11b^+^ and CD68^+^ cells, with a nonsignificant reduction in Mcpt8^+^ basophils (Fig. 5d). These markers were prioritized to capture key pathogenic immune populations. CD68 labels macrophages that contribute to cytokine production and tissue inflammation; CD11b identifies a broad range of myeloid cells, including monocytes and neutrophils, reflecting the innate immune infiltration; and MCPT8 specifically labels basophils, which are central drivers of MC903-induced skin inflammation^47^.

Similarly, Lcn-2 knockout significantly suppressed DNFB-induced itch compared with WT mice, without affecting scratching in vehicle-treated controls (Fig. 5e). Baseline scratching monitored one hour prior to DNFB application remained minimal (Fig. 5f). *Nppb* transcription in the left ear was significantly reduced, whereas *Npr1* exhibited a downward trend (Fig. 5g). In this model, Lcn-2 deficiency did not significantly reduce CD11b^+^ and CD68^+^ cell infiltration but significantly decreased basophils numbers (Fig. 5h). Dissection time points for both models were selected to coincide with their fully developed inflammatory states^8,48,49^.

Collectively, these findings suggest that Lcn-2 may act as a downstream effector of the TBX20-NPR1 pathway, contributing to itch propagation and promoting monocyte/macrophage infiltration in AD lesional skin, with model-dependent effects on basophils. Reduced itch, accompanied by decreased *Nppb* and *Npr1* expression, supports a role for Lcn-2 in coupling cutaneous inflammation to sensory neuron activation.

### TBX20 knockout in keratinocytes reduces itch and inflammation in the MC903-induced AD-like model

**Fig. 6:**
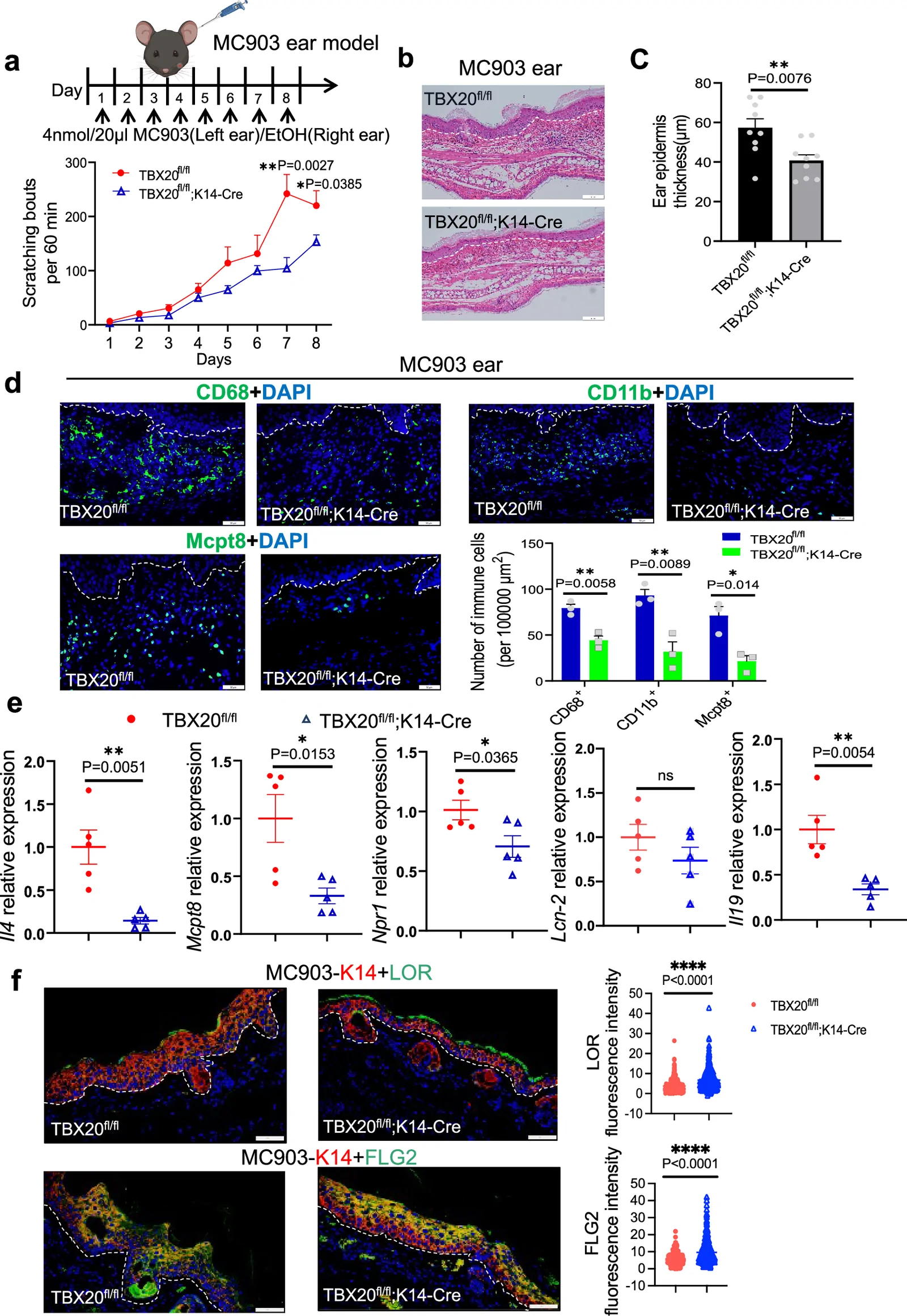
TBX20^fl/fl^; K14-Cre mice exhibited reduced itch, immune cell infiltration, and skin hyperplasia, along with decreased expression of itch- and inflammation-related transcripts and increased levels of skin barrier proteins in the MC903-model. **a** Schematic of the establishment of the MC903-induced ear model. Itch behaviour induced by MC903 was significantly reduced in TBX20^fl/fl^; K14-Cre mice compared to littermate TBX20^fl/fl^ control mice. *N* = 12 mice/group. Illustrations in (**a**) were created in BioRender. Meng, J. (2026) https://BioRender.com/q7b654v. **b** Representative H&E staining of MC903-treated ear skin from the MC903-induced model. The broken line marks the epidermal-dermal junction on the side of the ear where MC903 was applied. Scale bar = 100 μm. **c** Measurement of ear epidermis thickness on the side of the ear where MC903 was applied. *N* = 9 mice per strain. **d** Immunohistochemical staining of MC903-induced ear skin for CD11b, Mcpt8, and CD68. Bar chart depicts immune cell infiltration in MC903-induced ear skin (*n* = 3 mice/group). Representative images are shown. Scale bar = 50 μm. **e** RT-qPCR analysis of gene transcripts in the MC903-induced ear skin of TBX20^fl/fl^; K14-Cre mice compared to TBX20^fl/fl^ control mice. *N* = 5 mice/group. **f** Representative immunohistochemical staining of skin barrier proteins LOR and FLG2 (K14 is the keratinocyte-specific marker) in MC903-ear skin samples from TBX20^fl/fl^; K14-Cre when compared to TBX20^fl/fl^ control mice. Statistical analysis was performed on biological replicates (*n* = 3), with cells nested within each experiment; individual cells are shown for visualization. Scale bar = 50 μm. Data are mean ± SEM; a two-tailed unpaired Student’s t-test (**a**, **c**–**f**) was used. ^ns^*P* > 0.05, *P < 0.05, **P < 0.01, ****P < 0.0001. Source data (**a**, **c**–**f**) are provided as a Source Data file.

To investigate the effect of TBX20 knockout on itch response in AD, TBX20^fl/fl^; K14-Cre mice were compared with littermate TBX20^fl/fl^ control mice. TBX20^fl/fl^; K14-Cre mice exhibited significantly reduced itch on days 7 and 8 following MC903 treatment (Fig. 6a). Consistently, MC903-treated ear skin from these mice showed decreased epidermal thickness (Fig. 6b, c) and reduced immune cell infiltration, as evidenced by fewer CD68^+^, CD11b^+^, and MCPT8^+^ cells (Fig. 6d).

In addition, infiltration of CD4^+^ T cells was also decreased (Supplementary Fig. 7a). Because T_H_1, T_H_2, and T_H_17 cells are functional subsets within the CD4⁺ T-cell compartment, and most classical regulatory T cells (Tregs) are also CD4⁺, CD4 serves as a foundational marker for assessing these populations^50^. Together, these findings highlight a key role of TBX20 in regulating skin-derived itch and cutaneous inflammation in AD.

RT-qPCR analysis further confirmed downregulation of *Npr1* transcripts in MC903-treated ear skin of TBX20^fl/fl^; K14-Cre mice. Expression of *Mcpt8*, *Il4*, *Il19* (Fig. 6e) as well as *Il13* and *Periostin* (Supplementary Fig. 7b**)** was also reduced. Although *Lcn-2, Tslp, and Il31* expression trended downward (Fig. 6e and Supplementary Fig. 7b), this change did not reach statistical significance. The lack of significant *Lcn-2* downregulation in the MC903 model may reflect context-dependent regulation or compensatory signalling pathways that mitigate the effect of TBX20 loss in this specific inflammatory setting.

To assess the impact of keratinocyte-specific TBX20 knockout on skin barrier integrity, we analysed the expression of barrier-associated proteins LOR (Loricrin) and FLG2 (Filaggrin) in MC903-treated ear samples from TBX20^fl/fl^; K14-Cre and TBX20^fl/fl^ mice using immunohistochemistry. Quantification of fluorescence intensity revealed that both LOR and FLG2 were significantly upregulated in MC903-treated TBX20^fl/fl^; K14-Cre compared to TBX20^fl/fl^ mice (Fig. 6f).

Together, these results indicate that keratinocyte-TBX20 knockout downregulates NPR1 and other AD-related genes while enhancing skin barrier function.

### TBX20 overexpression via AAV-mediated gene delivery and its suppression through NRG1 application both modulate itch responses in AD

**Fig. 7:**
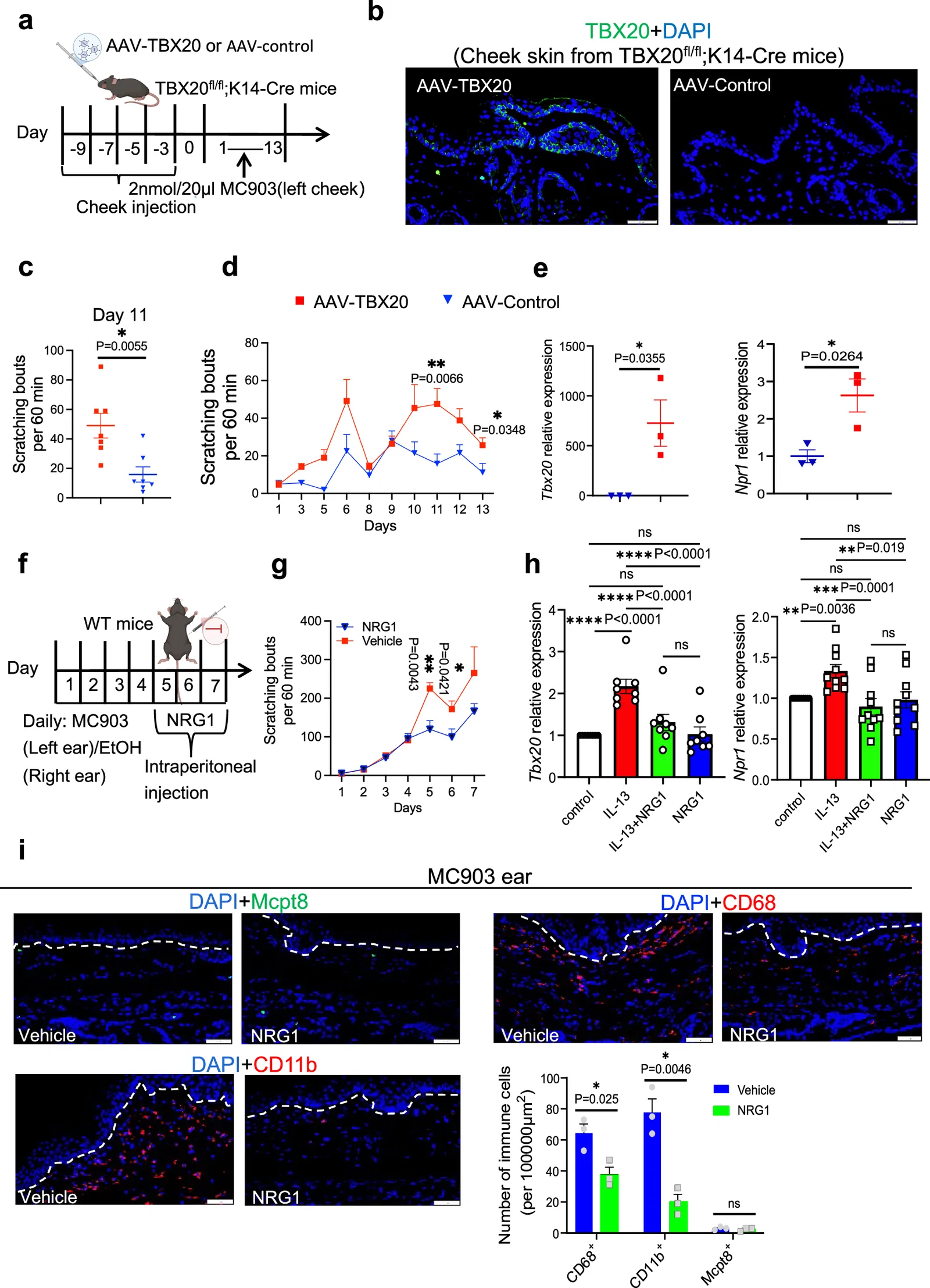
Rescuing TBX20 expression by AAV-mediated gene delivery restored itch response in TBX20^fl/fl^; Avil-Cre mice, while NRG1 inhibited TBX20 expression, reducing MC903-induced itch and immune cell infiltration in the ear model. **a** Schematic of the AAV-mediated TBX20 expression strategy in TBX20^fl/fl^;K14-Cre mice in the MC903-induced cheek model. AAV-Control is the negative control for AAV-TBX20. **b** Immunofluorescence images of TBX20 in the cheek skin following AAV-TBX20 or AAV-Control administration in the skin of TBX20^fl/fl^; K14-Cre mice, harvested on day 13. Experiments were independently repeated three times with similar results. Representative data and images are shown in the figures. Scale bar = 50 μm. Comparison of scratching bouts between AAV-TBX20 and AAV-Control injected TBX20^fl/fl^; K14-Cre mice in the MC903 cheek model. **c** Total number of scratching bouts on day 11. **d** Time-resolved analysis of scratching behaviour. *N* = 7 mice/group. **e** RT-qPCR analysis of *Tbx20* and *Npr1* transcript levels on MC903-cheek skin samples comparing AAV-TBX20 and AAV-Control injected TBX20^fl/fl^; K14-Cre mice (*n* = 3 mice/group). **f** Strategy of NRG1 administration in WT mice, followed by the establishment of the MC903-induced ear model. **g** Total number of scratching bouts in the MC903 ear model, comparing NRG1- and vehicle- treated WT mice (*n* = 5 mice/group). **h** RT-qPCR analysis of the effect of NRG1 on *TBX20* and *NPR1* transcripts in IL-13-stimulated human primary keratinocytes (NHEK) cells. *N* = 8 independent cultures for **h** (left) and *n* = 10 for **h** (right). **i** Representative immunohistochemistry images of ear skin from the MC903-induced model following NRG1 treatment, showing infiltration of Mcpt8^+^, CD68^+^ and CD11b^+^ cells. Bar-charts quantify immune cell infiltration in MC903-treated ear skin (*n* = 3 mice/group). Scale bar = 50 μm. Illustrations in (**a**) and (**f**) were created in BioRender. Meng, J. (2026) https://BioRender.com/q7b654v. Data presented as Mean ± SEM; a two-tailed unpaired Student’s t-test (**c**–**g**, and **i**) or 2-way ANOVA followed by Bonferroni’s multiple comparisons test (**h**) was used. ^ns^*P* > 0.05, *P < 0.05, ^**^P < 0.01, ***P < 0.001, ****P<0.0001. Source data (**c**, **d**–**i**) are provided as a Source Data file.

To investigate the selectivity of TBX20 function in itch, we employed AAV-mediated gene delivery to locally restore TBX20 expression in the skin of TBX20^fl/fl^; K14-Cre mice. Mice were injected in the cheek with either AAV-TBX20 viral particles (AAV-TBX20 group) or an equal amount of control virus (AAV-control group) every other day, for a total of four injections. Three days following the final injection, MC903 (2 nmol/20 μL) was applied to the virus-injected cheek skin in both groups. Scratching behaviour was recorded for one hour daily over 13 consecutive days (Fig. 7a). Immunofluorescence staining confirmed the expression of TBX20 in cheek skin of the AAV-TBX20 group (Fig. 7b). Scratching bouts were significantly increased in the AAV-TBX20 group compared with the AAV-control group. This increase was evident on day 11 (Fig. 7c) and remained significantly elevated on day 13 (Fig. 7d). RT-qPCR analysis of cheek skin harvested on day 13 revealed elevated transcription levels of both *Tbx20* and *Npr1* in the AAV-TBX20 group relative to the AAV-control group (Fig. 7e). These findings suggest that AAV-mediated TBX20 gene expression in TBX20^fl/fl^; K14-Cre mice restored *Npr1* expression and may contribute to the increased scratching behaviours observed.

To further investigate the role of TBX20 in AD-associated itch, we employed pharmacological inhibition of TBX20 using Neuregulin-1 (NRG1), a member of the paracrine epidermal growth factor family previously shown to suppress Tbx20 expression in a dose-dependent manner^22^. The MC903-induced itch response was monitored from Day 1 to Day 4. On Day 5, mice were randomly assigned to receive either an intraperitoneal (*i.p*.) injection of NRG1 or PBS (vehicle). Scratching behaviour was recorded daily following MC903 application through Day 7 (Fig. 7f). Mice treated with NRG1 exhibited reduced scratching bouts compared to vehicle-injected controls, with statistically significant differences observed on days 5–6 (Fig. 7g).

To assess the effect of NGR1 on TBX20/NPR1 expression in response to itch-related stimuli, we conducted in vitro experiments using cultured NHEKs. Cells were incubated for 24 hours with vehicle, IL-13, NRG1, or a combination of IL-13 and NRG1. RT-qPCR analysis revealed that IL-13 alone significantly upregulated *TBX20* and *NPR1* transcription, while co-treatment with NRG1 significantly attenuated this IL-13-induced transcriptional increase (Fig. 7h).

To evaluate the impact of NRG1 on inflammatory cell infiltration, ear skin from MC903-induced mouse models treated with NRG1 or vehicle was collected and analysed by immunohistochemistry. NRG1 treatment significantly reduced infiltration of CD68^+^ and CD11b^+^ cells, but not MCPT8^+^ cells, compared to the vehicle group (Fig. 7i).

Collectively, these TBX20 rescue and inhibition experiments highlight its role in regulating NPR1 expression, immune cell infiltration, and itch signalling.

### Knocking out TBX20 in mouse skin can alleviate itch and skin inflammation in DNFB models

**Fig. 8:**
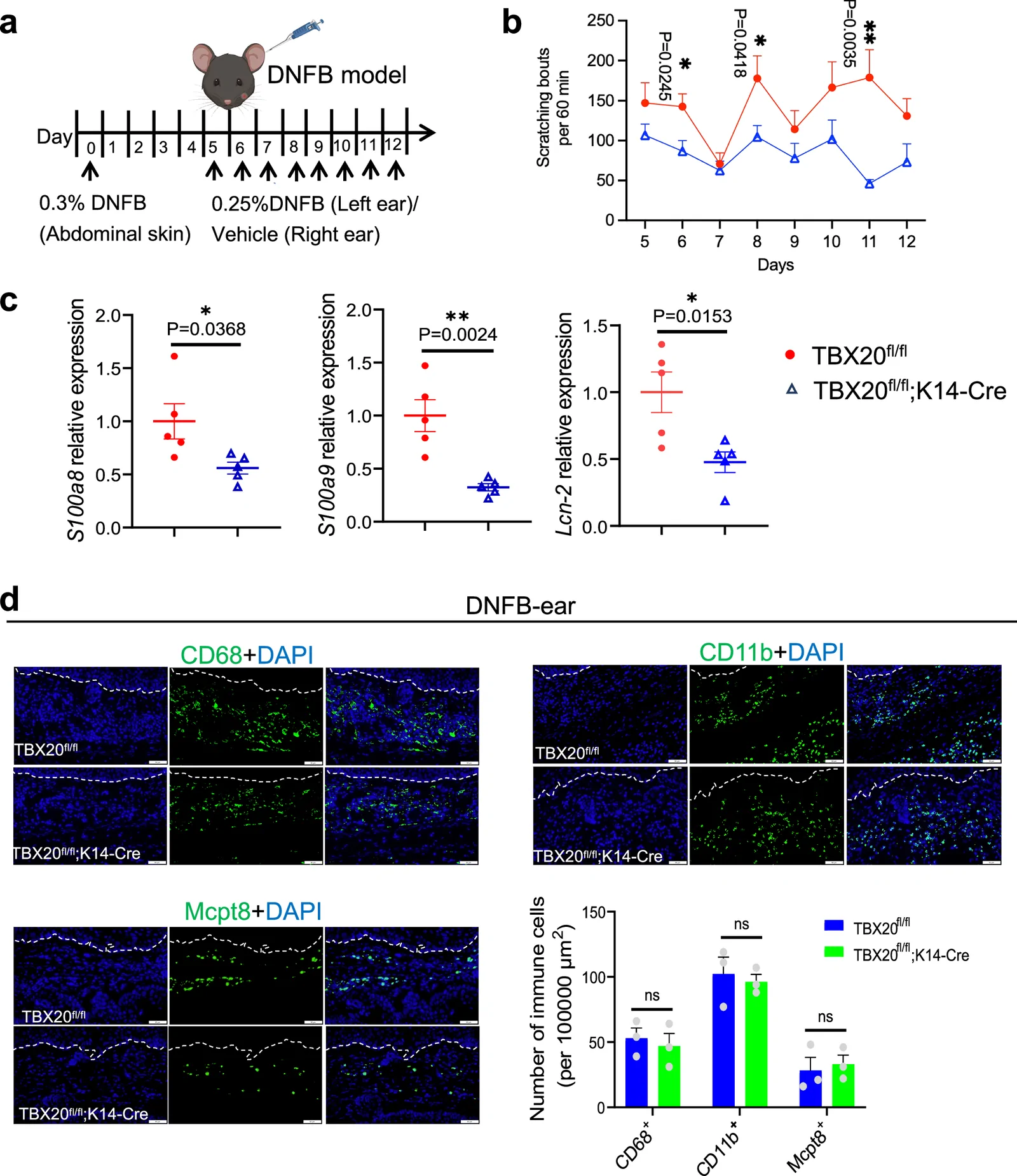
TBX20^fl/fl^;K14-Cre mice exhibited reduced itch and decreased itch- and inflammation-related transcripts, but not reduced immune cell infiltration, in the DNFB-induced ear model. Schematic representation of the DNFB-induced ear model (**a**) and DNFB-induced itch in TBX20^fl/fl^; K14-Cre mice was significantly lower than in TBX20^fl/fl^ mice (**b**). *N* = 6 mice/group. Statistical significance in (**b**) is indicated on days 6, 8, and 11. **c** RT-qPCR analysis of gene expression in DNFB-ear skin samples from TBX20^fl/fl^; K14-Cre and TBX20^fl/fl^ mice in the DNFB-ear model (*n* = 5 mice/group). Illustrations in (**a**) were created in BioRender. Meng, J. (2026) https://BioRender.com/q7b654v. **d** Representative immunohistochemical staining analysis of CD68^+^, CD11b^+^, and Mcpt8^+^ cell infiltration in DNFB-ear skin from TBX20^fl/fl^; K14-Cre mice compared to TBX20^fl/fl^ control mice. Bar chart depicts analysis of immunopositive cells (*n* = 3 mice/group). Scale bar = 50 μm. Data are mean ± SEM; a two-tailed unpaired Student’s *t*-test was used. ^ns^*P *> 0.05, *P < 0.05, **P < 0.01. Source data (**b**–**d**) are provided as a Source Data file.

We further investigated the role of TBX20 in chronic itch using a model of ACD, induced by DNFB application to the ear, in TBX20^fl/fl^; K14-Cre and TBX20^fl/fl^ mice (Fig. 8a**)**. TBX20^fl/fl^; K14-Cre mice exhibited significantly reduced scratching bouts compared to TBX20^fl/fl^ controls on days 6, 8, and 11 of the DNFB challenge phase (Fig. 8b). RT-qPCR analysis revealed downregulation of the skin damage markers *S100a8* and *S100a9*, as well as decreased expression of *Lcn-2* transcripts in the skin of TBX20^fl/fl^; K14-Cre mice compared to TBX20^fl/fl^ controls (Fig. 8c). However, immune cell infiltration, including CD68^+^, CD11b^+^ and Mcpt8^+^ cells, was not altered by keratinocyte-specific TBX20 knockout (Fig. 8d). These findings suggest that TBX20 deletion in keratinocytes modulates the itch response without significantly affecting skin inflammation in the ACD model, likely due to the differing immune mechanisms involved in AD versus ACD^51^.

### Knocking out TBX20 in sensory neurons improves itch and skin inflammation in the MC903-induced AD mouse model

Sensory neurons express both TBX20 and NPR1^27^, but the connection between neuronal TBX20 and itch has not been well studied. In paraffin-embedded human dorsal root ganglion (DRG) sections, RNAscope revealed both distinct and overlapping transcription of *TBX20* and *NPR1* (Supplementary Fig. 8a), prompting further investigation of TBX20 function in sensory neurons. *NPR1*^+^-only cells accounted for approximately 10% of total cells, while *NPR1*⁺*TBX20*⁺ co-expressing cells represented an additional ~10% of total cells (Supplementary Fig. 8a). The quality of the RNAscope assay was validated using positive and negative control probes (Supplementary Fig. 8b).

**Fig. 9:**
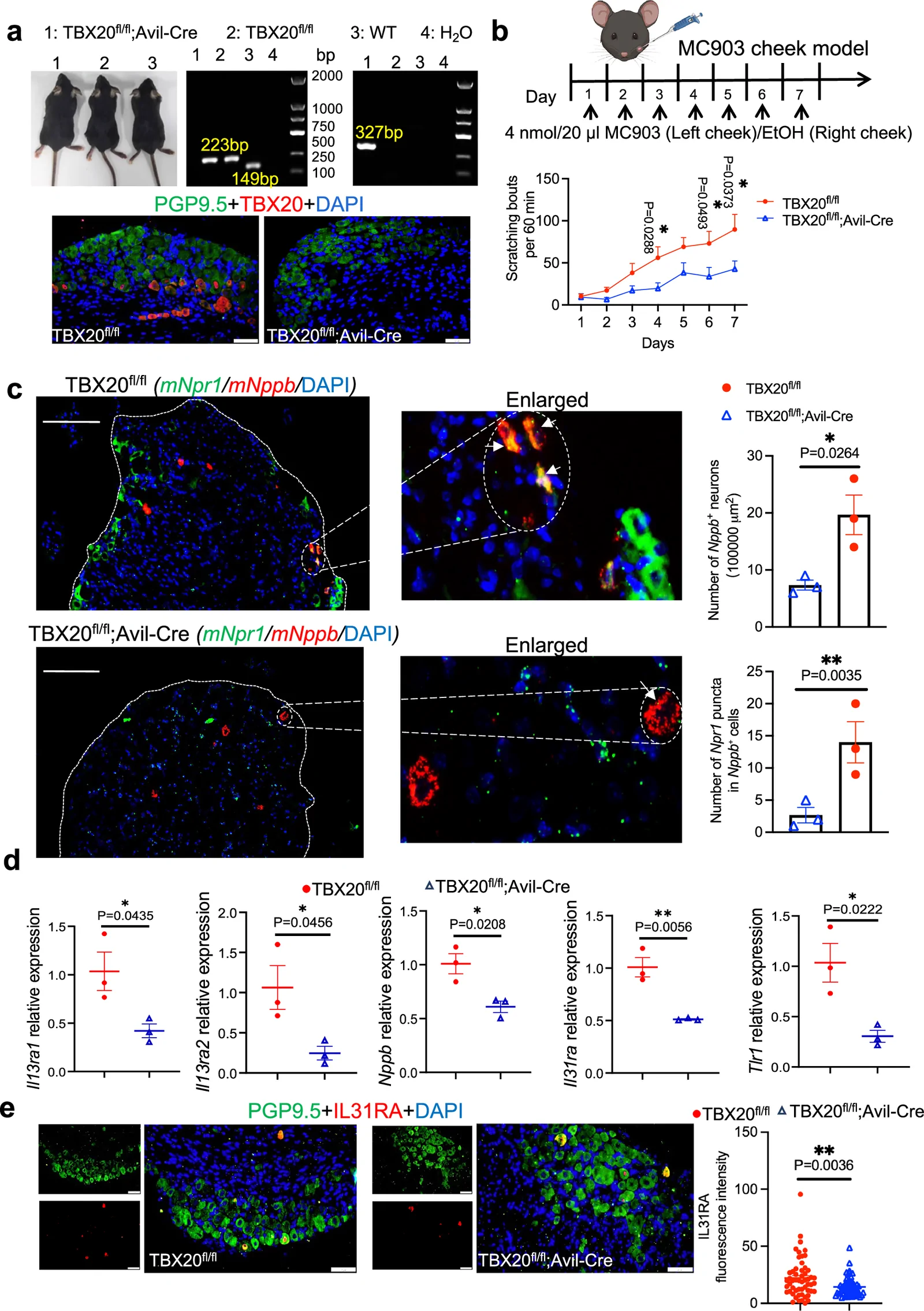
TBX20^fl/fl^; Avil-Cre mice exhibited reduced itch, decreased expression of *Npr1* and *Nppb*, and downregulation of itch- and inflammation-related genes and receptors in ipsilateral trigeminal ganglia in the MC903-induced cheek model. **a** TBX20^fl/fl^;Avil-Cre mice were genotyped (223 bp floxed, 149 bp WT; Avil-Cre 327 bp), with heterozygotes showing both the floxed and WT *Tbx20* bands. Representative immunohistochemical staining showing co-staining of TBX20 and PGP9.5 in trigeminal ganglia (TG) of TBX20^fl/fl^; Avil-Cre mice and TBX20^fl/fl^ mice. Scale bar = 50 μm. **b** Schematic of the MC903-induced cheek model. Itch behaviour induced by MC903 was significantly reduced in TBX20^fl/fl^; Avil -Cre mice compared to TBX20^fl/fl^ control mice. *N* = 7 mice/group. Illustrations in **b** were created in BioRender. Meng, J. (2026) https://BioRender.com/q7b654v. **c**. Representative RNAscope images of *Npr1* and *Nppb* transcripts in the ipsilateral TG from the MC903-cheek model. Oval circles highlight the same neurons at low magnification in the corresponding enlarged images; arrowheads indicate neurons expressing *Npr1* and *Nppb* puncta. Quantification shows the number of *Nppb*^+^ neurons per 100,000 μm^2^ of TG focal plane and the number of *Npr1* puncta within *Nppb*^+^ neurons. *N* = 3 mice/group. Scale bar = 100 μm. **d** RT-qPCR analysis of  gene transcripts the ipsilateral TG of TBX20^fl/fl^; Avil-Cre mice compared to TBX20^fl/fl^ mice following MC903-cheek modelling (*n* = 3 mice/strain). **e**. Representative immunohistochemical co-staining of PGP9.5 + IL-31RA in the ipsilateral TG from the MC903-cheek model of TBX20^fl/fl^; Avil-Cre mice compared to TBX20^fl/fl^ control mice. Statistical analysis was performed on biological replicates (*n* = 3), with cells nested within each experiment; individual cells are shown for visualization. Scale bar = 50 μm. Data are mean ± SEM; a two-tailed unpaired Student’s *t*-test was used. *P < 0.05, **P < 0.01. Source data (**b**–**e**) are provided as a Source Data file.

To explore this link, we generated sensory neuron-specific TBX20 cKO mice (TBX20^fl/fl^; Avil-Cre), by crossing TBX20^fl/fl^ mice with Advillin (Avil)-Cre mice, using a strategy similar to that employed for producing TBX20^fl/fl^; K14-Cre mice (c.f. Figs. 3a and 9a). TBX20^fl/fl^; Avil-Cre mice displayed normal phenotypes and were correctly genotyped (Fig. 9a). Immunohistochemical staining of trigeminal ganglion (TG) from TBX20^fl/fl^ mice revealed TBX20 expression in a small subset of ipsilateral TG neurons, marked by PGP9.5. In contrast, TBX20 expression was absent in the TG of TBX20^fl/fl^; Avil-Cre mice (Fig. 9a), confirming efficient depletion of TBX20 from sensory neurons. This finding is consistent with the known sensory neuron-specific expression of Avillin across all somatosensory neuron subtypes^52^. In the MC903 model, TBX20^fl/fl^; Avil-Cre mice exhibited significantly reduced scratching behaviour compared to TBX20^fl/fl^ controls, particularly on days 4, 6 and 7 (Fig. 9b). Notably, this reduction occurred earlier than in skin-specific TBX20 knockout mice (c.f. Fig. 6a).

To examine whether TBX20 regulates *Npr1* and *Nppb* expression in neurons, we performed RNAscope on ipsilateral TG from MC903-treated mice, with assay quality validated using positive and negative control probes (Supplementary Fig. 9). In TBX20^fl/fl^ controls, a higher number of *Nppb*^+^ cells was observed, whereas this number was significantly reduced in TBX20^fl/fl^; Avil-Cre mice (Fig. 9c). *Npr1* transcripts were detected in *Nppb*^*+*^ neurons, and the number of puncta in these neurons was significantly decreased in TBX20^fl/fl^; Avil-Cre mice compared with TBX20^fl/fl^ mice (Fig. 9c). These results suggest that TBX20 in our model positively regulates itch-related gene expression in sensory neurons.

RT-qPCR analysis of in the ipsilateral TG further revealed significant reductions in the expression of itch- and inflammation-associated genes in the MC903-treated TBX20^fl/fl^; Avil-Cre mice compared with controls. These included *Il13ra1* and *Il13ra2*, key receptors mediating IL-13–driven inflammation in AD^53^; *Nppb*, a critical neuroimmune mediator of itch and inflammation, known to be upregulated in AD lesion^13^; *Tlr1*, a microbial sensor that contributes to skin inflammation responses and is upregulated in lesions with high house dust mite sensitization^54^; and *Il31ra*, which mediates IL-31-driven itch and type 2 inflammation^13^ (Fig. 9d).

These findings suggest that TBX20 may transcriptionally regulate itch- and inflammation-related modulators in the AD model, influencing both cytokine-receptor signalling and neuroimmune pathways. This points to a potential upstream role for TBX20 in coordinating the molecular networks that drive cutaneous inflammation and pruritus. Consistently, immunofluorescence staining showed reduced protein levels of IL31RA in the ipsilateral TG of TBX20^fl/fl^; Avil-Cre mice compared to controls (Fig. 9e).

**Fig. 10:**
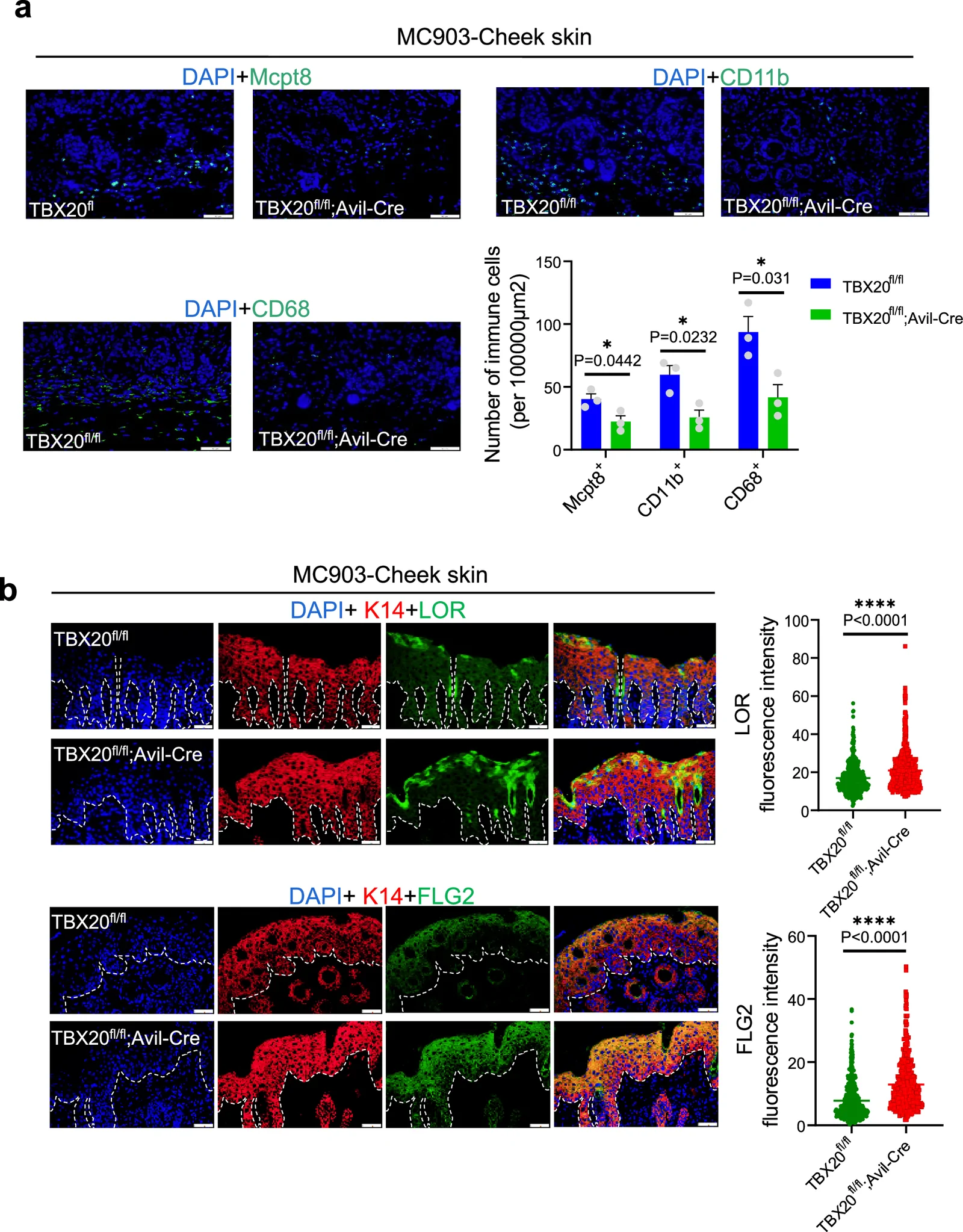
TBX20^fl/fl^; Avil-Cre mice exhibited reduced immune cell infiltration and increased skin barrier protein expression in the cheek skin of the MC903-cheek model. **a** Representative immunohistochemical staining of CD11b, Mcpt8, and CD68 in MC903-cheek skin samples from the MC903-cheek model, comparing TBX20^fl/fl^;Avil-Cre mice and TBX20^fl/fl^ control mice (*n* = 3 mice/group). A bar chart displays the quantitative analysis of immune cell infiltration between two groups. Scale bar = 50 μm. **b** Representative immunohistochemical staining for skin barrier proteins LOR and FLG2, with K14 as a keratinocyte-specific marker. MC903-cheek skin samples of TBX20^fl/fl^; Avil-Cre mice were compared to TBX20^fl/fl^ control mice. Statistical analysis was performed on biological replicates (*n* = 3), with cells nested within each experiment; individual cells are shown for visualization. Scale bar = 50 μm. Data are mean ± SEM; a two-tailed unpaired Student’s *t*-test was used. *P < 0.05, ****P < 0.0001. Source data (**a**, **b**) are provided as a Source Data file.

Moreover, on day 7 following MC903 treatment, TBX20^fl/fl^; Avil-Cre mice exhibited significantly reduced immune cell infiltration in the cheek skin, as evidenced by decreased numbers of Mcpt8^+^, CD11b^+^, and CD68^+^ cells (Fig. 10a). Additionally, expression of skin barrier proteins LOR and FLG2 was significantly increased in TBX20^fl/fl^; Avil-Cre mice (Fig. 10b). Together, these results highlight a key role for neuronal TBX20 in modulating itch and cutaneous inflammation, potentially through BNP-NPR1 signaling pathways.

## Discussion

Our approach involves leveraging clinical samples, cell-based assays, and generating specific transcription-factor-deficient transgenic animal models to investigate previously unreported transcriptional mechanisms driving itch. Using this strategy, we investigated the role of TBX20 in keratinocytes and sensory neurons by selectively knocking out the gene in models of AD and ACD. Complementary gain- and loss-of-function studies revealed that TBX20 is a critical regulator of itch, immune cell infiltration, and epidermal barrier integrity, identifying it as a promising therapeutic target in chronic pruritic conditions.

BNP is a critical neuropeptide transmitting itch at peripheral and central levels. Comparative transcriptomic analysis across itch and pain models identified BNP as a conserved biomarker in chronic itch, but not chronic pain^12^. Despite this, no therapeutics targeting the BNP pathway have been successfully developed. Previous studies have identified that elevated levels of BNP and NPR1 in the pruritic epidermis correlated with itch severity^13,55^. Dysregulation within the BNP-NPR1 signalling network can lead to an exaggerated immune response, heightened sensitivity to environmental triggers, and compromised skin integrity, all of which collectively drive the development and persistence of chronic itch symptoms^5^. Antagonising NPR1 has shown efficacy in reducing itch behaviour in both acute and chronic inflammatory dermatitis mouse models^9^, underscoring its potential as a therapeutic target^56^. However, the underlying mechanisms driving the overexpression of NPR1 in itch-related diseases and amplifying BNP-NPR1 signalling remained unclear.

Our study highlights the cardiovascular-related disease gene TBX20 as the crucial regulator of the BNP-NPR1 pathway in skin and peripheral neuronal systems under chronic itch conditions. *Tbx20* ChIP peak calling analysis followed by ChIP-qPCR revealed that the NPR1 gene contains a TBX20 conserved binding domain and that BNP promotes TBX20 interaction with the NPR1 promoter in keratinocytes, which in turn regulates NPR1 transcription. Ratiometric dual luciferase reporter assay further confirmed that BNP significantly enhanced TBX20-mediated activation of the Wt NPR1 promoter, whereas this effect was abolished in the mutant construct lacking functional TBX20 binding sites. This upregulation might amplify itch signalling, trigger immune activation, disrupt skin barrier function, and promote inflammation—core elements in the development and persistence of chronic itch^57^.

NPR1 and TBX20 transcripts were markedly increased in the epidermis of lesional AD skin and tape-striped skin samples, and the number of co-expressing cells detected by RNAscope was elevated^16^, implicating keratinocyte-specific TBX20 as key drivers of itch in these contexts. Using TBX20^fl/fl^; K14-Cre mice, a significant reduction of itch response and inflammation was observed across multiple models: BNP-induced itch, AD-like itch induced by MC903, and ACD-related itch triggered by DNFB, associated with downregulation of *Npr1* transcripts. These findings suggest a direct correlation between TBX20 expression in skin cells and the severity of chronic itch. Notably, TBX20 has been proposed to be a putative target of a transcription factor ΔNp63 in AD, which, when overexpressed in the epidermis, induces skin lesions and pruritus, and activates IL-31 and IL-33 pathways^58^. The reduction of itch observed in the MC903-induced model in TBX20^fl/fl^; K14-Cre mice was accompanied by downregulation of *Npr1*, as well as *Mcpt8*, *Il4*, *Il19*, *Il13*, and *Periostin* in the skin, further suggesting that TBX20 may regulate *Npr1* expression within the inflammatory network driving AD.

cKO of TBX20 in keratinocytes affected BNP induced transcription of *HP, Sprr2a3, Clec4e, Saa2*, *Lcn2*, *MS4a4c*, as well as *Serpina3m* in skin. Among these, Lcn-2 has been shown to correlate with itch severity in psoriasis and contributes to spinal itch signalling via astrocyte-derived release^44^. Here, intradermal injection of Lcn-2 into the male mouse cheek induces robust itch behaviour. Moreover, application of Lcn-2 to sensory neurons induced transient calcium influx. In Lcn-2 knockout mice, we observed a significant reduction in scratching behaviours in both MC903 and DNFB models, associated with downregulation of *Tbx20* in skin. However, we did not observe significant differences in scratching behaviour between the genotypes in female mice. It has been reported that Lcn-2 deficiency in female mice can influence estradiol biosynthesis and oestrogen receptor signalling, which may in turn affect brain function and behaviour—such as anxiety, stress responses, and cognition^59^. Although this is an interesting and relevant topic, it falls outside the scope of our current study.

Similar to keratinocyte-specific TBX20 knockout, global Lcn-2 deficiency led to a marked reduction of CD11b⁺ and CD68⁺ cell infiltration in MC903-treated skin, but not in those treated with DNFB. In Lcn-2 knockout, basophil infiltration was significantly reduced in the DNFB model, while only a downward trend was observed in MC903-treated skin. These findings suggest a context-dependent role for LCN-2, potentially reflecting the distinct inflammatory profiles—T_H_2-driven in MC903 versus T_H_1/T_H_17-dominant in DNFB. Notably, this mechanism appears distinct from astrocyte-derived LCN-2-mediated chronic itch in diphenylcyclopropenone-induced ACD, which operates through a STAT3-dependent transcriptional pathway^60^. Further investigation is needed to elucidate the molecular basis of these context-specific functions.

Although TBX20 is expressed in sensory neurons, our study revealed that a proportion of the total neurons that express NPR1 also express TBX20. The function of TBX20 in sensory neurons was elucidated in sensory neuron-selective TBX20 knockout mice using a cheek model with MC903 treatment, as this area is innervated by TG neurons, not dorsal root ganglionic neurons^61^. Knockout of TBX20 in Avil^+^ sensory neurons eliminated TBX20 along with *Npr1* and *Nppb* in these neurons, leading to attenuation of the MC903-induced itch response on the cheek. This was accompanied by downregulation of transcription levels of *IL13ra1*, *Il13ra2*, *IL31ra*, and *Tlr1* in the ipsilateral TG. IL-31RA expression is known to be upregulated in sensory neurons associated with chronic itch^62^ and AD-related murine models^63^, and its downregulation indicates a reduced itch response to IL-31.

In the MC903-induced cheek skin of TBX20^fl/fl^; Avil-Cre mice, infiltrations of Mcpt8^+^, CD11B^+^, and CD68^+^ cells were reduced, while expression of skin barrier proteins LOR and FLG2 was elevated compared to control TBX20^fl/fl^ mice. The reduction in Mcpt8⁺ cells following TBX20 knockout in keratinocytes and sensory neurons suggests that TBX20 regulates basophil recruitment to the skin. Given that basophils amplify T_H_2-driven inflammation and itch through T_H_2 promotion and release of IL-4, IL-13, and the pruritogen LTC₄ (Leukotriene C4), these findings link TBX20–NPR1 signalling to itch^47^. NPR1 expression in secondary sensory neurons plays a critical role in conveying pruritogen-elicited BNP signalling^7^, though it is also detected in primary sensory neurons^64^. While upregulation of BNP-NPR1 signalling in primary neurons has been implicated in pain inhibition^65^ and in neuropathic chronic itch^66^, its autocrine role in itch propagation remains unresolved. The precise function of NPR1 in primary sensory neurons is still unclear, despite intradermal injection of BNP inducing scratching in mice^11^. Notably, Avil, a somatosensory neuronal marker, is expressed in both primary and secondary sensory ganglia^67^, raising the possibility that TBX20 deletion in Avil^+^ primary neurons may influence spinal NPR1-mediated itch response to MC903. This warrants further investigation in future studies.

At the translational level, we proved that restoring TBX20 expression in the skin of TBX20^fl/fl^; K14-Cre mice via local AAV-mediated TBX20 gene delivery increased scratching behaviour induced by MC903 and was associated with elevated NPR1 expression, confirming the specificity of TBX20 in regulating the BNP-NPR1 pathway. Conversely, in WT mice, intraperitoneal injection of NRG1, a cardioactive growth factor released from endothelial cells^22^, suppressed TBX20 expression, alleviated MC903-induced itch, and reduced CD68^+^ and CD11b^+^ cell infiltration. Supporting this, in vitro studies show that NRG1 downregulated IL-13-induced TBX20 transcription and decreased NPR1 expression. Elevated levels of NRG1 have been reported under AD-modelled conditions and are known to enhance filaggrin expression, further supporting its anti-inflammatory role in AD pathogenesis^68^. Collectively, these findings highlight TBX20 as a key regulator of itch and suggest that targeting TBX20 with NRG1 may offer a novel therapeutic approach for chronic itch and skin barrier dysfunction.

## Methods

### Research ethics approval

All animal procedures were performed in accordance with the Guidelines for Care and Use of Laboratory Animals of Henan University and approved by the Animal Ethics Committee of Henan University. The human skin tissues were purchased from Tissue Solutions (Glasgow, Scotland); thus, obtaining additional Institutional Review Board approval and written informed consent was not necessary, as ethical approval and written informed consent were obtained from Tissue Solutions for the sample procurement. No human participant recruitment or compensation was performed by the authors.

### Mouse models

All animal experiments were conducted in accordance with the ARRIVE guidelines and reported in compliance with their recommendations. Animals were housed under a standard 12 hours light/dark cycle with food and autoclaved water provided ad libitum. Ambient temperature was maintained at 20–22 °C, and relative humidity at 40–60%. A standard autoclaved rodent chow diet (SPF-F01-002; SPF (Beijing) Biotechnology Co., Ltd.) was provided throughout the study. The diet contained approximately 18% crude protein, 5% crude fat, and 5% crude fibre. Six- to eight-week-old mice (body weight 16–18 g) were used for behavioural assessments. Mice were weighed daily to monitor weight loss and overall welfare. Terminal anaesthesia was induced by using inhalable anaesthetic and cervical dislocation, and death was subsequently confirmed by thoracotomy.

C57BL/6 breeder mice were obtained from SPF (Beijing) Biotechnology Co. Ltd., which we bred in-house. To generate cell-specific TBX20 conditional knockout mice (cKO), we crossed K14-Cre transgenic mice (genetic background C57BL/6N, Cyagen) or Avil-Cre-transgenic mice (genetic background C57BL/6J, Shanghai Model Organisms Center, Inc) with “floxed” TBX20 (TBX20^fl/fl^) mice (genetic background C57BL/6J, Cyagen). Progeny were genotyped by PCR as described below. cKO TBX20^fl/fl^; K14-Cre and TBX20^fl/fl^; Avil-Cre were specifically deficient in keratinocytes and sensory neurons, respectively. In both genotypes, exons 2- 3 of TBX20 are recombinantly excluded, resulting in a frameshift of the gene and loss of function of the mouse TBX20 gene. The size of the effective cKO region: ~3100 bp. TBX20^fl/fl^ littermate mice were used as controls for comparison in behaviour testing. For all the behaviour testing and post-analysis, only female TBX20 transgenic mice were used. The whole-body LCN-2 knockout mouse strain C57BL/6JCya-*Lcn2*^*em1*^/Cya (S-KO-02865) was purchased from Cyagen. Exons 1–6 were excised from the LCN-2 gene in the LCN KO mice. The size of the effective KO region: ~3.2 kb. Male LCN-2 knockout transgenic mice and WT mice were used for behaviour testing.

### Identification and genotyping of mouse

To ensure that mice of the correct genotype were used in cKO studies, progeny at 3 weeks old were genotyped by PCR. Tail tip (~3 mm) was incubated at 95^o^C for 30 mins in 100 µL mouse tail lysis buffer (10 mM NaOH and 0.1 mM EDTA). Tail segments were allowed to cool to room temperature before being centrifuged at 1788.8 g for 5 minutes and supernatant collected for PCR analysis. The thermocycler conditions were as follows: one initial 95 °C step for 3 mins; followed by 35 cycles of denaturation (95 °C for 30 seconds), annealing (60 °C for 30 seconds), and extension (72 °C for 30 seconds) with a final extension at 72 °C for 5 mins at the end. PCR products were then analysed by agarose gel electrophoresis. Genotyping of TBX20^fl/fl^; K14-Cre and TBX20^fl/fl^; Avil-Cre mice was performed by PCR using primer pairs specific for the *Tbx20* floxed allele and the respective *Cre* transgene. For detection of the *Tbx20* floxed allele, primers *Tbx20*-F (5′-CTTCTCTATTTCTGTGGTGCTCTG-3′) and *Tbx20*-R (5′-TACCAACATCCTGAAACACTTTTGG-3′) were used with an annealing temperature of 60 °C, generating a 223 bp band for the floxed allele and a 149 bp band for the wild-type allele, while heterozygous mice displayed both bands.

For identification of the *K14-Cre* transgene, primers *K14*-F (5′-CGATGGGAAAGTGTAGCCTGCA-3′) and *K14*-R (5′-TCCAGGTATGCTCAGAAAACGCC-3′) were used at an annealing temperature of 60 °C, producing a 450 bp *Cre*-specific amplicon.

For *Avil-Cre* genotyping, primers *Avil*-F (5′-GTGGTGGTGGCATGGTGATA-3′) and *Avil*-R (5′-GCACACAGACAGGAGCATCT-3′) were used under the same PCR conditions, generating a 327 bp Cre-specific amplicon. Combined analysis of these PCR products allowed reliable identification of TBX20^fl/fl^; K14-Cre and TBX20^fl/fl^; Avil-Cre mice.

For *Lcn-2* knockout genotyping, two primer sets were used. Primer set 1: F1 (5′-CAACTCAGAACTTGATCCCTGCC-3′) and R1 (5′-TTTCCCTAAGTCCCGTTCAATCC-3′), producing a 597 bp product for the knockout allele. Primer set 2: F2 (5′-ATGTACAGCACCATCTATGAGCTA-3′) and R2 (5′-TTTCCCTAAGTCCCGTTCAATCC-3′), producing a 729 bp product. The wild-type allele produces a 1287 bp band. PCR was performed with an annealing temperature of 60 °C. Genotypes were identified as follows: homozygous KO mice showed a single band at 597 bp; heterozygous mice showed three bands at 597 bp, 729 bp, and 1287 bp; wild-type mice showed two bands at 729 bp and 1287 bp. Only homozygous KO mice were used for the experiment.

### Animal models and itch behaviour scoring

#### BNP cheek injection

One day before BNP injection the fur from both sides of the cheeks of 7-week-old female TBX20^fl/fl^; K14-Cre mice and TBX20^fl/fl^ mice was shaved off. On the following day, 10 μg/10 μL of recombinant BNP (011-23, Phoenix Biotech, Kanpur, India) in physiological saline solution supplemented with 0.1% w/v BSA was injected intradermally (*i.d*) into the left cheek, with the vehicle (physiological saline solution with 0.1% w/v BSA) injected into the right cheek, as previously established^11^. Behaviour was recorded for 30 mins to capture the acute responses. Mice were then euthanised five hours after the recording in a biosafety cabinet. Their cheek tissues were collected using surgical scissors, immediately frozen in liquid nitrogen, and stored at -80 °C in RNase-free 2 mL tubes for future use.

#### AD mouse model induced by MC903 in *Tbx20* transgenic mice

12 female mice per group of TBX20^fl/fl^;K14-Cre and TBX20^fl/fl^, or 7 female mice per group of TBX20^fl/fl^;Avil-Cre and TBX20^fl/fl^ were acclimated in a behavioural observation chamber (10 cm×10 cm) for 60 mins on day 0. On day 1, each mouse was applied with MC903 (4 nmol/20 μL) to the dorsal surface of the left ear, while ethanol (vehicle) was applied to the dorsal surface of the right ear of TBX20^fl/fl^; K14-Cre mice and TBX20^fl/fl^ for 8 consecutive days to fully induce the chronic dermatitis phenotype, as previously established^8^. However, in the case of TBX20^fl/fl^; Avil-Cre and TBX20^fl/fl^ mice, MC903 (4 nmol/20 μL) was applied to the left check with vehicle (ethanol) applied to the right cheek for 7 consecutive days. Immediately after, scratching behaviours were recorded for 60 minutes to capture the peak responses. Five hours after the last application of MC903, the mice were euthanised. The left ear/cheek was harvested and divided into two parts: one part was used for sectioning, and the other for RNA extraction, according to our established protocol^47^. Additionally, the left trigeminal ganglion (TG) from TBX20^fl/fl^; Avil-Cre mice was used for sectioning, while the right TG was used for genotyping confirmation. Some tissue was fixed by immersion in a 4% paraformaldehyde solution, while the portion intended for RNA extraction was immediately frozen in liquid nitrogen.

#### Allergic Contact Dermatitis (ACD) induced by 2,4-dinitrofluorobenzene (DNFB) in TBX20 transgenic mice

At day 0, the fur of seven-week-old female TBX20^fl/fl^; K14-Cre and TBX20^fl/fl^ mice (6 mice per group) was shaved on a 2 cm × 2 cm area of their abdomens, and 25 μL of 0.5% DNFB (dissolved in a 4:1 mixture of acetone: oil) was applied to prime the immune response. After a four-day interval, on day 5, 10 μL of 0.3% DNFB (dissolved in a 4:1 mixture of acetone: oil) was applied to the dorsal surface of the left ear to initiate dermatitis and itch behaviour, while 10 μL of vehicle (4:1 acetone:oil) was applied to the dorsal surface of the right ear as a control, as previously established^69^. This procedure was repeated daily from day 6 to day 12. The scratching behaviour of the mice was recorded for 60 minutes to capture the peak responses. On day 12, five hours after the final recording, the mice were euthanised in a biosafety cabinet. The ear tissue samples were dissected, with one half placed in 4% paraformaldehyde and the other half immediately frozen in liquid nitrogen.

#### AAV-TBX20 injection in MC903-induced cheek model

To restore TBX20 function in cKO mice, Adeno-associated virus (AAV2/9) carrying TBX20 for overexpression (pAAV-CMV-mCherry-P2A-Tbx20-3xFLAG-WPRE) and an equal amount of control virus (pAAV-CMV-mCherry-P2A-3xFLAG-WPRE), both from OBiO, Shanghai, were injected into the cheeks of TBX20^fl/fl^; K14-Cre cKO mice (7 mice/group) every other day, for a total of four injections. The total virus dose per injection for experimental and control groups was 2.5 × 10^11^ particles. Three days after the last injection, 20 μL of 2 nmol MC903 was applied to the cheeks of TBX20^fl/fl^; K14-Cre mice. The mice were then placed in a behavioural observation chamber. MC903 application and scratching behaviour observation were repeated for 13 consecutive days. Five hours after the final observation on the last day, the mice were euthanised, and cheek skin samples were collected, frozen in liquid nitrogen, and stored at −80 °C.

#### Neuregulin 1 (NRG1) administration in MC903-induced ear model

Female C57BL/6J mice (6–7 weeks old) were acclimated in the animal facility for five days. From Day 1-4, 20 μl of 4 nmol MC903 was applied to the left ear of each mouse, and scratching behaviour was immediately recorded for one hour. On Day 5, the mice were randomly divided into two groups. The experimental group received an intraperitoneal (*i.p*) injection of NRG1 (9875-NR, R&D; 200 μg/kg, dissolved in sterile PBS), while the control group received an *i.p*. injection of an equal volume of PBS. One hour after the injection, 20 μL of 4 nmol MC903 was applied to the left ear of each mouse and scratching behaviour was recorded for one hour. This procedure was repeated on Days 6 & 7.

#### LCN-2 injection

Sixteen C57BL/6J (WT) male mice were randomly divided into two groups: the model group and the control group. Each mouse in the experimental group received an injection of a 1 μg/20 μL recombinant LCN-2 (1857-LC-050, R&D) into the right cheek. The control group was injected with the vehicle solution of 25 nmol/L MES (Sigma), 150 nmol/L NaCl, pH 6.5. The scratching behaviour of the mice was recorded for one hour to capture peak acute itch response.

#### MC903 and DNFB modelling in LCN-2 knockout mice

For the MC903 model, 7 male LCN-2 KO mice and 7 wild-type littermates received 4 nmol/20 μL MC903 on the left ear daily for 7 days. Scratching behaviour was recorded immediately, and body weight was measured. For the DNFB model, 6 male LCN-2 KO mice and 6 wild-type littermates were sensitized with 25 μL of 0.5% DNFB on a shaved abdominal area. After 4 days, 10 μL of 0.3% DNFB was applied daily to the left ear and vehicle to the right ear for 6 days. Scratching behaviour was recorded for 1 hour after each application, and tissues were harvested five hours after the final observation on the last day. In some experiments, behaviour was recorded one hour prior to the application of MC903 and the challenge with DNFB.

### Scratching behaviour scoring

To prevent bias, the scratching behaviour was scored by individuals who were blinded to the experimental conditions and unfamiliar with the experimental aims. These individuals were trained and assessed for their ability to discern scratching behaviour and pain responses to reduce variability in scoring between assessors as much as possible. For all the itch behaviour scoring, at least 1 hour before the itch inducer application, the mice were placed individually in a transparent multiple-configuration animal enclosure (10 cm×10 cm) (Ugo Basile) to acclimatise them to the environment. After that, mice were video recorded using EthoVision XT behaviour recording equipment (Noldus). No personnel were present during the recording. The mouse’s hind limb scratching at the injection or pruritogen application site indicated a sign of the animal’s scratching behaviour. Lifting the hind limb and then putting it down or bringing it to the mouth to clean the foot counts were counted as one scratching bout^47^.

### RNA-sequencing

RNA sequencing (RNA-seq) was conducted by BGI (Beijing Genomics Institute). Total RNA was extracted from the cells using TRIzol (Invitrogen, Carlsbad, CA, USA). The mRNA was purified using oligo(dT)-attached magnetic beads. Purified mRNA was fragmented before cDNA synthesis using random hexamer-primed reverse transcription. cDNA fragments underwent end repair by adding A-Tailing Mix and RNA Index Adapters. PCR amplification was performed on the obtained cDNA fragments, and the products were purified using Ampure XP Beads and dissolved in EB solution. Quality control (QC) was conducted using the Agilent Technologies 2100 bioanalyser to validate the resulting cDNA fragments. The double-stranded PCR products were denatured and circularised using the splint oligo sequence to generate the final library. The single-stranded circular DNA was then prepared as the final library. Phi29 amplification was performed to create DNA nanoballs (DNBs), each containing more than 300 copies of one molecule. The DNBs were loaded onto a patterned nanoarray, and single-end 50-base reads were generated using a DNBseq platform (BGI-Shenzhen, China). The initial sequencing data obtained are referred to as raw reads or raw data, which underwent QC to determine their suitability for subsequent analysis. The software SOAPnuke, developed independently by BGI, was employed to filter the sequencing data (Parameters: -l 15 -q 0.2 -n 0.05). After QC, the filtered clean reads were aligned to the reference sequence. Hierarchical indexing for spliced alignment of transcripts (HASAT) is the software for mapping RNA-seq reads. After the alignment, the statistics of the mapping rate and the distribution of reads on the reference sequence are used to determine whether the alignment result passes the second QC of alignment. If it passes, we perform quantitative gene analysis and other analyses based on gene expression (principal component, correlation, differential gene screening, etc.). The data analysis platform under BGI (Dr. Tom) was exploited for bioinformatically processing analysis of Fragments Per Kilobase per Million mapped fragments (FPKM), Differentially Expressed Gene (DEG), and heatmap.

### Real-time quantitative reverse transcription PCR (RT-qPCR)

Total RNA was extracted from murine skin or cells using the Nucleospin RNA Midi kit (74104, QIAGEN) following the manufacturer’s instructions. The concentration of RNA was determined using a Nanodrop 2000 spectrophotometer (Thermo Fisher Scientific). cDNA was prepared using a high-capacity cDNA reverse transcription kit (4368814, Thermo Fisher Scientific), where 1 μg of total RNA was reverse-transcribed into cDNA. For RT-qPCR analysis, we used the SYBR Green PCR Master Mix (4309155, Thermo Fisher Scientific) and primers (Supplementary Table 1).

### Immunohistochemistry/Immunofluorescence Staining

After euthanasia, mouse tissue samples were fixed by immersion in 4% w/v paraformaldehyde. After fixation, the tissues underwent a gradient dehydration process using ethanol solutions of increasing concentration (70%, 80%, 90%, 95%, and 100%), each for 40 mins. Subsequently, tissue clarification was performed by sequentially immersing the tissues in three paraffin baths, each lasting 1 hour. The well-soaked paraffin tissue blocks were placed flat at the bottom, and liquid paraffin was poured into the mould boxes. After cooling, the paraffin blocks were removed. Pre-chilled paraffin blocks were affixed to a paraffin microtome, ensuring that the cut surface of the paraffin block was parallel to the blade, and sections of uniform thickness (4 μm) were cut. The resulting glass slides were stored at 4 °C. Paraffin sections of mouse and human skin as well as sensory ganglia were deparaffinised, rehydrated, and permeabilised in PBS containing 0.2% v/v Triton X-100 and then incubated for 1 hour in 5% w/v normal donkey serum in PBS (blocking solution) at room temperature. After blocking, the specimens were incubated overnight at 4 °C with a primary antibody (Supplementary Table 2). Following incubation, the specimens were washed with PBS and treated with fluorescence-labelled secondary antibodies for 1 hour at room temperature. After washing again with PBS, the specimens were mounted using ProLong Anti-fade Reagent containing DAPI (4’,6-diamidino-2-phenylindole) (Thermo Fisher Scientific). Images were captured using an Olympus IX73 inverted microscope and CellSens Dimension Imaging software or a Leica DMi8 confocal microscope using LASX software.

### Hematoxylin and Eosin (H&E) staining

H&E staining was performed as before^13^. For staining, the sections were soaked in hematoxylin for 10 mins, and excess hematoxylin stain was subsequently rinsed off with PBS. The sections were treated with 0.5% hydrochloric acid in ethanol (acid alcohol) for 30 seconds to differentiate the stain, and the surplus acid alcohol solution was removed with deionized water. The glass slides were placed in water for 5–10 mins for bluing. Subsequently, they were stained with eosin for 5 minutes and rinsed clean with deionised water. The glass slides underwent two cycles of dehydration in absolute ethanol for 2 mins per cycle. Finally, the glass slides were cleared in xylene to complete the staining process.

### Dual-Luciferase Reporter Assay

Binding sites of the hTBX20 and hNPR1 genes were predicted using the JASPAR database (http://jaspar.binf.ku.dk/) based on the hTBX20 matrix profile MA0689.1. Wild-type (Wt) and mutant (Mt) hNPR1 promoter sequences were synthesized by Hanbio Biotechnology, with the Mt construct containing simultaneous mutations at all four predicted TBX20 binding sites.

To assess promoter activity and transcription factor–promoter interactions, a dual-luciferase reporter system (Hanbio Biotechnology) was used. Wt and Mt hNPR1 promoter fragments were cloned upstream of the firefly luciferase (Fluc) gene in the linearized pGL3-Basic vector. The TBX20 coding sequence (NM001077653.2) was synthesized and cloned into the pcDNA3.1 plasmid for overexpression. The pRL-TK plasmid, expressing Renilla luciferase (Rluc), served as an internal control.

For luciferase assays, HaCaT and HEK293 (iCell-h237, iCell Bioscience Inc) at 70–80% confluence in 96-well plates were co-transfected with a reporter plasmid (wild-type or mutant NPR1 promoter cloned into the pGL3-Basic vector), a transcription factor expression plasmid (pcDNA3.1-TBX20), and the pRL-TK control plasmid using TransPi^TM^ Mate Reagent (1000M-0.1, PolyGene Bio) and buffer for TransPiTM Mixing (T567422, PolyGene Bio). A total of 1 µg plasmid DNA per well was used (pRL-TK: 0.2 µg; pGL3 construct: 0.4 µg; pcDNA3.1: 0.4 µg), and cells were incubated for 48 hours. Cells were then stimulated post-infection with BNP (1 μM), IL-13 (100 ng/ml, 213-ILB-025, R&D), or vehicle (PBS) for 24 hours.

Luciferase activity was measured using the Dual-Luciferase® Reporter Gene Assay Kit (Beyotime; RG027). Promoter activity was calculated as the ratio of firefly to Renilla luciferase activity (Fluc/Rluc).

### RNAscope multiplex fluorescent in situ hybridization

RNAscope was performed in three stages: pretreatment, hybridization/amplification, and detection. For FFPE samples, sections were baked at 60 °C for 1 hour, deparaffinized in xylene, rehydrated through graded ethanol, and air-dried. Endogenous peroxidase activity was blocked with hydrogen peroxide for 10 mins at room temperature, followed by washing. Target retrieval was performed in preheated 1× retrieval buffer at 98–102 °C for 15–30 mins. Slides were then washed, dehydrated in 100% ethanol, dried, and circled with a hydrophobic barrier. Sections were treated with RNAscope Protease Plus at 40 °C for 30 mins and washed. For hybridization, probes (target, positive, and negative controls) were applied to fully cover the tissue and incubated at 40 °C for 2 hours, followed by washing. Signal amplification steps (Amp 1–3) were performed sequentially at 40 °C with intermediate washes. For fluorescent detection, HRP-C1 and HRP-C2 reagents were applied sequentially, each followed by TSA fluorophore development (Vivid 520 for C1 and Vivid 690 for C2), HRP blocking, and washing steps. Nuclei were counterstained with DAPI, and slides were mounted with antifade medium prior to imaging using an Akoya scanner (Akoya Biosciences PhenoImager™ HT) using Phenochart 1.0.12 software. For human skin and murine TG, the reagents are listed in Supplementary Table 3.

The procedures for human dorsal root ganglion (DRG) sections (HP-240, Amsbio) were performed using RNAscope™ Multiplex Fluorescent Reagent Kit v2 (323100, ACD) in the same manner, with the probes listed in Supplementary Table 4, and images taken by Leica DMi8 confocal microscope using LASX software.

### Isolation and Cultivation of Primary Mouse Keratinocytes (mKCs) and Trigeminal Ganglionic Neurons (TGNs)

TBX20^fl/fl^; K14-Cre and TBX20^fl/fl^ were euthanized at 6-8 weeks of age, before their tail segments were dissected. An incision along the tail skin from the base to the tip was made using a sharp blade. Subsequently, the tissue was removed from the tailbone using a pair of tweezers, and the mouse tail was cut into several segments of approximately 3 cm in length. The tail skin was washed twice with calcium- and magnesium-free PBS containing 2% penicillin/streptomycin (v/v). Then, the tail skin was incubated at 4 °C overnight (~12 h) with digestion buffer (4 mg/ml Dispase II) in a 2 ml centrifuge tube on a horizontal shaker. The fully digested mouse skin was washed with PBS containing 2% antibiotics. The dermis was separated from the epidermis using a pair of tweezers. The epidermis was placed in a new Petri dish containing 0.05% trypsin with the basal layer facing down. This digestion was incubated at 37 °C for 6–8 minutes, with gentle shaking twice. An equal volume of DMEM supplemented with 10% v/v serum was added to stop the digestion. Keratinocytes were released by gently rubbing the epidermis with tweezers. A pipette was used to triturate the cell suspension, which was then passed through a 70 μm cell strainer. The flow-through was transferred to a centrifuge tube and spun at 54.5 g for 5 mins. The cell pellet was suspended in complete keratinocyte medium (KGM Gold Keratinocyte Growth Medium BulletKit, 00192060, Lonza), counted using a hemacytometer, and 1 × 10^5^ cells per well were seeded onto the mouse tail collagen pre-coated 24 plate. This plate was prepared by dispensing 200 μl of mouse tail collagen (50 μg/mL, abs47014921, ABSIN) per well 24 hours before cell seeding and was stored at 4 °C. Cells were cultured at 37 °C in 5% CO₂, and the medium was replaced every two days until approximately 70% confluence was reached. Cells were then washed twice with pre-warmed calcium- and magnesium-free PBS and incubated in keratinocyte basal medium for 6 hours, followed by stimulation with BNP (1 μM) for 24 hours. After stimulation, the culture supernatant was collected, centrifuged at 4 °C, 111.8 *g* for 5 mins, and the clarified supernatant was used for measurement of the released cytokines by the Proteome Profiler Murine XL Cytokine Array Kit (ARY028, Bio-Techne) as per the manufacturer’s instructions.

For calcium imaging of sensory neurons, mouse TG were isolated from postnatal C57BL/6 mice or TBX20^fl/fl^ mice, dissociated by collagenase I, and cultured as previously described^8,13^. After four days in culture, the cells were used for the experiment.

### Chromatin Immunoprecipitation (ChIP) and qPCR assay of BNP-stimulated keratinocytes

The human keratinocyte cell line (HaCaT) was cultured in DMEM supplemented with 1% penicillin-streptomycin solution and 10% FBS and grown to 70% confluency. The cells were equilibrated with preheated DMEM for 6 hours. After equilibration, the medium was discarded, and the cells were stimulated with fresh serum-free media containing BNP (1 μM; Phoenix Biotech) or vehicle control (PBS) for 24 hours. Following stimulation, the cell bodies were harvested and processed using the EpiQuik ChIP Kit (Catalogue number P-2002-3, Epigentek) according to the manufacturer’s protocol. The purified DNA obtained after cross-link reversal was subsequently analysed by SYBR green qPCR.

### Treatment of HaCaT cells with A71915 and BNP

HaCaT cells (BioCat GmbH) were cultured in vitro to 70% confluency and equilibrated in preheated DMEM for 6 hours prior to treatment with BNP (1 μM) or vehicle, in the presence or absence of A71915 (6715, Bio-Techne), for 24 hours in serum-free medium. TBX20 transcription was analysed using RT-qPCR.

### Treatment of NHEK with IL-13 and NRG1

Primary human adult epidermal keratinocytes (NHEK, 00192627, Lonza, Basel, Switzerland) were seeded into 24-well plates and cultured in KBM-Gold medium supplemented with KGM Gold SingleQuot Keratinocyte Supplement (00192060, Lonza). Cells were incubated in the medium for 3 days before any experiments were conducted. NHEKs were then stimulated for 24 hours with either the medium alone, IL-13 (100 ng/ml, 213-ILB-025, R&D) ± Neuregulin-1 (NRG1, 2 nmol/L, CF 5898-NR-050, R&D Systems), or medium + Neuregulin-1. Following stimulation, cell cultures were lysed using TRIzol and stored at −80 °C until analysis by RT-qPCR.

### Intracellular Ca^2+^ measurement in mouse TGNs

Murine TGNs isolated from WT mice were cultured in 8-well IBIDI chambers and loaded with 3 μM Fluo-4 AM (F14201, Thermo Fisher) for 30 mins at 37 °C. Cells were then washed and allowed to equilibrate to room temperature before being stimulated with 1 μM BNP (011-23, Phoenix Biotech, Kanpur, India) or 1 μg/ml Lcn-2 (1857-LC-050, R&D Systems) or their vehicle (PBS), and time-lapse images were acquired using an ImageXpress Micro 4 Automated Cell Imaging System (Molecular Devices) with MetaXpress 6 software. Intracellular calcium responses were quantified as F/F₀, where F represents the fluorescence intensity at a given time point, and F₀ represents the baseline fluorescence.

To assess the effect of keratinocyte-specific TBX20 depletion on sensory neuron activation, murine keratinocytes (mKCs) from TBX20^fl/fl^; K14-Cre and TBX20^fl/fl^ mice were stimulated with 1 μM BNP for 24 hours. Conditioned media were collected and applied to Fluo-4 AM–preloaded TGNs isolated from TBX20^fl/fl^ mice. Intracellular calcium responses, quantified as F/F₀, were analysed, and the number of responsive cells was determined relative to the total number of neuronal cells.

### Short hairpin RNA-mediated knockdown of TBX20 in NHEK cells

NHEKs were cultured in 12-well plates for 24 hours in KBM-Gold medium supplemented with KGM-Gold SingleQuot KC supplement (00192060, Lonza), prior to treatment with either non-targeted scrambled lentiviral particles or PLKO.1-puro lentiviral particles carrying short hairpin RNA (shRNA) targeting TBX20 (OBiO Technology Corp Ltd) in hydrocortisone-free Lonza KGM gold keratinocyte growth medium (400 transducing units per well). Validated shRNA sequences targeting TBX20 for lentiviral transduction particles: TBX20 shRNA, CCGGGCGACGGAGAACACAATCAAACTCGAGTTTGATTGTGTTCTCCGTCGCTTTTT. After 2 days in culture, the cells were further cultured in medium containing 1 μg/mL of puromycin (Sigma) for 3 days. Cells were then treated with BNP (1 μM) for 24 hours, after which BNP-induced release was measured using Human XL cytokine antibody arrays (ARY022B, Bio-Techne). Cell bodies were lysed in LDS sample buffer for Western blotting analysis to confirm knockdown. TBX20 was detected using an affinity-isolated anti-TBX20 antibody produced in rabbit (AV33177, Sigma). Syntaxin 4 was detected as an internal control protein using a monoclonal antibody (H00006810-M04, Novusbio).

### Data analysis

Data are presented as mean ± SEM and displayed as dot plots or bar graphs. All data are derived from at least 3 independent experiments. Comparisons between two groups were performed using a two-tailed Student’s *t*-test with Welch’s correction. For selected datasets, as specified in the figure legends, a 2-way ANOVA followed by multiple comparisons testing was applied. The criterion for statistical significance is ^ns^*P* > 0.05, *P < 0.05; **P < 0.01; ***P < 0.001; ****P < 0.001 (Prism 10.1.0, GraphPad Software, La Jolla, CA, USA).

### Reporting summary

Further information on research design is available in the Nature Portfolio Reporting Summary linked to this article.

## Supplementary information

Supplementary Information

Peer Review file

Reporting Summary

## Data Availability

All data described in this study are included in the manuscript and the accompanying Supporting Information. All RNA-seq datasets associated with this article have been deposited in the Gene Expression Omnibus (GEO) under accession code GSE281758. All relevant raw data from figures generated in this study have been provided in the Source Data file. Source Data are provided with this paper^70^.

## Code availability

This study did not involve the development of custom code. Accordingly, no custom code was generated or deposited in a public repository. Code used for the identification of TBX20 consensus sequences in ChIP peak calling related to Fig. 3a is provided in the Source Data file. These analyses were performed using established, publicly available software and packages.
