# Supplementary material for: Misregulation of T-box transcription factors drives BNP–NPR1 signalling underlying chronic itch
Source: Nat Commun. 2026 Jul 17;17:8754. doi: 10.1038/s41467-026-75726-x (PMC13493844; doi:10.1038/s41467-026-75726-x)
Supplement: Supplementary file 1 [file 41467_2026_75726_MOESM1_ESM.pdf]

Supplementary Fig. 1. Representative images of RNAscope for positive and negative control probes on human skin. Positive control probes (Polr2a, Ppib, and UBC) show robust labelling, whereas the negative control probe (bacterial DapB) shows no signal. Scale bar = 50 μm.

Supplementary Fig. 1

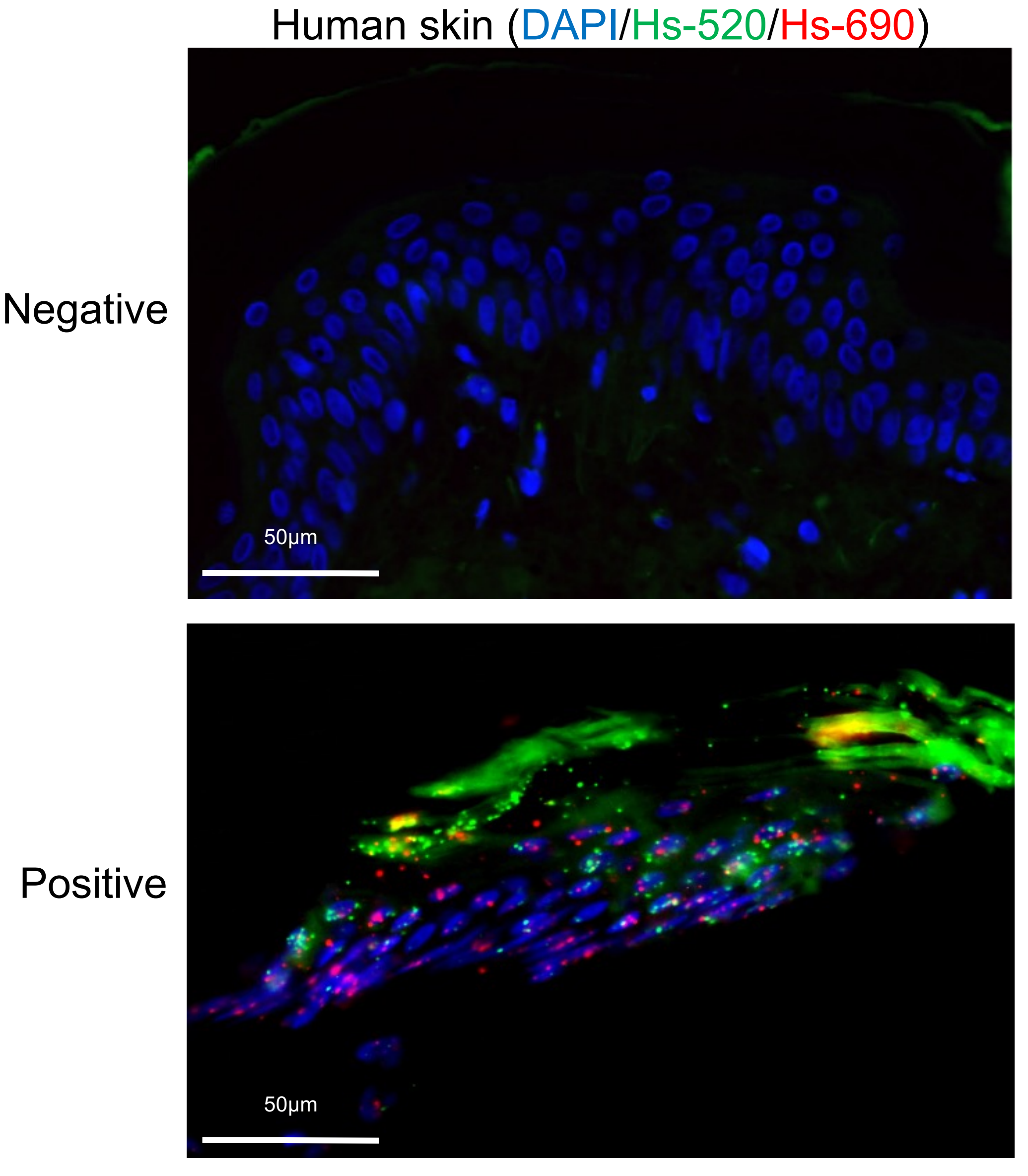

Supplementary Fig. 2. Schematic map of the hNPR1 promoter cloned into the pGL3-Basic vector, and sequences of the wild-type (Wt) and mutant (Mt) hNPR1 promoter regions. Following insertion, sequencing confirmed that the cloned Mt *hNPR1* promoter contains four simultaneous mutations at all predicted binding sites (highlighted). Portions of the pGL3-Basic vector backbone are also shown (highlighted).

Supplementary Fig. 2

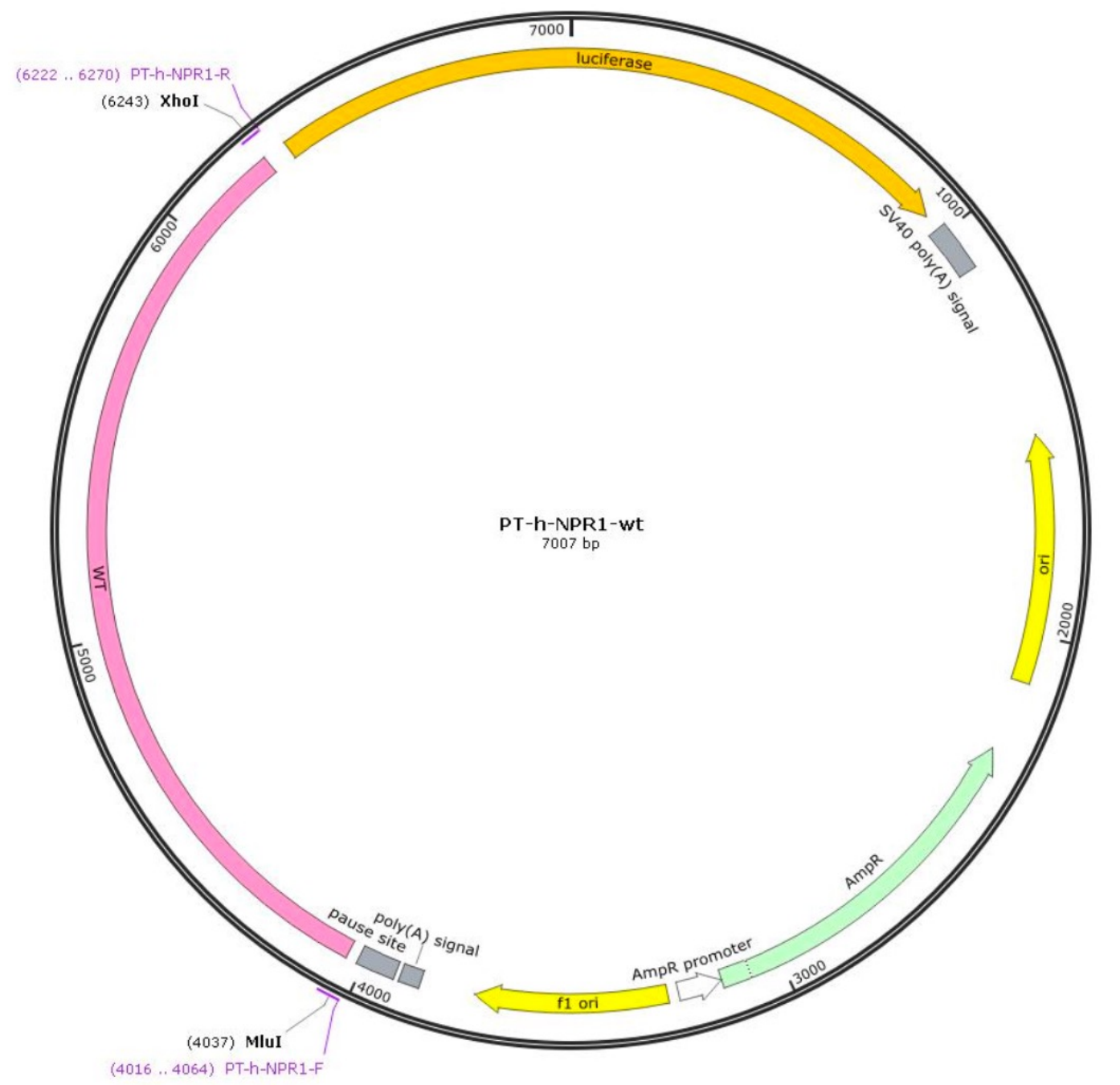

Wt hNPR1 promoter sequence (Select the region from 2000 bp upstream of the TSS to 200 bp downstream (NC_000001:153676688..153678887) :

```
hNPR1-Wt.txt    ................................................................................................      0
h-NPR1-Mt.txt   TCATCTTTATGCCCTCGGCACCTAGTGGGTGCTTAATGCACCGAAGGTTCTCTGGAAATGTTTGCTGACCTGAAGTTTGGTGATCTTATGTCCTGC     96
Consensus

hNPR1-Wt.txt    .................................................................................GCAGTCTGCACCGGG     15
h-NPR1-Mt.txt   TTAGGCGCAGCGTTGGAGAGGTTGGCGCTGCAATGCCGTCAATAACCCCCGATGACCATTAGAGGGCACACTGATTCCACAGCAGTCTGCACCGGG    192
Consensus                                                                                        gcagtctgcaccggg

hNPR1-Wt.txt    GGCCCAGCTGTCAAGTGCTCCAAAACCCACTCCCCAATTTACGCCTCCAGTGGTCATCTCCCACAGCCTTCATTTTAACAACCCTCAACACAAAAC    111
h-NPR1-Mt.txt   GGCCCAGCTGTCAAGTGCTCCAAAACCCACTCCCCAATTTACGCCTCCAGTGGTCATCTCCCACAGCCTTCATTTTAACAACCCTCAACACAAAAC    288
Consensus       ggcccagctgtcaagtgctccaaaacccactccccaatttacgcctccagtggtcatctcccacagccttcattttaacaaccctcaacacaaaac

hNPR1-Wt.txt    AGTCATTTTCTTGGGGCGTCCCTGAGGTGTGAACCTCCTCCCTCCCAGACACCCACCCACTCTCCCCATCTTCCTACAATTTGGGCGCTAAAGATT    207
h-NPR1-Mt.txt   AGTCATTTTCTTGGGGCGTCCCTCACGAGAGTAGCTCCTCCCTCCCAGACACCCACCCACTCTCCCCATCTTCCTACAATTTGGGCGCTAAAGATT    384
Consensus       agtcattttcttggggcgtccct a g g g a ctcctccctcccagacacccacccactctccccatcttcctacaatttgggcgctaaagatt

hNPR1-Wt.txt    TAATTCCTCTCTGACACCAGATCATTTTTTCACAGCTAATCTGCACAGTCTGGAAGGTGGCAGGGAGGGAAAGAGAGGGAGATCATAGAAGGAATG    303
h-NPR1-Mt.txt   TAATTCCTCTCTGACACCAGATCATTTATACTCTGGTTATCTGCACAGTCTGGAAGGTGGCAGGGAGGGAAAGAGAGGGAGATCATAGAAGGAATG    480
Consensus       taattcctctctgacaccagatcattt t c c g t atctgcacagtctggaaggtggcagggagggaaagagagggagatcatagaaggaatg

hNPR1-Wt.txt    GGTAACAAGCACTCTGGGCTCACCTTCAACTTTAACGGACTTTCCAGAAACCCGTCACCTTCCACCCCACACCCAAACACACACATCCACGCACTC    399
h-NPR1-Mt.txt   GGTAACAAGCACTCTGGGCTCACCTTCAACTTTAACGGACTTTCCAGAAACCCGTCACCTTCCACCCCACACCCAAACACACACATCCACGCACTC    576
Consensus       ggtaacaagcactctgggctcaccttcaactttaacggactttccagaaacccgtcaccttccaccccacacccaaacacacacatccacgcactc

hNPR1-Wt.txt    ACTCAGTTTCCTGCCAGGATCCCAAACCAGCACACCTTTCCCTCTTCCCCCGAGGAGACCAGGTAGGAGGCGAGGGAAAAGGTGGGGCGCAAGTGG    495
h-NPR1-Mt.txt   ACTCAGTTTCCTGCCAGGATCCCAAACGACCTCTCGTATCCCTCTTCCCCCGAGGAGACCAGGTAGGAGGCGAGGGAAAAGGTGGGGCGCAAGTGG    672
Consensus       actcagtttcctgccaggatcccaaac a c c c t tccctcttcccccgaggagaccaggtaggaggcgagggaaaaggtggggcgcaagtgg

hNPR1-Wt.txt    GCCCCGGTTGCTTCCACACACACCCTCCGTTCAGCCGTCCTTTCCATCCCGGCGAGGGCGCACCTTCAGAGGGTCCTGTCCTCCAAAGAGGTAGGC    591
h-NPR1-Mt.txt   GCCCCGGTTGCTTCCACACACACCCTCCGTTCAGCCGTCCTTTCCATCCCGGCGAGGGCGCACCTTCAGAGGGTCCTGTCCTCCAAAGAGGTAGGC    768
Consensus       gccccggttgcttccacacacaccctccgttcagccgtcctttccatcccggcgagggcgcaccttcagagggtcctgtcctccaaagaggtaggc

hNPR1-Wt.txt    GTGGGGCGGCCGAGACCGGGGAAGATGGTCCACGGGGAAGCGCGCGGGCTGGGCGGCGGGGAGGAAGGAGTTAAAGATCCTGGATTGGCTCTTCTG    687
h-NPR1-Mt.txt   GTGGGGCGGCCGAGACCGGGGAAGATGGTCCACGGGGAAGCGCGCGGGCTGGGCGGCGGGGAGGTACGTGATTATGATCCTGGATTGGCTCTTCTG    864
Consensus       gtggggcggccgagaccggggaagatggtccacggggaagcgcgcgggctgggcggcggggagg a g g t a gatcctggattggctcttctg

hNPR1-Wt.txt    TCACTGAGTCTGGGAGGGGAAGCGGCTGGGAGGGAGGGTTCGGAGCTTGGCTCGGGTCCTCCACGGTTCCCTCCGGATAGCCGGAGACTTGGGCCG    783
h-NPR1-Mt.txt   TCACTGAGTCTGGGAGGGGAAGCGGCTGGGAGGGAGGGTTCGGAGCTTGGCTCGGGTCCTCCACGGTTCCCTCCGGATAGCCGGAGACTTGGGCCG    960
Consensus       tcactgagtctgggaggggaagcggctgggagggagggttcggagcttggctcgggtcctccacggttccctccggatagccggagacttgggccg

hNPR1-Wt.txt    GCCGGACGCCCCTTCTGGCACACTCCCTGGGGCAGGCGCTCACGCACGCTACAAACACACACTCCTCTTTCCTCCCTCGCGCGCCCTCTCTCATCC    879
h-NPR1-Mt.txt   GCCGGACGCCCCTTCTGGCACACTCCCTGGGGCAGGCGCTCACGCACGCTACAAACACACACTCCTCTTTCCTCCCTCGCGCGCCCTCTCTCATCC   1056
Consensus       gccggacgccccttctggcacactccctggggcaggcgctcacgcacgctacaaacacacactcctctttcctccctcgcgcgccctctctcatcc

hNPR1-Wt.txt    TTCTTCACGAAGCGC...............                                                                      894
h-NPR1-Mt.txt   TTCTTCACGAAGCGCTCACTCGCACCCTTT                                                                     1086
Consensus       ttcttcacgaagcgc
```

Supplementary Fig. 3. Mutation of *TBX20* binding sites abolishes BNP-induced *NPR1* promoter activation in HEK293; NPR1 inhibitor A71915 attenuates BNP-induced *TBX20* transcription in human keratinocytes.

a. Fluc/Rluc reporter activity was measured in BNP-induced and IL-13-induced Wt-h*NPR1*- and Mt-h*NPR1*- HEK293 cells. Firefly luciferase (Fluc) activity was normalized to Renilla luciferase (Rluc), and the Fluc/Rluc ratio was calculated. BNP treatment significantly increased activity compared with PBS in Wt-*hNPR1* HEK293 cells, and this effect was abolished in Mt-*hNPR1* HEK293 cells. IL-13 enhanced activity showed an increase compared with PBS in Wt-h*NPR1*-HEK293 cells, but this didn't reach statistical significance.

b. HaCaT cells were treated with BNP (1 μM) in the presence or absence of the NRP1 antagonist A71915 (1 μM, 24 h) or vehicle control. *TBX20* mRNA expression was quantified by RT–qPCR and normalized to the indicated housekeeping gene. Data are presented as mean ± SEM from six independent experiments.

Data are presented as mean ± SEM. Statistical significance was determined using 2-way ANOVA Tukey's multiple comparisons test; $p < 0.05$.

Source data (**a**, **b**) are provided as a Source Data file.

Supplementary Fig. 3

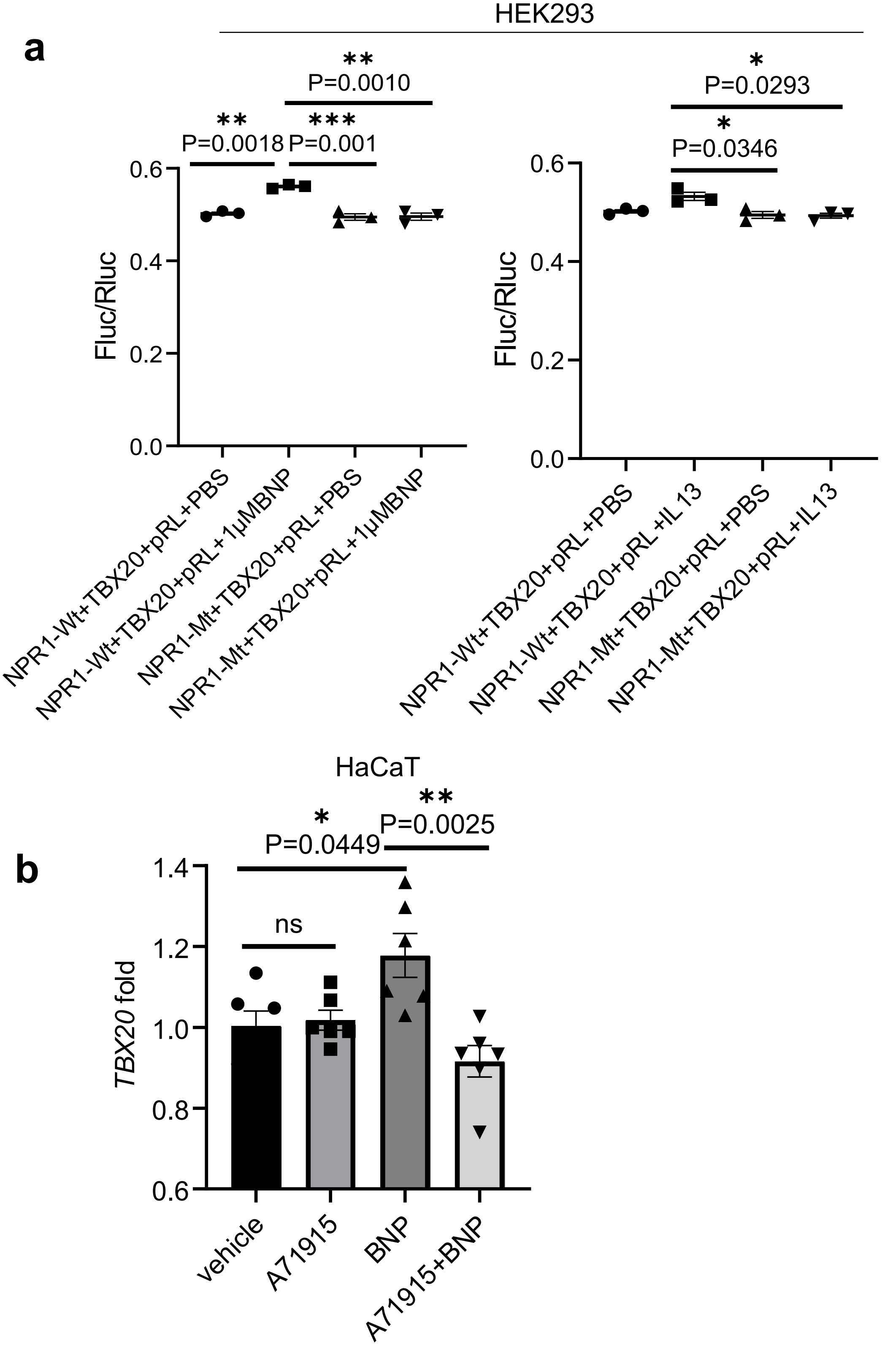

Supplementary Fig. 4. TBX20 knockdown (KD) in NHEK cells alters BNP-induced cytokine release. Immunoblot showing lentiviral TBX20 shRNA–mediated knockdown of TBX20 following independent infections with targeted and non-targeting control virus (**a**), and quantification of BNP-induced cytokine release in supernatants from TBX20-KD cells compared with non-targeting virus-treated control cells (**b**). Samples were assayed using the Proteome Profiler Human XL Cytokine Array Kit. Syntaxin 4 serves as internal control protein. Samples from three independent experiments were pooled prior to analysis. Cytokine profiling was performed in a single experiment and is presented descriptively, as no independent biological replicates were included. Each cytokine was measured in duplicate spots, and signal intensity was calculated as the mean of the duplicate spots. Error bars reflect technical variation only; no inferential statistics were performed.

Source data (**b**) are provided as a Source Data file.

Supplementary Fig. 4

a

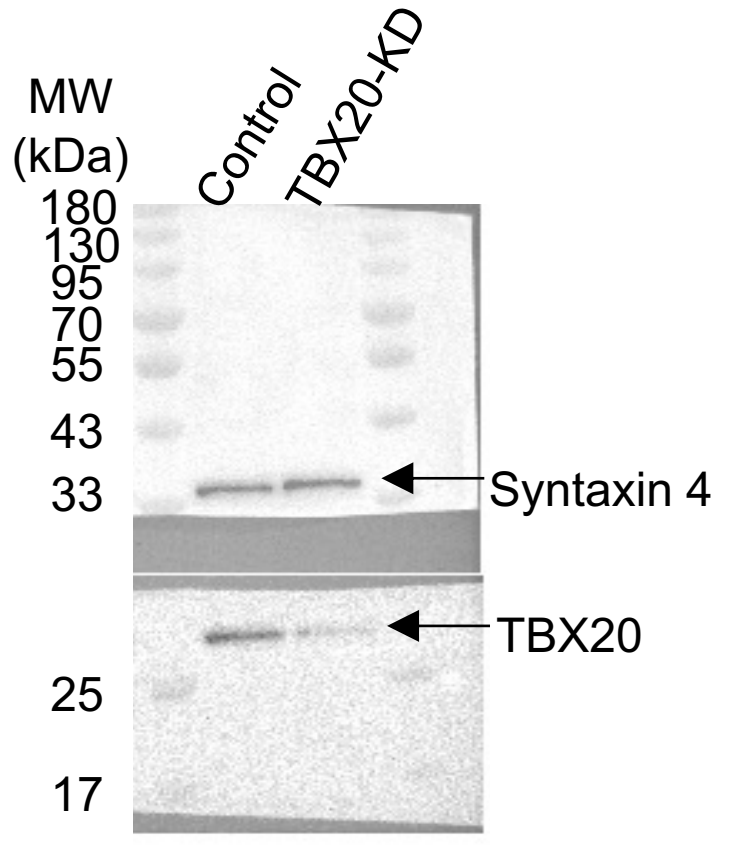

Cat: H00006810-M04, Anti-Syntaxin 4 monoclonal antibody, Novusbio

Cat: AV33177, Anti-TBX20 antibody produced in rabbit, affinity isolated antibody, Sigma

b

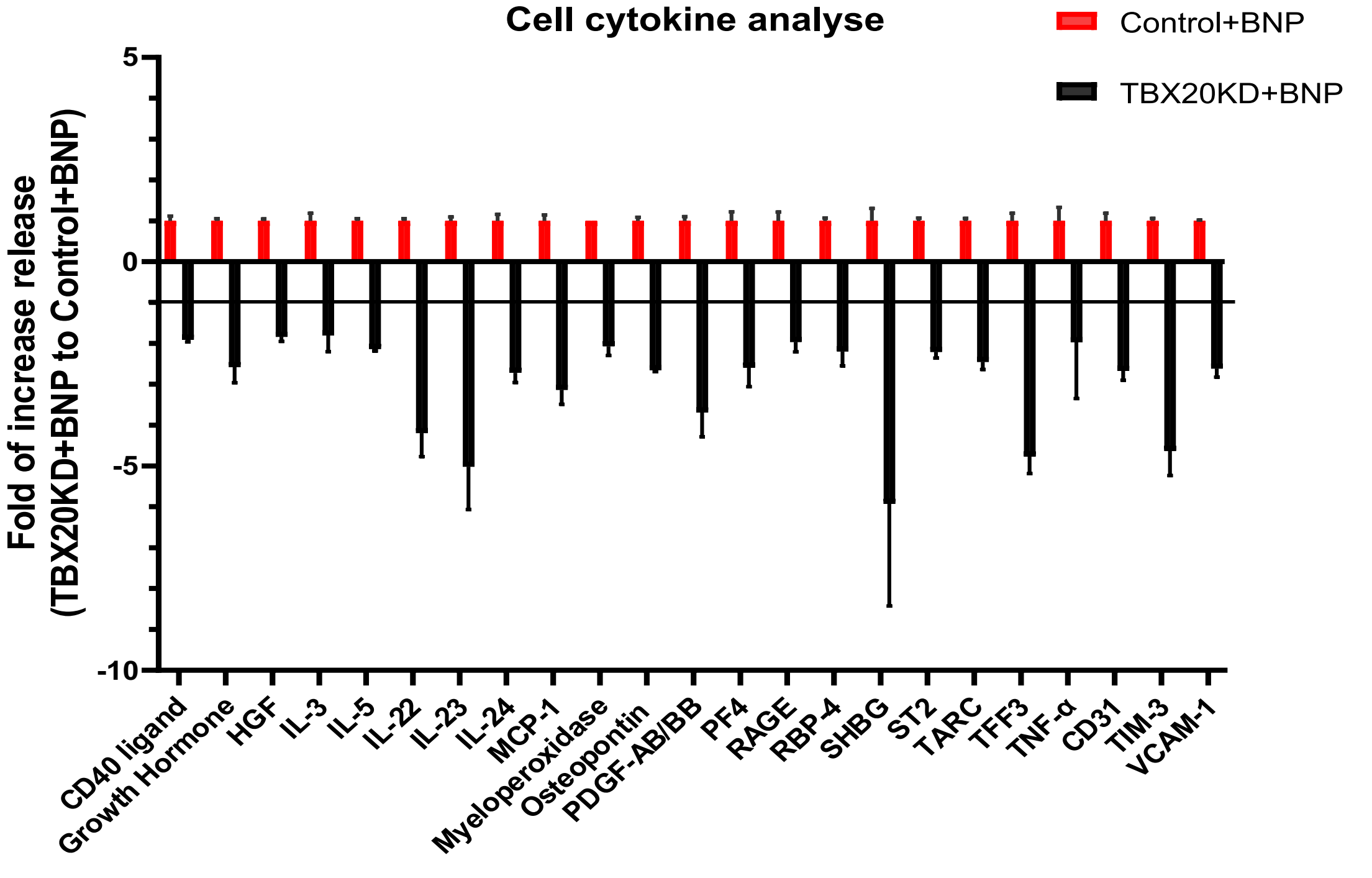

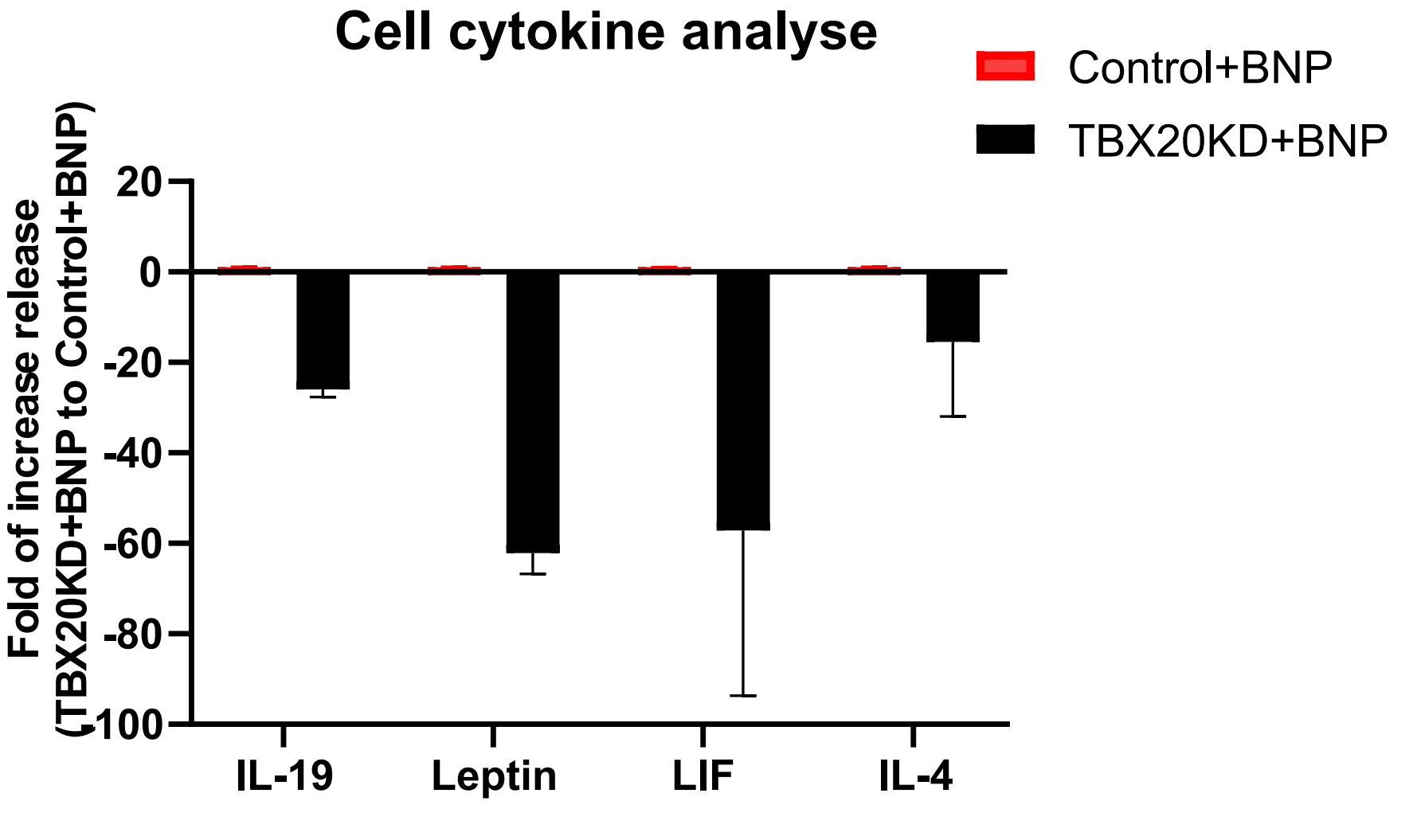

Supplementary Fig. 5. BNP triggers calcium influx in cultured normal WT murine TGNs. Depletion of TBX20 in keratinocytes did not significantly affect *Il33*, *Il25*, *Tslp*, *Ccl5, Ccl27a*, and *Ccl27b* transcription in BNP-injected cheek skin.

a. Ca^{2+}-mobilization in response to BNP (1 μM) in TGNs showed that it activates subsets. Representative trace of the Ca^{2+} transients is shown. Venn diagram shows the number of neurons activated by BNP; in all, 827 neurons were recorded from 3 independent experiments. Arrows denote a representative neuron exhibiting calcium responses. Scale bar = 50 μm.

b. FPKM comparison in BNP-injected cheek skin of $TBX20^{fl/fl}$;K14-Cre mice compared to $TBX20^{fl/fl}$ mice. N=4 mice/group were analysed.

For **b**, data are mean ± SEM; *P < 0.05, ****P < 0.0001 (Student's two tailed unpaired t test).

Source data (**a** and **b**) are provided as a Source Data file.

Supplementary Fig. 5

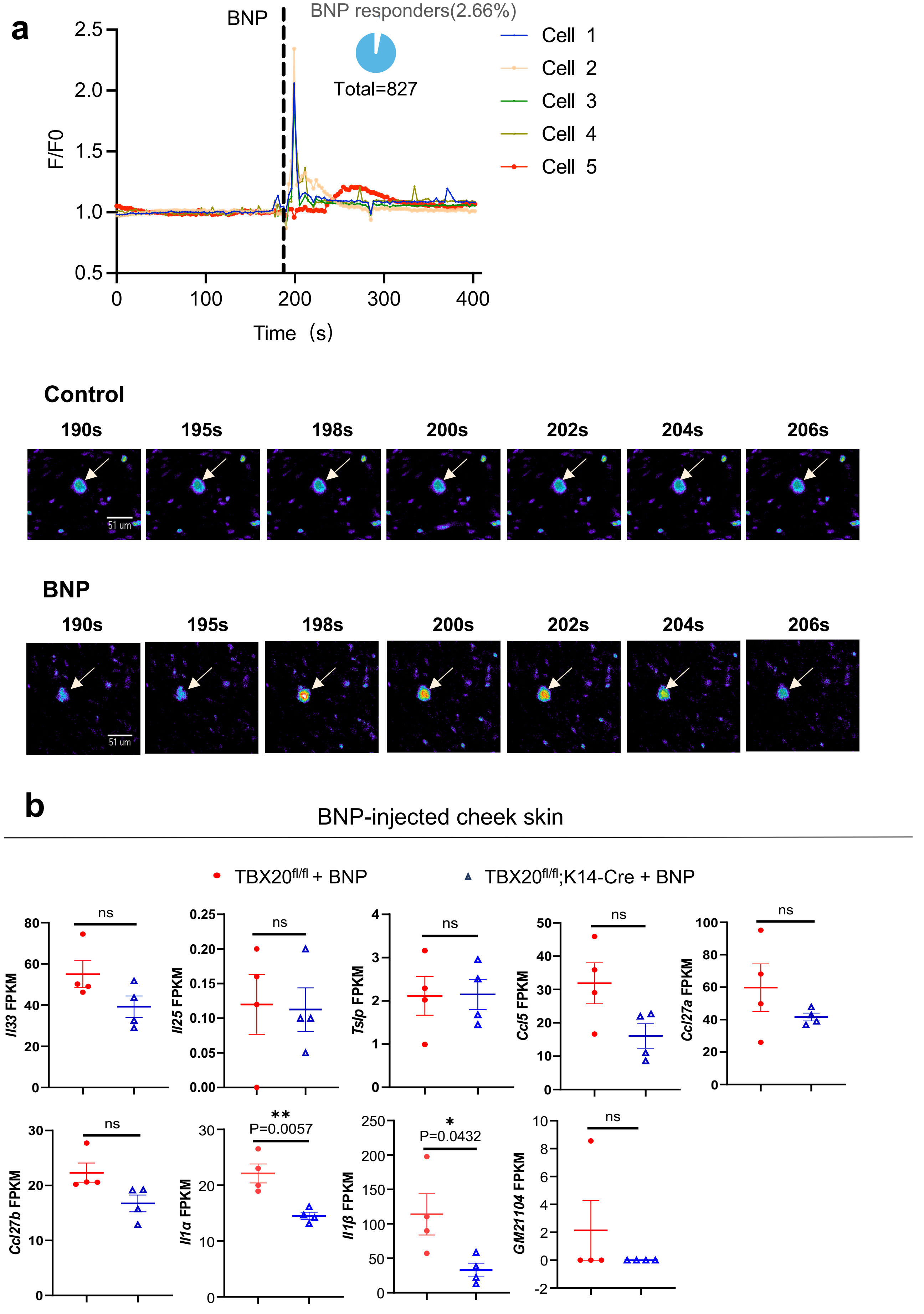

Supplementary Fig. 6. TBX20-dependent keratinocyte paracrine signalling enhances TGN calcium responses to BNP-stimulated conditioned media.

a, **b**. Conditioned media from BNP-stimulated $TBX20^{fl/fl}$ keratinocytes (**a**) elicit stronger peak calcium responses in TGNs than media from $TBX20^{fl/fl}$;K14-Cre keratinocytes (**b**). Venn diagrams depict the % of responsive neurons activated by BNP; total number of neurons were indicated from n=3 independent experiments.

c. Comparison of peak of calcium transients in the responsive neurons between two groups. N=7 neurons treated with conditioned media from $TBX20^{fl/fl}$ keratinocytes and n=6 neurons treated with conditioned media from $TBX20^{fl/fl}$;K14-Cre keratinocytes.

d. Representative trace of the Ca^{2+} transients is shown. Arrows denote a representative neuron exhibiting calcium responses. Scale bar = 50 μm.

Data presented as Mean ± SEM; a two tailed unpaired Student's t-test (**c**) was used. ****P < 0.0001.

Source data (**a**, **b** and **c**) are provided as a Source Data file.

Supplementary Fig. 6

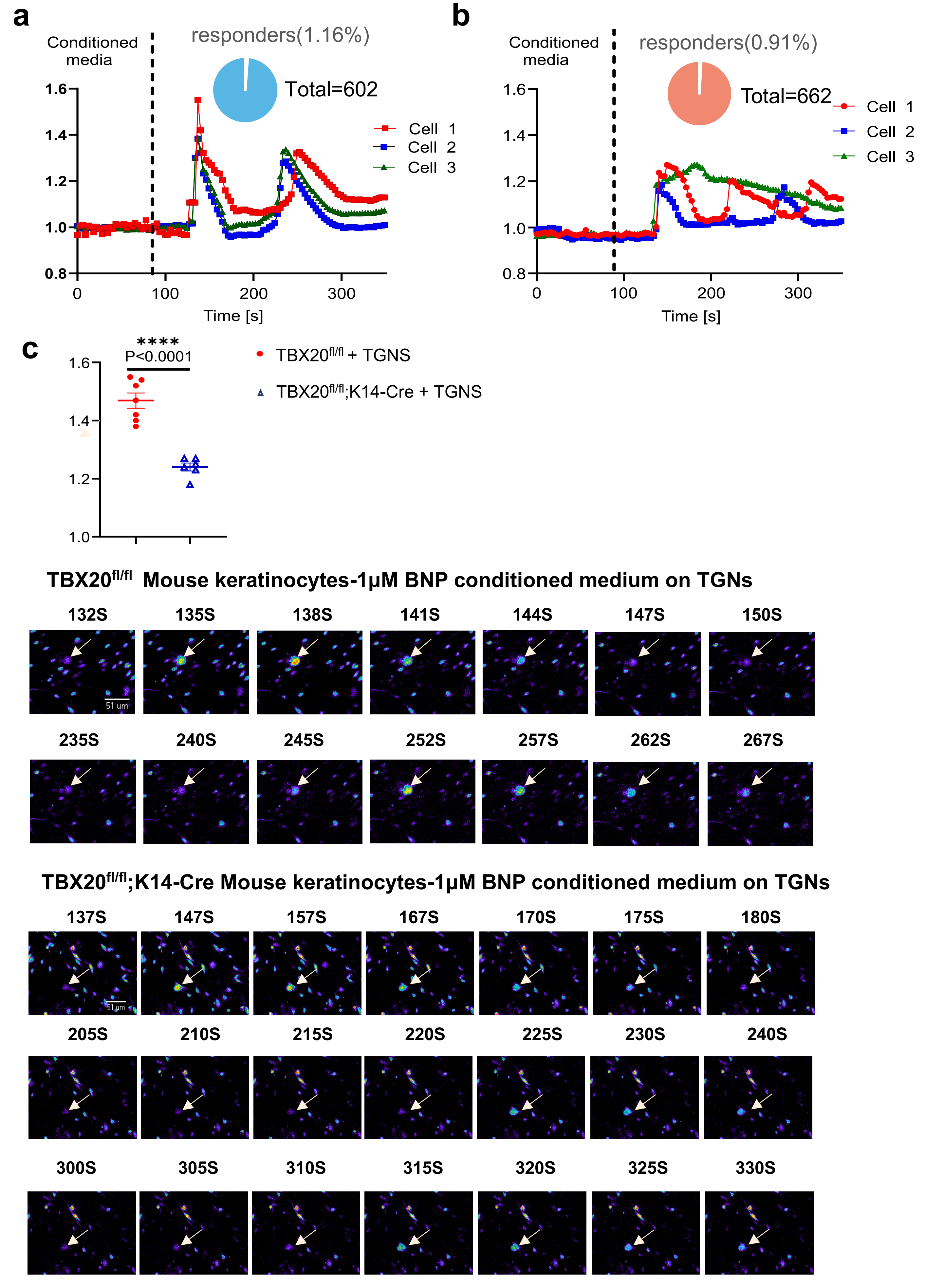

Supplementary Fig. 7. MC903- induced $CD4^+$ cell infiltration is reduced in the ear model of $TBX20^{fl/fl}$;K14-Cre mice, associated with down regulation of *Il13* and *Periostin* but not *Tslp* and *Il31* in the ear skin.

a. Immunohistochemical staining of MC903-induced ear skin for $CD4^+$ cells. Bar-chart depicts immune cell infiltration in MC903-induced ear skin (n=3 mice/group). Representative images are shown. Scale bar = 50 μm.

b. RT-qPCR analysis of gene transcripts in the MC903-induced ear skin of $TBX20^{fl/fl}$;K14-Cre mice compared to $TBX20^{fl/fl}$ control mice. N=5 mice/group.

Data are presented as mean ± SEM; a two tailed unpaired Student's t-test (**a**, **b**) was used; $^{ns}>0.05$, $^*<0.05$.

Source data (**a**, **b**) are provided as a Source Data file.

Supplementary Fig. 7

a

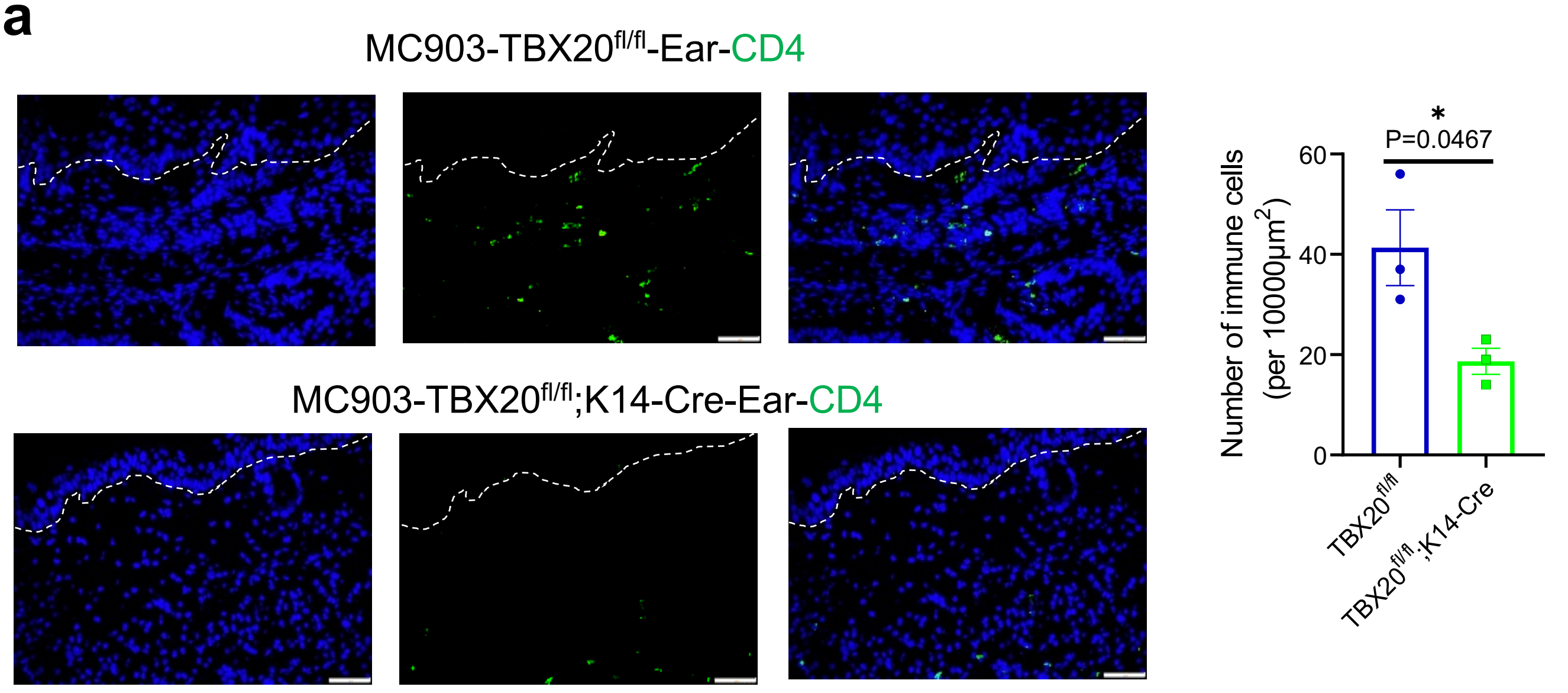

b

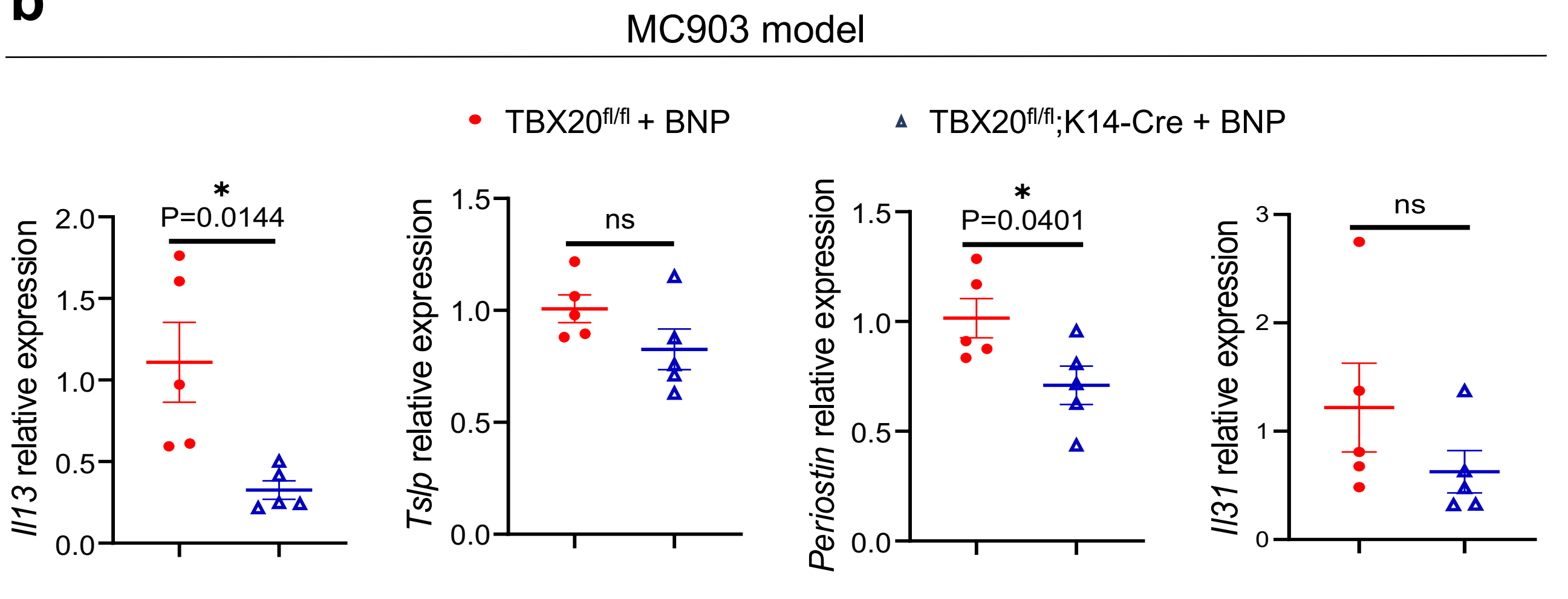

Supplementary Fig. 8. RNAscope *in situ* hybridization reveals *NPR1* and *TBX20* mRNA expression in the human DRG paraffin-embedded sections.

a. Representative images showing both *NPR1* and *TBX20* mRNA copies were detected in human DRG sections, overlapped in a fraction of cells. Merged images for the target of interest with DAPI are shown. Bar = 25 μm. Venn diagram showing the relative numbers of positive cells and a bar chart displaying the relative frequency (%). N=3 human DRG sections.

b.Representative images showing RNAScope of positive and negative controls: positive control probes are (mouse) Polr2a-C1, Ppib-C2 and UBC-C3; negative control probes is bacterial DapB-C1. Scale bar = 25 μm.

Data are presented as mean ± SEM; two tailed unpaired t test (**a**) were used. **<0.01.

Source data (**b**) are provided as a Source Data file.

Supplementary Fig. 8

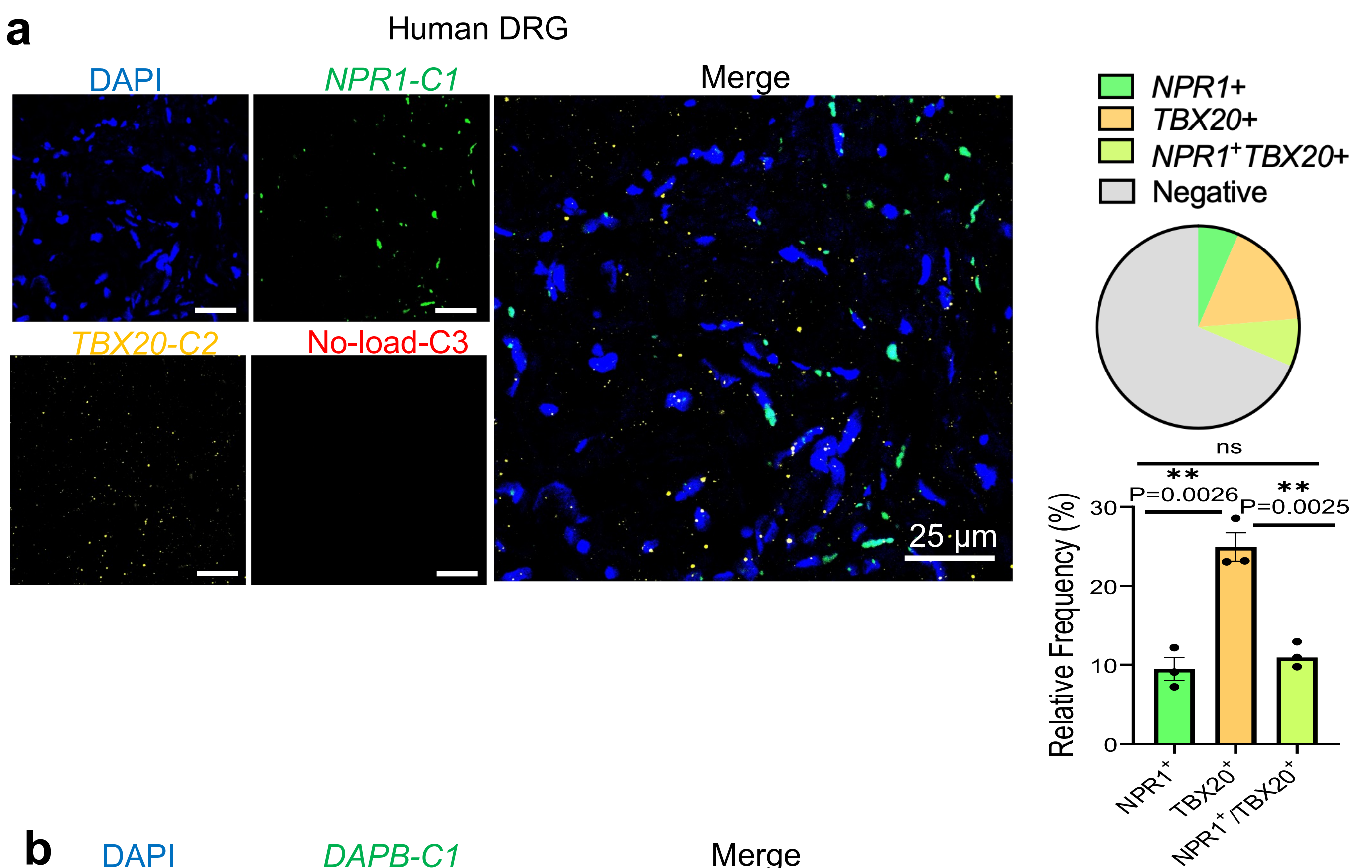

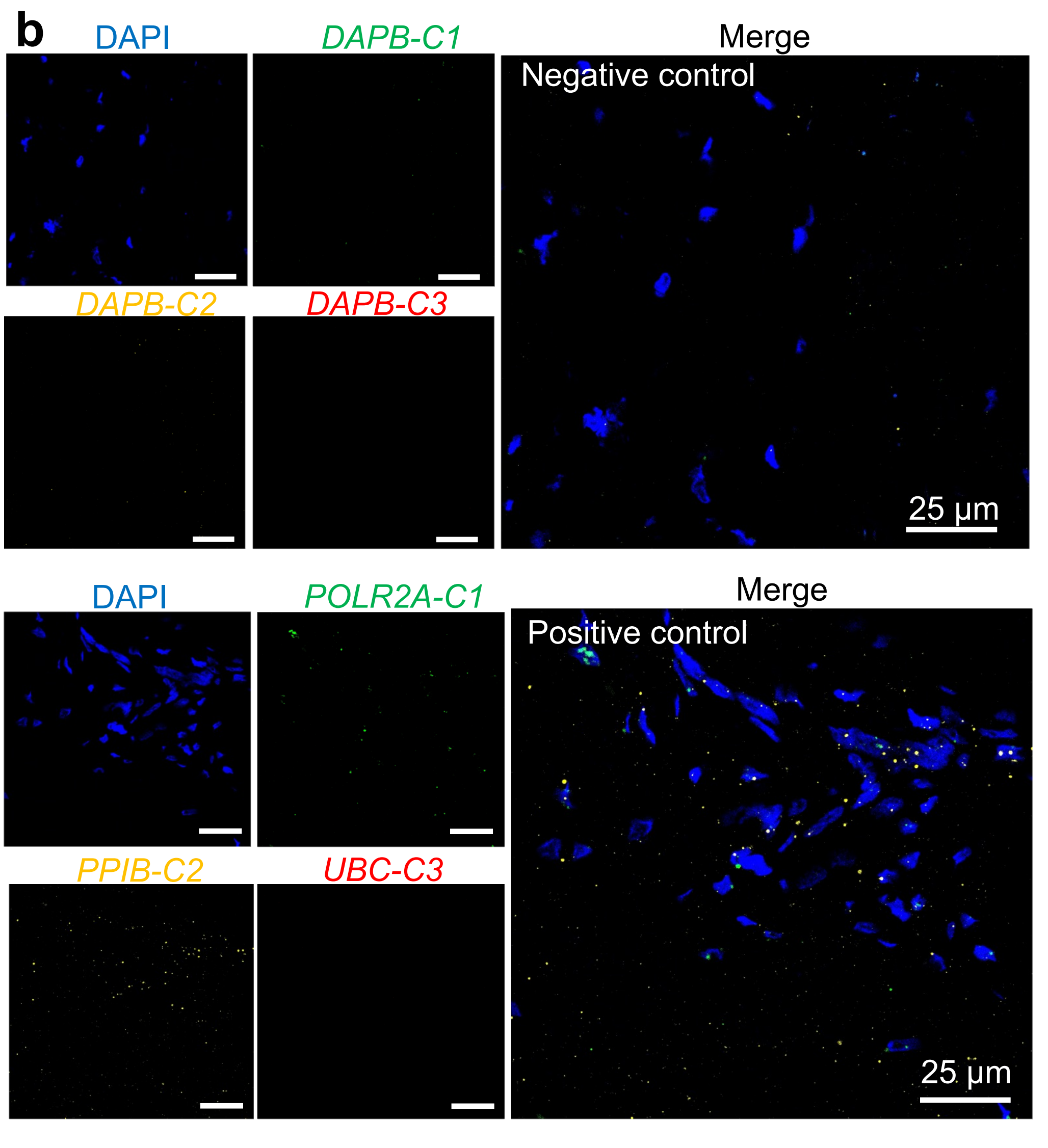

Supplementary Fig. 9. Representative images showing RNAscope positive and negative controls on mouse TG. Positive control probes (*Polr2a*, *Ppib*, and *UBC*) show robust labelling, whereas the negative control probe (bacterial *DapB*) shows no signal. Scale bar = 50 μm.

Supplementary Fig. 9

Mouse TG (DAPI/Mm-520/Mm-570)

Positive

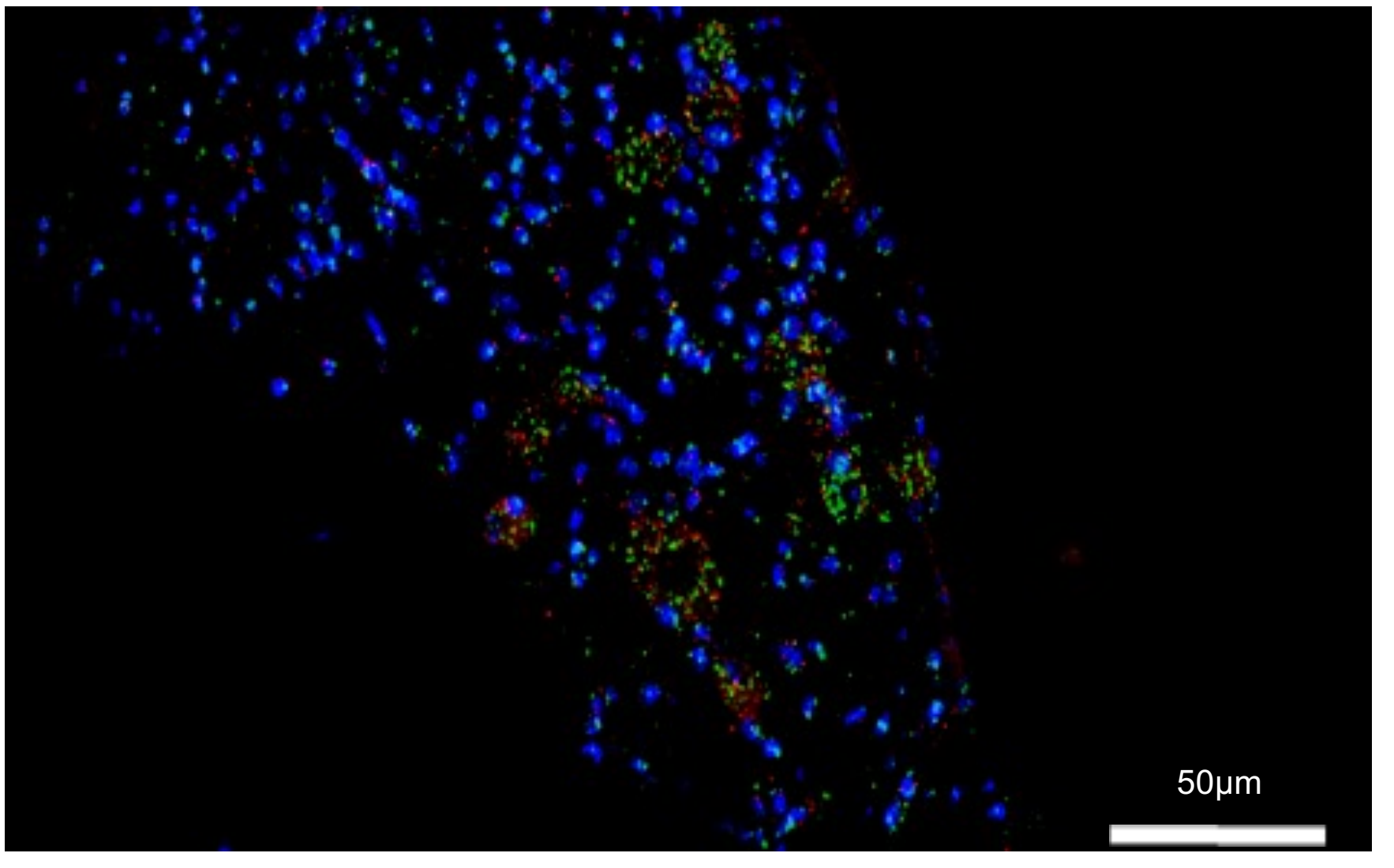

Negative

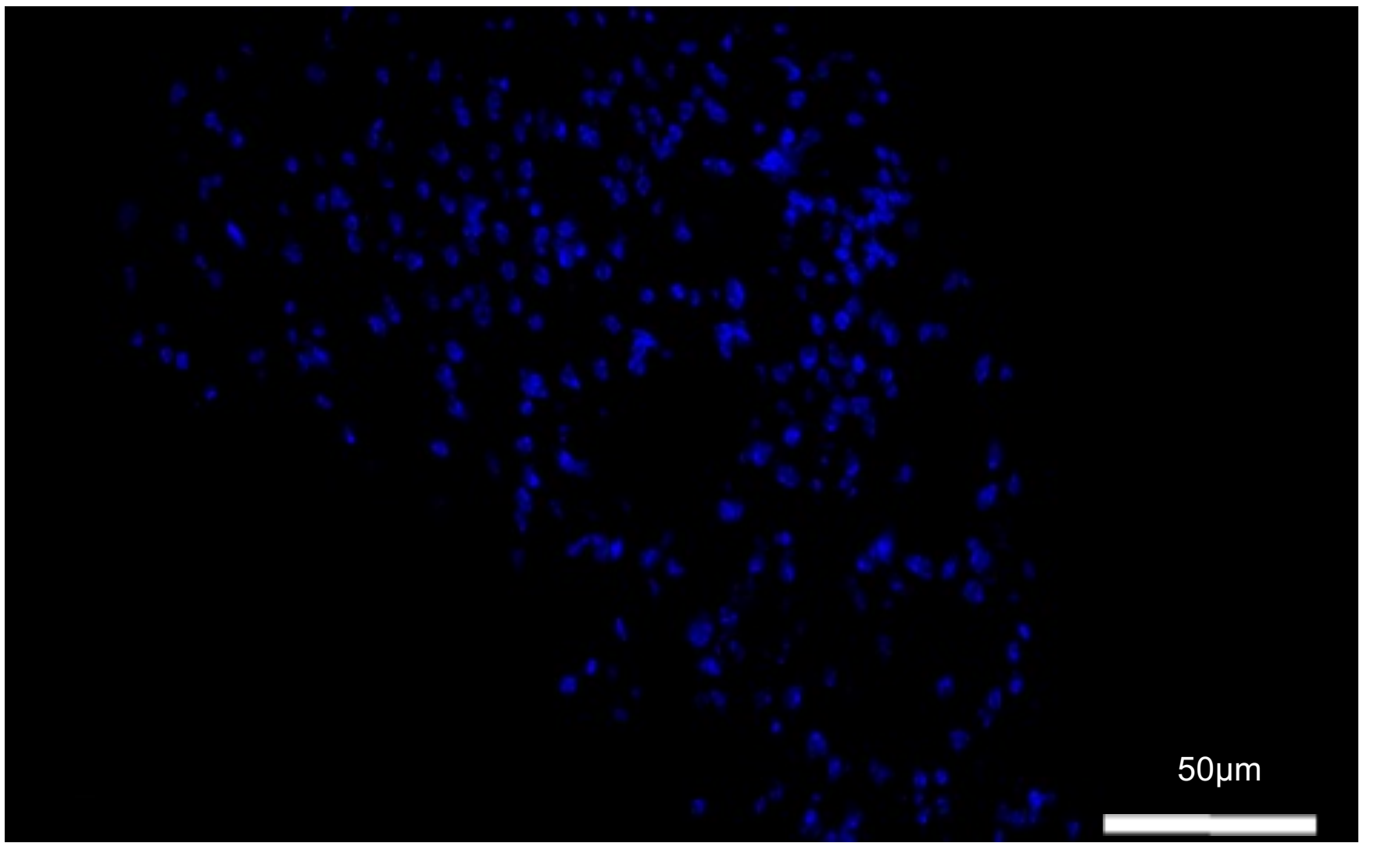

Supplementary Table 1: Sequences of primers (OriGene) used for the RT-qPCR.

genes	primers
Gapdh-M	F: CATCACTGCCACCCAGAAGACTG<br>R: ATGCCAGTGAGCTTCCCGTTCAG
Il19-M	F: CTGTGGACATGCGCCTCATAGA<br>R: TGAGGTTCTCCAGGGACAGGAT
Il20-M	F: TTCTGAGATTCGGGATAGTGTGC<br>R: GATGACGAAGGAAGCAGCACCT
Il24-M	F: CGGCTTCACTTTAGGACCCTAG<br>R: CCCAAATCGGAACTCTTGACCC
Il27-M	F: TCTCGATTGCCAGGAGTGAACC<br>R: AGTGTGGTAGCGAGGAAGCAGA
Lcn2-M	F: CCCAGCGAAGAGATGGCTAA<br>R: AAATGACACGCGGATGGTGG
Mcpt8- M	F: GGTTTCCTGGTGGCAAGAGACA<br>R: GGCTTCACTGACAGGAATGAGC
Npr1-M	F: TTCCACACTGGAGGTTCTGGCT<br>R: CTCTGAGACCAGCTCCTTTTCC
Nppb-M	F: TCCTAGCCAGTCTCCAGAGCAA<br>R: GGTCCTTCAAGAGCTGTCTCTG
Osm-M	F: CAATCGTGGCTGCTCCAACTCT<br>R: GGTGTGTTCAGGTTTTGGAGGC
S100a8-M	F: CAAGGAAATCACCATGCCCTCTA<br>R: ACCATCGCAAGGAACTCCTCGA
S100a9-M	F: TGGTGGAAGCACAGTTGGCAAC<br>R: CAGCATCATACACTCCTCAAAGC
Tbx20-M	F: CCCAGCGAAGAGATGGCTAA<br>R: AAATGACACGCGGATGGTGG
Il4-M	F: ATCATCGGCATTTTGAACGAGGTC<br>R: ACCTTGGAAGCCCTACAGACGA
Il17a-M	F: CAGACTACCTCAACCGTTCCAC<br>R: TCCAGCTTTCCCTCCGCATTGA
GAPDH-H	F: GTCTCCTCTGACTTCAACAGCG<br>R: ACCACCCTGTTGCTGTAGCCAA
TBX20-H	F: GATTCCTCCAGGCTCACTGACA<br>R: TCTTCTCCTCCGTAGGTACGGA
NPR1-H	F: CAACCTCGTGGCTGTGAAACGT<br>R: TCCCACAAACCTGGTCAGGTGT
Tlr1-M	F: GGTAGCAAGAGAAGTGGTGGAG<br>R: CGATGGTGACAGTCAGCAGAAC
Il13ra1-M	F: TGTTCTTGCCGACGCTGTCTA<br>R: GTGTAGAAGGTGGAGTTTTGCTC
Il13ra2-M	F: TGGTCTGCTCTTGGAAACCTGG<br>R: GCTGGAGGTAATCAGCACACTG
Il31ra-M	F: ACACCGAGTTGGAGAGCCGTAT<br>R: CTGTCCTCAGACCGATGTTCTC
Il13-M	F: AACGGCAGCATGGTATGGAGTG<br>R: TGGGTCCTGTAGATGGCATTGC
Il31-M	F: CCACACAGGAACAACGAAGCCT<br>R: CCCGGTCCAGGCTGAAACACG
Tslp-M	F: GCAAATCGAGGACTGTGAGAGC<br>R: TGAGGGCTTCTCTTGTTCTCCG
Periostin-M	F: CAGCAAACCACTTTCACCGACC<br>R: AGAAGGCGTTGGTCCATGCTCA

Supplementary Table 2: Antibodies used for immunohistology.

Antibody	Cat# and dilutions	Sources
FLG2	NBP1-91901 (1:500)	Novus
LOR	905103 (1:500)	Biolegend
PGP9.5	AB108186 (1:500)	Abcam
CD11b	AB13357 (1:500)	Abcam
MCPT8	647401 (1:500)	Biolegend
CD4	AB183685 (1:200)	Abcam
CD68	AB283654 (1:100)	Abcam
PGP9.5	AB8189 (1:500)	Abcam
IL-31RA	AF2107 (1:200)	R&D
TBX20	AV33177 (1:100)	Sigma-Aldrich
K14	NBP2-34355(1:500)	Novus
LCN-2	PA5-116987 (1:250)	Thermo Fisher
Goat Anti-Rat 488	AB150165 (1:500)	Abcam
Goat Anti-Rabbit 488	AB150077 (1:500)	Abcam
Goat Anti-Rat 594	AB150160 (1:500)	Abcam
Goat Anti-Mouse 594	AB150111 (1:500)	Abcam
Goat Anti-Mouse 488	AB150133 (1:500)	Abcam

Supplementary Table 3: Reagents used for RNAscope in human skin and murine TG.

Reagents	Company	Catalogue
RNAscope™ Multiplex Fluorescent Reagent Kit v2	ACD	323100
RNAscope™ Probe - Hs-TBX20-C2	ACD	868311-C2
RNAscope™ Probe - Hs-NPR1	ACD	452191
RNAscope™ Probe - Mm-Nppb-C2	ACD	425021-C2
RNAscope™ Probe - Mm-Npr1	ACD	484531
RNAscope™ 3-plex Negative Control Probe	ACD	320871
RNAscope™ 3-plex Positive Control Probe - Hs	ACD	320861
RNAscope™ 3-plex Positive Control Probe - Mm	ACD	320881
TSA Vivid 520	AXB (AlphaX (Beijing) Biotech Co., Ltd))	AXT6210000
TSA Vivid 690	AXB	AXT6705000

Supplementary Table 4: Probes used for RNAscope in human DRG sections.

Reagents	Company	Catalogue
RNAscope™ Probe - Hs-TBX20-C2	ACD	868311-C2
RNAscope™ Probe - Hs-NPR1	ACD	452191
RNAscope™ 3-plex Negative Control Probe	ACD	320871
RNAscope™ 3-plex Positive Control Probe	ACD	320861
TSA Vivid 520	ACD	323271
TSA Vivid 570	ACD	323272
TSA Vivid 650	ACD	323273
