# Supplementary material for: Misregulation of T-box transcription factors drives BNP–NPR1 signalling underlying chronic itch
Source: Nat Commun. 2026 Jul 17;17:8754. doi: 10.1038/s41467-026-75726-x (PMC13493844; doi:10.1038/s41467-026-75726-x)
Supplement: Supplementary file 2 [file 41467_2026_75726_MOESM2_ESM.pdf]

nature portfolio

Peer Review File

Misregulation of T-box transcription factors drives BNP–NPR1 signalling underlying chronic itch

Corresponding Author: Dr Jianghui Meng

This file contains all reviewer reports in order by version, followed by all author rebuttals in order by version.

Version 0:

Reviewer comments:

Reviewer #1

(Remarks to the Author)
The team identified the T-box transcription factor 20 (TBX20) and the BNP receptor NPR1 as key regulators of chronic itch and skin barrier dysfunction in atopic dermatitis (AD). The authors demonstrate that TBX20 is upregulated in the skin of AD patients and directly binds to and activates the NPR1 gene. Using a series of knockout mouse models, the authors show that deleting TBX20 in either keratinocytes or sensory neurons reduces AD-associated itch, decreases inflammation (basophil infiltration, IL-4, and Oncostatin M levels), and restores skin barrier integrity. The study further identifies LCN-2 as a downstream effector of this pathway. Finally, a rescue experiment using an AAV approach confirmed that the TBX20-NPR1 axis is a crucial driver of chronic itch, positioning it as a promising new therapeutic target for inflammatory skin disorders.
Several critical gaps in the proposed TBX20-NPR1 axis weaken the authors' conclusion.
Major comments:
The ChIP assay, while useful, only demonstrates physical binding and does not prove that this interaction leads to gene activation. A reporter assay will be helpful to establish that connection.

The claim that LCN-2 is a downstream effector of the TBX20-NPR1 axis is not fully substantiated. The study shows LCN-2 is downstream of TBX20, but the essential link between NPR1 signaling and LCN-2 expression is missing, undermining the proposed pathway.

Lacks a direct demonstration of the functional crosstalk between keratinocytes and sensory neurons. While knockout models show that deleting TBX20 in either cell type reduces itch, the study does not provide direct evidence (e.g., through co-culture experiments) to show how one cell type's actions influence the other. At least, some functional assessment of BNP-NPR1 in keratinocytes or in sensory neurons would be helpful.

Technical and Data Presentation issues undermine the reliability of the findings, such as the skin IHC provided in Figure 1. Others are of insufficient quality, and the lack of high-resolution confocal images makes it difficult to verify key localizations. The staining for PGP9.5 and NPR1 around the edges appears to be an artifact rather than the expected staining, which raises questions about the technique or antibody specificity. The antibody specificity for NPR1 in TG (Trigeminal Ganglion) tissue is a significant concern. The authors should have performed in situ hybridization (ISH) to provide independent confirmation of NPR1 expression. Additionally, they should have co-stained for known itch markers in the DRG to identify the specific neuronal population expressing NPR1 more accurately.

AD is a model of type 2 inflammatory itch, yet it fails to show an upregulation of several key itch cytokines known to be involved in AD, such as TSLP, IL-4, IL-13, and periostin. Lastly, the authors' assumption that BNP's effect works directly through sensory neurons is speculative, as the possibility of an indirect mechanism remains unaddressed.

(Remarks on code availability)

Reviewer #2

(Remarks to the Author)
The paper presents novel insights into chronic itch conditions by investigating the role of BNP-NPR1 axis in keratinocytes and sensory neurons. The authors conducted cell-based assays, analysis of clinical samples along with generation of transcription factor-deficient mice to study the role of TBX20 in keratinocytes and sensory neurons in AD and ACD mouse models. Based on the results, the authors suggest that TBX20 plays an important role in itch regulation and modulates immune response and skin barrier integrity.

However, there are certain aspects that need more specific explanation and clarification.

Major comments:
1. When conducting behavioral experiments, what was the reason of using male LCN-2 KO mice, and not female ones? As the behavioral patterns of male and female mice may vary, the mice of the same sex would give more consistent results. Additionally, through the whole paper there was no behavioral data for WT mice treated with PBS and MC903/DNFB. I would suggest to add this data to see the difference in behavioral phenotype between WT and transgenic mice.
2. For 'Animal Models and Itch Behavior Scoring' section – please provide the references for all models. Additionally, for chronic itch models (MC903 and DNFB) – it is stated that 'immediately scratching behaviors were recorded for 60 min'. Does it mean that the experiments were conducted immediately after the application of MC903 and DNFB? Given that the authors aimed at investigating the chronic itch, have you tried to check the itch phenotype 24h-post exposure or before daily treatment?
3. For the behavioral experiments, the duration is not consistent which makes it hard to interpret the data. Could the authors clarify how the duration was defined?
4. For Figure 1F, the authors aimed at assessing the co-localization of TBX20 and NPR1-expressing keratinocytes (KC), however, the KC marker was not used (e.g. Krt14). Additionally, how many KC co-express TBX20 and NPR1? The quantification would support your data. Also, how many sample were used?
5. For Figure 3C and 3E, please provide the references validating the marker panel used. How were these markers selected? Also, in AD context it is known that KC release important itch-related cytokines and chemokines such as IL-33, IL-25, TSLP, IL-1, CCL5, CCL27 etc. Have the authors tried to analyze them? Additionally, for Figure 3E please revise the groups: according to the text, the groups are 'TBX20fl/fl + BNP', and 'TBX20fl/fl; K14-cre + BNP', otherwise it is confusing.
6. Could the authors clarify the rationale for focusing on macrophages and basophils in Figures 4D? Were other relevant populations (e.g., TH2, TH17, TH1, Treg, dendritic cells, mast cells, neutrophils) analyzed, and if so, what were the results? If not, please justify their exclusion. Additionally, please justify how the markers were defined in Figure 4E. Have the authors tried to analyze IL-13 and IL-31 (which are also critical mediator of itch)?
7. For Figure 5D – please provide the references for the marker panel. How were these markers selected?
8. For Figure S1A, the KC marker was not used to support the statement that 'Lcn-2 was specifically increased in epidermal KC'.
9. In line 553, it was stated that knock-out of TBX20 in KC led to a significant downregulation of Lcn-2 transcription compared to other genes. Please provide statistical results for the other genes to support this claim. In Supplementary Fig. S1B, Saa3 and Ms4a4c also appear decreased—do these changes reach statistical significance? Additionally, have the authors tried to analyze Lcn-2 target cells?
10. According to 'GSE281758_core_table_gene', acute-phase protein serum amyloid genes (Saa1, Saa2, Saa3) are also significantly upregulated along with Il1b. The relationship between SAA proteins and IL-1β has already been investigated in psoriatic (IMQ-induced) skin inflammation (Yu N et al. Clin Exp Immunol. 2015). Have the authors considered to analyze this axis in relation to dysregulation of TBX20/NPR1 in AD and ACD context? Could the authors explain the preference of Lcn2 over Saa and IL-1β genes?
11. For Figures S1E and S1F, please explain the difference in duration of experiments as it can affect the data interpretation. Please clarify whether the skin dissection time point was kept constant across all experiments. Additionally, does the deficiency of Lcn-2 affect Mcpt8+ population?
12. In Figure 7B – the graph is not clear - total scratching bouts on day 13?
13. Figure 7H – the data for Mcpt8+ cells is confusing – in comparison with Figure 6A and Figure 4D, the number of Mcpt8+ cells in Figure 7H vehicle group is sharply decreased – how can it be explained? Does the genetic background of mice (WT vs TBX20fl/fl) affect the result?
14. Figure 7A – have the authors tried to use AAV-mediated Tbx20 rescue in sensory neurons? If not, please explain why rescue was restricted to keratinocytes?
15. According to RT-qPCR results, IL-13 significantly upregulated Tbx20 and Npr1 transcription in NHEK highlighting the importance of interaction between IL-13 and KC – however, IL13 and IL-13-producing cells-related data is lacking in this study. I would suggest to investigate the change of this cytokine and related cells in the context of TBX20 deficiency.
16. In Discussion part, the authors did not mention basophils (Mcpt8+ cells), although the Mcpt8 data was provided across the study – please discuss the importance of basophils in the context of TBX20-NPR1 axis underlying chronic itch.

Minor comments:
1. What serotype of AAV was used in your experiments? Please indicate it.
2. For Figure 3A and B and the corresponding paragraph (lines 509-520), I suggest you to exclude the TBX20fl/fl-Avil-cre and insert it to another Figure which contains TBX20fl/fl-Avil-cre data (Figure 7) as it is confusing.
3. Line 549 – please paraphrase the subtitle (check the grammar)
4. For line 559 – i.d. injection of Lcn-2 to WT mice? Please specify it.
5. For Figure S1E and S1F, I would suggest to add 'MC903' and 'DNFB' accordingly for easier understanding.
6. Line 620 – the sentence is confusing – immunostaining of skin or TG? Please revise it.

(Remarks on code availability)
When trying to reach the code/raw data, the following message appears on SRA Run Selector: 'nothing has been found for accession'. The only file available for downloading is supplementary file in txt format. The supplementary file was reviewed and assessed accordingly.

Reviewer #3

(Remarks to the Author)

This study investigates the expression and functional role of TBX20 in lesional and non-lesional skin of atopic dermatitis (AD) patients, particularly focusing on its role in itch. While the observation is potentially interesting, the manuscript suffers from poor organization, unclear data presentation, and methodological inconsistencies, raising concerns about the quality and reliability of the conclusions. Significant improvements in organization, data interpretation, and methodological rigor are needed.

Specific Comments
1. Figure 1, Antibody staining: The TBX20 antibody staining does not appear specific. As a transcription factor, TBX20 should be localized in the nucleus, but in Figure 1A it appears cytoplasmic. Antibody specificity should be tested in knockouts and controls. In situ hybridization (ISH): The ISH signal is very weak, making it difficult to interpret. Using RNAscope or another sensitive method could improve signal detection. Figure 1F: If the antibody works, why not consistently use it across all panels instead of mixing detection methods?
2. Figure 2, The rationale for selecting H3K27ac-negative loci is unclear. The relationship to other histone modifications should be explained.
3. To support the functional relevance of the NRP1 promoter/enhancer targeted by TBX20, additional experiments are needed, such as reporter assays with TBX20 overexpression or knockdown.
4. The authors show that TBX20 and NRP1 expression are correlated, but only test whether TBX20 regulates NRP1. They should also consider testing whether NRP1 regulates TBX20.
5. ChIP vs. RT-qPCR, Page 20, lines 497–502: The text suggests the authors performed ChIP-seq to identify TBX20 binding on the NRP1 promoter. However, Figure B appears to show gene expression (RT-qPCR), not ChIP-qPCR. The authors may have mixed gene expression and ChIP experiments, or there is a misunderstanding of the techniques.
6. Figure 3B: Primer locations should be clearly labeled to indicate PCR products. Figure legends (page 30, lines 808–810) are poorly written and difficult to understand. Additional experiments such as qPCR and IHC would help confirm gene deletion in conditional knockout (CKO) mice.
7. LCN2 Focus, Page 21, line 549: The functional analysis of LCN2 is introduced without justification. Among multiple genes that are altered, it is unclear why the authors focus only on LCN2.
8. AAV Experiments, Details on the AAV vector are missing. The authors should test efficiency and confirm that TBX20 is successfully overexpressed, ideally using IHC to show tissue specificity. Control data should be shown.
9. NRG1 Treatment, Page 24, line 661: The authors state, “we employed pharmacological inhibition of TBX20 using Neuregulin-1 (NRG1),” but there is no evidence or reference supporting that NRG1 inhibits TBX20. This claim is unsupported and requires clarification.
10. The organization of the results, The manuscript mixes neural TBX20 knockout mice (TBX20^fl/fl;Avil-Cre) and skin-specific knockout mice (TBX20^fl/fl;K14-Cre). Figures 5 and 6: TBX20^fl/fl;Avil-Cre (sensory neurons). Figures 3, 4, 7: TBX20^fl/fl;K14-Cre (skin). This inconsistency makes it difficult to follow the experimental logic and interpret the results. Clear separation of the two models is required

(Remarks on code availability)

Reviewer #4

(Remarks to the Author)
I co-reviewed this manuscript with one of the reviewers who provided the listed reports. This is part of the Nature Communications initiative to facilitate training in peer review and to provide appropriate recognition for Early Career Researchers who co-review manuscripts.

(Remarks on code availability)

Version 1:

Reviewer comments:

Reviewer #1

(Remarks to the Author)
While the authors have made significant progress in addressing the previous concerns, several critical points remain. First, the Npr1 expression profile in the sensory ganglia has not been sufficiently characterized. It is essential to clarify whether the

reported expression and functional data were derived from naïve (normal) mice or atopic dermatitis models as well as the data is not convincingly demonstrated the expression of Npr1 in the sensory neurons. Additionally, to strengthen the mechanistic link, the authors should include expression data for Nppb and Npr1 in ear skin within Figure 5. Finally, regarding Figure 3C, the inclusion of siRNA-mediated knockdown as a control is necessary to validate the specificity of the various cytokine releases.

(Remarks on code availability)

Reviewer #2

(Remarks to the Author)
The authors have conducted comprehensive revision of the previous version and adequately addressed all the questions and comments made in the 1st round of review. The current version is well-organized, presented in a clear and accessible style for readers.

(Remarks on code availability)

Reviewer #3

(Remarks to the Author)
Reviewers have nicely addressed my previous concerns

(Remarks on code availability)

Reviewer #4

(Remarks to the Author)
I co-reviewed this manuscript with one of the reviewers who provided the listed reports. This is part of the Nature Communications initiative to facilitate training in peer review and to provide appropriate recognition for Early Career Researchers who co-review manuscripts.

(Remarks on code availability)

Version 2:

Reviewer comments:

Reviewer #1

(Remarks to the Author)
The authors have addressed all my concerns!

(Remarks on code availability)

Open Access This Peer Review File is licensed under a Creative Commons Attribution 4.0 International License, which permits use, sharing, adaptation, distribution and reproduction in any medium or format, as long as you give appropriate credit to the original author(s) and the source, provide a link to the Creative Commons license, and indicate if changes were made.
In cases where reviewers are anonymous, credit should be given to 'Anonymous Referee' and the source.
The images or other third party material in this Peer Review File are included in the article's Creative Commons license, unless indicated otherwise in a credit line to the material. If material is not included in the article's Creative Commons license and your intended use is not permitted by statutory regulation or exceeds the permitted use, you will need to obtain permission directly from the copyright holder.
To view a copy of this license, visit https://creativecommons.org/licenses/by/4.0/

We are submitting the revised manuscript (NCOMMS-25-60793A), entitled "Misregulation of T-box transcription factors drives BNP–NPR1 signalling underlying chronic itch" upon Reviewers' thoughtful suggestions. We have revised the manuscript based upon the comments, which we feel has greatly improved the manuscript. Detailed point-to-point responses are described below. With these revisions (highlighted in yellow in the marked copy of the revision), we hope this manuscript is now acceptable for Nature Communications.

COMMENTS FROM REVIEWER #1

Reviewer #1: (Remarks to the Author):

The team identified the T-box transcription factor 20 (TBX20) and the BNP receptor NPR1 as key regulators of chronic itch and skin barrier dysfunction in atopic dermatitis (AD). The authors demonstrate that TBX20 is upregulated in the skin of AD patients and directly binds to and activates the NPR1 gene. Using a series of knockout mouse models, the authors show that deleting TBX20 in either keratinocytes or sensory neurons reduces AD-associated itch, decreases inflammation (basophil infiltration, IL-4, and Oncostatin M levels), and restores skin barrier integrity. The study further identifies LCN-2 as a downstream effector of this pathway. Finally, a rescue experiment using an AAV approach confirmed that the TBX20-NPR1 axis is a crucial driver of chronic itch, positioning it as a promising new therapeutic target for inflammatory skin disorders. Several critical gaps in the proposed TBX20-NPR1 axis weaken the authors' conclusion.

Response: We greatly appreciate the reviewer's comments and valuable suggestions to improve our manuscript. According to the Reviewers' suggestions, we performed additional experiments and revised the manuscript.

Major comments:

1) The ChIP assay, while useful, only demonstrates physical binding and does not prove that this interaction leads to gene activation. A reporter assay will be helpful to establish that connection.

Response: Thank you for raising this important suggestion. To address this question, we performed Dual-Luciferase Reporter Assay.

Firstly, binding sites of *hTBX20* and *hNPR1* gene were predicted using the JASPAR database based on *hTBX20* matrix profile MA0689.1. The potential interaction sites identified on *hNPR1* promoter include 1333-1343bp, 1433-1443bp, 1625-1635bp, and 1854-1864bp. We then synthesized both wild-type (Wt) and mutant (Mt) promoter fragments of *hNPR1*, in which the Mt contained all four mutations, and the dual-luciferase reporter vector system was used to assess promoter activity and promoter–transcription factor interactions. The pGL3-Basic plasmid was cloned with Wt or Mt *hNPR1* promoter fragment upstream of the firefly luciferase (Fluc) gene (**Supplementary Fig. 2**); the pcDNA3.1 plasmid was used for *hTBX20* CDS region (NM001077653.2) overexpression; the pRL-TK plasmid was used for expressing Renilla luciferase (Rluc) as an internal control.

For luciferase assays, we co-transfected all three plasmids into both the human keratinocyte cell line HaCaT and HEK293 cells. After 48 h of incubation, cells were

treated with 1 μM BNP vs vehicle (PBS) for 24 hours, lysed and Firefly and Renilla luciferase activities were measured using the Dual-Luciferase® Reporter Assay System, with promoter activity evaluated as the Fluc/Rluc ratio.

Dual-luciferase assays showed that *TBX20* significantly increased Wt *hNPR1* promoter activity following BNP stimulation compared to vehicle treatment, whereas mutation of the predicted binding sites abolished this increment, indicating a specific transcriptional interaction between TBX20 and the *hNPR1* promoter. This result has been included in the revised manuscript as **Fig. 2c** and the **Supplementary Fig. 3a**.

Accordingly, we have revised our manuscript as follows. In the main text, the revisions are highlighted in yellow.

(P23L597-616) "To investigate whether TBX20 directly regulates *NPR1* transcription, a dual-luciferase reporter assay was performed. The human NPR1 promoter region (−2000 to +200 bp relative to the transcription start site [TSS]; NC_000001:153676688–153678887) was cloned upstream of the firefly luciferase gene in the pGL3-Basic vector to generate the wild-type reporter construct (**Supplementary Fig. 2**).

Four putative TBX20-binding sites within the NPR1 promoter were predicted using JASPAR (MA0689.1; relative score > 0.8, score > 8), located at positions 1333–1343 bp (0.94; 14.7), 1433–1443 bp (0.87; 10.5), 1625–1635 bp (0.85; 9.6), and 1854–1864 bp (0.81; 7.4). To assess the functional relevance of these sites, a mutant NPR1 promoter fragment harbouring simultaneous mutations at all four predicted TBX20-binding sites was synthesized (**Supplementary Fig. 2**) and cloned into the same reporter vector.

HaCaT cells were co-transfected with either the wild-type or mutant NPR1 promoter construct, a TBX20 overexpression plasmid (pcDNA3.1-TBX20), and the Renilla luciferase plasmid pRL-TK as an internal control. Promoter activity was determined by calculating the ratio of firefly to Renilla luciferase activity (Fluc/Rluc).

Dual-luciferase assays showed that TBX20 significantly enhanced *NPR1* promoter activity following BNP stimulation in HaCaT cells transfected with the wild-type construct. In contrast, the synthesized mutant promoter construct abolished this activation, indicating that TBX20 regulates NPR1 transcription through these specific binding sites (**Fig. 2c**). Similar results were observed in HEK293 cells (**Supplementary Fig. 3a**)."

2) The claim that LCN-2 is a downstream effector of the TBX20-NPR1 axis is not fully substantiated. The study shows LCN-2 is downstream of TBX20, but the essential link between NPR1 signaling and LCN-2 expression is missing, undermining the proposed pathway.

Response: Thank you for this comment. To address this, we examined the effects of Lcn-2 deficiency on the transcription of *Nppb* and its receptor *Npr1* in the skin of MC903-treated mice. RT-qPCR analysis showed significant downregulation of both *Nppb* and *Npr1* transcripts in Lcn-2 KO mice compared with WT controls in the MC903 model. These findings suggests that Lcn-2 influences the Nppb–Npr1 axis, linking Lcn-2 activity to NPR1 signalling, particularly in MC903 model and supporting

its role as a downstream effector of the TBX20–NPR1 pathway. The corresponding data have been added to the revised manuscript as **Fig. 5c**.

Accordingly, we have revised our manuscript as follows. In the main text, the revisions are highlighted in yellow.

(P27L724-726) " In ear skin, transcription of *Nppb* and *Npr1* was reduced in Lcn-2 knockout mice (**Fig. 5c**), indicating that Lcn-2 positively regulates the Nppb–Npr1 axis and NPR1 signalling."

3) Lacks a direct demonstration of the functional crosstalk between keratinocytes and sensory neurons. While knockout models show that deleting TBX20 in either cell type reduces itch, the study does not provide direct evidence (e.g., through co-culture experiments) to show how one cell type's actions influence the other. At least, some functional assessment of BNP-NPR1 in keratinocytes or in sensory neurons would be helpful.

Response: Thank you for this important comment. To address this, we isolated and cultured keratinocytes from epidermis of TBX20$^{fl/fl}$;K14-Cre and TBX20$^{fl/fl}$ mice, and stimulated them with BNP for 24 hours. This condition has been shown to induce IL-10, MMP3, VEGF, CXCL9, IGFBP-3, and LDLR in TBX20$^{fl/fl}$ keratinocytes, while reducing their expression in TBX20$^{fl/fl}$;K14-Cre keratinocytes (**Fig. 3c**). The conditioned media from these cultures were then applied to cultured trigeminal ganglion neurons (TGNs) isolated from TBX20$^{fl/fl}$ mice, and neuronal calcium influx was assessed using calcium imaging.

As shown in **Supplementary Fig. 5**, conditioned media from TBX20$^{fl/fl}$;K14-Cre keratinocytes elicited a significantly lower peak of calcium response in TGNs compared with conditioned media from TBX20$^{fl/fl}$ keratinocytes. Additionally the number of responding neurons were decreased. These results indicate that loss of TBX20 in keratinocytes reduces their ability to activate sensory neurons.

Accordingly, we have revised our manuscript as follows. In the main text, the revisions are highlighted in yellow.

(P25L678-687) "To examine keratinocytes-sensory neuron crosstalk, keratinocytes isolated from TBX20$^{fl/fl}$;K14-Cre and TBX20$^{fl/fl}$ mice were stimulated with BNP for 24 hours, and the resulting conditioned media were applied to TGNs isolated from TBX20$^{fl/fl}$ mice. Calcium imaging revealed that conditioned media from BNP-stimulated TBX20$^{fl/fl}$;K14-Cre keratinocytes elicited significantly reduced peak of neuronal calcium responses compared with media from TBX20$^{fl/fl}$ keratinocytes (**Supplementary Fig. 5a-c**). Because neurons were not directly exposed to BNP and conditioned media were generated under identical stimulation conditions, these differences likely reflect TBX20-dependent changes in keratinocyte-derived soluble factors, supporting a paracrine mechanism by which keratinocytes enhance sensory neuron excitability."

4) Technical and Data Presentation issues undermine the reliability of the findings, such as the skin IHC provided in Figure 1. Others are of insufficient quality, and the lack of high-resolution confocal images makes it difficult to verify key localizations.

The staining for PGP9.5 and NPR1 around the edges appears to be an artifact rather than the expected staining, which raises questions about the technique or antibody specificity. The antibody specificity for NPR1 in TG (Trigeminal Ganglion) tissue is a significant concern. The authors should have performed in situ hybridization (ISH) to provide independent confirmation of NPR1 expression. Additionally, they should have co-stained for known itch markers in the DRG to identify the specific neuronal population expressing NPR1 more accurately.

Response: We thank the reviewer for their thoughtful comments. To address the issue about the lack of high-resolution confocal images, we repeated the human skin staining using high-resolution confocal microscopy and co-stained TBX20 with K14 to clearly identify basal keratinocytes. The new images revealed robust upregulation of TBX20 in keratinocytes from lesional AD skin compared with healthy controls. Notably, TBX20 is predominantly localized in basal keratinocytes labelled by the K14 antibody, with some showing perinuclear and nuclear localization. These results have been used in current **Fig. 1a** to replace the previous images.

Accordingly, we have revised our manuscript as follows. In the main text, the revisions are highlighted in yellow.

(P21L540-544) "Confocal imaging revealed markedly elevated TBX20 protein expression in the basal K14^{+}-keratinocytes of lesional AD (LAD) skin compared with healthy controls (HC), with partial nuclear localization (**Fig. 1a**). Quantification showed a 2-fold increase in TBX20 signal in randomly selected K14^{+} keratinocytes (**Fig. 1b**)."

To address the reviewer's concern regarding NPR1 antibody specificity, unfortunately we could not validate the antibody specificity due to lack of knockout mice or tissue. To address this concern, we performed RNAscope on skin sections of human LAD vs HC. The specific probes detected a clear NPR1 mRNA expression in LAD skin vs. HC, and the levels are increased in LAD skin; additionally their cooccurrence with TBX20 mRNA in keratinocytes were increased. These results are now included in the revised **Fig. 1c**.

Accordingly, we have revised our manuscript as follows. In the main text, the revisions are highlighted in yellow.

(P21L544-550) "RNAscope detected *TBX20* and *NPR1* transcripts predominantly in the epidermis of both LAD and HC samples (**Fig. 1c**). Assay quality was validated using positive and negative control probes (**Supplementary Fig. 1**). Quantitative analysis revealed that the numbers of puncta for both *TBX20* and *NPR1* transcripts were elevated in LAD epidermis compared with HC (**Fig. 1d, top**). Additionally, keratinocytes co-expressing *TBX20* and *NPR1* transcripts were also increased (**Fig. 1d, bottom**), suggesting coordinated epidermal upregulation of these genes in LAD"

To address concerns regarding the ISH localisation of NPR1 in sensory ganglia, we performed RNAscope analysis. In response to the question of NPR1 colocalization with an itch-related neuropeptide, we focused on trigeminal ganglia (TG) rather than DRG, on the transgenic mice models, as the TG is anatomically and functionally

relevant to our cheek model. We selected Nppb as the itch-related neuropeptide for co-labelling using RNAscope based on its relevance to the mechanistic framework of our study.

Ipsilateral TG sections were obtained from $TBX20^{fl/fl}$;Avil-Cre and $TBX20^{fl/fl}$ mice following MC903-treatment of the cheek skin. RNAscope analysis demonstrated that neurons expressing *Nppb* transcripts were reduced, and there is significant reduction in the number of puncta of *Npr1* in *Nppb*$^{+}$ neurons, further supporting the involvement of TBX20 in regulating itch-associated neuronal populations (**Fig. 9c).**

Accordingly, we have revised our manuscript as follows. In the main text, the revisions are highlighted in yellow.

(P30L847-854) "To examine whether TBX20 regulates *Npr1* and *Nppb* expression in neurons, we performed RNAscope on ipsilateral TG from MC903-treated mice, with assay quality validated using positive and negative control probes (**Supplementary Fig. 8**). In $TBX20^{fl/fl}$ controls, a higher number of *Nppb*$^{+}$ cells was observed, whereas this number was significantly reduced in $TBX20^{fl/fl}$;Avil-Cre mice (**Fig. 9c**). *Npr1* transcripts were detected in *Nppb*$^{+}$ neurons, and the number of puncta in these neurons was significantly decreased in $TBX20^{fl/fl}$;Avil-Cre mice compared with $TBX20^{fl/fl}$ mice (**Fig. 9c**). These results suggest that TBX20 in our model positively regulates itch-related gene expression in sensory neurons."

All corresponding data from NPR1 RNAscope have been incorporated into the revised manuscript and all the immunohistochemistry staining using NPR1 antibodies have been removed from the revision.

Additionally, we performed RNAscope on human DRG sections using *NPR1* and *TBX20* probes, with assay quality validated using positive and negative control probes (**Supplementary Fig. 7**). This analysis revealed co-occurrence of NPR1 and TBX20 in a subpopulation of the cells in human DRG section (**Supplementary Fig. 7a**).

Accordingly, we have revised our manuscript as follows. In the main text, the revisions are highlighted in yellow.

(P30L828-834) "In paraffin-embedded human dorsal root ganglion (DRG) sections, RNAscope revealed both distinct and overlapping transcription of *TBX20* and *NPR1* (**Supplementary Fig. 7a**), prompting further investigation of TBX20 function in sensory neurons. *NPR1*$^{+}$-only cells accounted for approximately 10% of total cells, while *NPR1*$^{+}$*TBX20*$^{+}$ co-expressing cells represented an additional ~10% of total cells (**Supplementary Fig. 7a**). The quality of the RNAscope assay was validated using positive and negative control probes (**Supplementary Fig. 7b**)."

5) AD is a model of type 2 inflammatory itch, yet it fails to show an upregulation of several key itch cytokines known to be involved in AD, such as TSLP, IL-4, IL-13, and periostin. Lastly, the authors' assumption that BNP's effect works directly through sensory neurons is speculative, as the possibility of an indirect mechanism remains unaddressed.

Response: We appreciate the reviewer's comments. In our MC903-induced cheek model (4 nM in 20µL, WT mice), our RNAseq of the cheek skin did observe upregulation of the key type 2- associated cytokines highlighted by the reviewer. At day 7, transcripts for *Tslp*, *Il4*, *Il13*, and *Periostin* are increased in MC903-treated skin compared with vehicle controls (*Tslp*: Log2FC = 2.60, Q value=2.35E-12; *Il4*: Log2FC = 6.13, Q value= 1.08E-14; *Il13*: Log2FC = 2.54, Q value= 1.72E-06; *Periostin*: Log2FC = 0.80, Q value= 0.002). Please refer to our deposited dataset (GEO accession: GSE230812) Token: sdczwkiilpuzvwj

Furthermore, several independent studies (https://doi.org/10.1073/pnas.2100749118; DOI: 10.1538/expanim.24-0088; DOI: 10.1016/j.jid.2018.06.168; DOI: 10.1016/j.jaci.2022.11.009) consistently report that MC903 treatment leads to sustained elevation of TSLP, IL-4, IL-13, and IL-33 after MC903 treatment within the first two weeks and throughout prolonged treatment. Thus, the MC903 model reliably recapitulates the canonical type 2 inflammatory signature of AD.

To further strengthen our conclusions, we performed additional RT-qPCR experiments. In addition to the downregulation of *Il4* in skin of MC903-treated $TBX20^{fl/fl}$;K14-Cre mice compared to $TBX20^{fl/fl}$;K14 mice (**Fig. 6e**), we observed reduced *Il13* and *Periostin* transcription in skin of these knockout mice (**Supplementary Fig. 6b**). This suggests that Tbx20 selectively regulates a subset of type 2 inflammatory pathways in MC903 model.

Accordingly, we have revised our manuscript as follows. In the main text, the revisions are highlighted in yellow.

(P28L758-761) "Expression of *Mcpt8*, *Il4*, *Il19* (**Fig. 6e**) as well as *Il13* and *Periostin* (**Supplementary Fig. 6b)** was also reduced. Although *Lcn2, Tslp, and Il31* expression trended downward (**Fig. 6e** and **Supplementary Fig. 6b**), this change did not reach statistical significance."

Regarding the reviewer's final point about the direct and indirect activation of sensory neurons by BNP, we performed calcium imaging on cultured trigeminal ganglionic neurons (TGs) after BNP stimulation. We found that BNP (1µM) did activate calcium transients in a proportion of these neurons, although some response were delayed (**Supplementary Fig. 4a**), suggesting that BNP may act directly on sensory neurons while indirect pathways cannot be excluded.

Accordingly, we have revised our manuscript as follows. In the main text, the revisions are highlighted in yellow.

(P25L655-660) "To determine whether keratinocyte-specific TBX20 deletion impacts acute itch in vivo, we intradermally (*i.d.*) injected BNP (10 µg) into the mice cheek, a method known to elicit itch-related scratching behaviour [69], likely through direct activation of sensory neurons. Consistent with this mechanism, cultured trigeminal ganglionic neurons (TGNs) exhibited rapid calcium responses to BNP (1µM), with a subset displaying delayed activation (**Supplementary Fig. 4a**)"

Notably, previous studies have shown that BNP can act on sensory neurons to promote axon outgrowth and branching (https://www.pnas.org/doi/10.1073/pnas.0906880106). In addition, an study using ISH (doi: 10.1186/1744-8069-10-4), together with our current RNAscope findings, confirms that *NPR1* is present in sensory neurons, therefore BNP may directly act on sensory neurons.

We agree that additional experiments are needed to fully distinguish between direct and indirect mechanisms, which would require an extensive study using sensory neuron-specific NPR knockout experiments in future research.

We deeply appreciate the time and effort the Reviewer took in reviewing our manuscript.

COMMENTS FROM REVIEWER #2
Reviewer #2 (Remarks to the Author):

The paper presents novel insights into chronic itch conditions by investigating the role of BNP-NPR1 axis in keratinocytes and sensory neurons. The authors conducted cell-based assays, analysis of clinical samples along with generation of transcription factor-deficient mice to study the role of TBX20 in keratinocytes and sensory neurons in AD and ACD mouse models. Based on the results, the authors suggest that TBX20 plays an important role in itch regulation and modulates immune response and skin barrier integrity.

However, there are certain aspects that need more specific explanation and clarification.

Response: We greatly appreciate the reviewer's favourable comments and valuable suggestions to improve our manuscript. According to the Reviewers' suggestions, we performed additional experiments and revised the manuscript.

Major comments:
1). When conducting behavioral experiments, what was the reason of using male LCN-2 KO mice, and not female ones? As the behavioral patterns of male and female mice may vary, the mice of the same sex would give more consistent results. Additionally, through the whole paper there was no behavioral data for WT mice treated with PBS and MC903/DNFB. I would suggest to add this data to see the difference in behavioral phenotype between WT and transgenic mice.

Response: Thank you for raising this important question. We initially conducted the behavior experiments on the female mice in our LCN-2 KO vs. WT experiments using both the MC903 models. However, we did not observe significant differences in scratching behavior between the genotypes (please see the figure provided below):

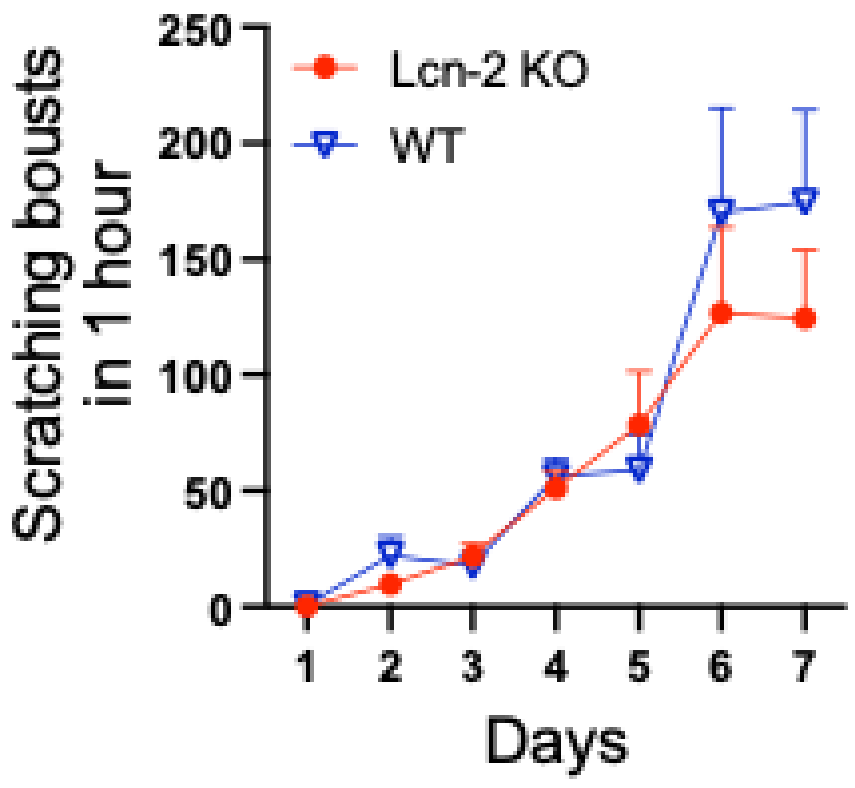

It has been reported that Lcn2 deficiency in female mice can influence estradiol biosynthesis and estrogen receptor signaling, which may in turn affect brain function and behavior, such as anxiety, stress responses, and cognition (doi: 10.1210/en.2011-1642). Although this is an interesting and relevant topic, it falls outside the scope of our current study.

Accordingly, we updated our text to discuss this possibility. In the main text, the revisions are highlighted in yellow.

(P33L925-930) "However, we did not observe significant differences in scratching behaviour between the genotypes in female mice. It has been reported that Lcn2 deficiency in female mice can influence estradiol biosynthesis and estrogen receptor signalling, which may in turn affect brain function and behaviour—such as anxiety, stress responses, and cognition[14]. Although this is an interesting and relevant topic, it falls outside the scope of our current study."

In response to the reviewer's concern about the model of WT mice, we now have included the behavioural data for vehicles- and MC903/DNFB-treated WT mice in the revised manuscript (incorporated with Lcn2-KO mice in **Fig.5a** and **5e**) to provide a complete comparison.

Accordingly, we have revised our manuscript as follows. In the main text, the revisions are highlighted in yellow.

(P27L721-723) "Consistent with these findings, Lcn-2 knockout significantly reduced MC903-induced ear scratching compared with WT mice, without affecting the minimal scratching observed in vehicle-treated controls (**Fig. 5a**)."

And,

(P27L728-729) "Similarly, Lcn-2 knockout significantly suppressed DNFB-induced itch compared with WT mice, without affecting scratching in vehicle-treated controls (**Fig. 5e**)."

2). For 'Animal Models and Itch Behavior Scoring' section – please provide the references for all models. Additionally, for chronic itch models (MC903 and DNFB) – it is stated that 'immediately scratching behaviors were recorded for 60 min'. Does it mean that the experiments were conducted immediately after the application of MC903 and DNFB? Given that the authors aimed at investigating the chronic itch, have you tried to check the itch phenotype 24h-post exposure or before daily treatment?

Response: Thank you for bringing this issue to our attention. We have now included references for 'Animal Models and Itch Behaviour Scoring' section for all models.

Please refer to **P10L248**, **258**, and **P11L275** for the following updated references. In the main text, the revisions are highlighted in yellow.

"BNP cheek injection. … The following day, 10 μg/10 μL of recombinant BNP (catalogue number 011-23, Phoenix Biotech, Kanpur, India) in physiological saline solution supplemented with 0.1% w/v BSA was injected intradermally (i.d) into the left cheek, with the vehicle (physiological saline solution with 0.1% w/v BSA) injected into the right cheek, as previously established [69].

AD mouse model induced by MC903 in TBX20 transgenic mice. … On day 1, each mouse was applied with MC903 (4 nmol/20 µL) to either the dorsal surface of the left ear, with ethanol (vehicle) applied to the dorsal surface of the right ear for $TBX20^{fl/fl}$;K14-Cre mice for 8 consecutive days to fully induce chronic dermatitis phenotype, as previously established [21].

Allergic Contact Dermatitis (ACD) induced by DNFB in TBX20 transgenic mice. … After a four-day interval, on day 5, 10 µL of 0.3% DNFB (dissolved in a 4:1 mixture of acetone:oil) was challenged to the dorsal surface of the left ear to initiate dermatitis and itch behaviour, while 10 µL of vehicle (4:1 acetone:oil) was applied to the dorsal surface of right ear as a control, as previously established [61]."

Thank you for the thoughtful comment about the time chosen for scratching behavior recording. To clarify, the 60-min behavioral recordings were conducted immediately after the daily application of MC903 or DNFB. This was done to capture the acute scratching response associated with each treatment session.

In response to the reviewer's comments, we conducted additional behavioral recordings 24 hours post-exposure, at one hour prior each daily treatment. These new results are included in the revised **Fig. 5b** and **5f**. We found that scratching behavior at the 24-hour time point (one hour prior to daily treatment) are minimal in both LCN-2 KO mice and WT mice. This observation supports our focus on stimulus-evoked itch responses during chronic sensitization in this study, rather than baseline spontaneous itch.

Accordingly, we have revised our manuscript as follows. In the main text, the revisions are highlighted in yellow.

(P27L723-724) "Baseline scratching monitored one hour prior to MC903 application also remained unaffected (**Fig. 5b**)."

And,

(P27L729-730) "Baseline scratching monitored one hour prior to DNFB application remained minimal (**Fig. 5f**)."

3). For the behavioral experiments, the duration is not consistent which makes it hard to interpret the data. Could the authors clarify how the duration was defined?

Response: We appreciate the reviewer's comment. The duration of behavioural recordings was defined based on the kinetics and selected to capture the peak itch responses characteristic of each model.

For acute itch assays, scratching behaviour was recorded for 30 minutes following BNP injection and 60 minutes following Lcn-2 injection, consistent with standard time window used to capture peak acute itch response. As shown in **Fig. 3d, left panel**, BNP-induced cheek scratching declined to the baseline within 30 mins (**Fig. 3d, right panel**), whereas in **Fig. 4e**, Lcn-2-induced cheek scratching returned to baseline within 60 mins, therefore, scratching bouts were quantified over 30 mins for BNP and over 60 mins for Lcn-2 (**Fig. 4d)**.

For the chronic models (MC903 and DNFB), scratching behaviour was recorded for 60 minutes following each daily application, consistent with prior studies using these paradigms. For the MC903 model, dermatitis was established over a minimum of 7 days to ensure full development of the chronic phenotype (chronic eczematous dermatitis associated with dry scaly skin or xerosis) before behavioural analysis and inflammatory response measurement (Brian S Kim et al . Sci Transl Med. 2013 Jan 30;5(170):170ra16). For the DNFB-induced ACD model, mice were first sensitized by DNFB application to the abdominal skin to induce the contact hypersensitivity, followed by daily DNFB challenge to the ear beginning on day 5 and continuing for 5-7 days to ensure robust induction of ear swelling and dermatitis (Takahiro Nagatake et al. Journal of Allergy and Clinical immunology. 2018 142(2):470-484).

4). For Figure 1F, the authors aimed at assessing the co-localization of TBX20 and NPR1-expressing keratinocytes (KC), however, the KC marker was not used (e.g. Krt14). Additionally, how many KC co-express TBX20 and NPR1? The quantification would support your data. Also, how many sample were used?

Response: Thank you for these comments. Regarding the assessment of TBX20 and NPR1 co-localization in keratinocytes, we addressed this concern using two complementary approaches. We also note that the use of RNAscope to validate co-localization was independently suggested by other reviewers, and we therefore incorporated this approach in the revised manuscript.

First, to clearly establish the keratinocyte identity of TBX20-expressing cells, we performed co-immunofluorescence staining for TBX20 together with KRT14 (K14), a specific basal keratinocyte marker, in human lesional AD skin and healthy control skin. Confocal microscopy demonstrated that in lesional AD skin, TBX20 signal was predominantly localized within $K14^+$ basal keratinocytes. While K14 showed the expected cytoplasmic distribution, some $TBX20^+$ cells exhibited a perinuclear/nuclear localization pattern of TBX20, consistent with its role as a transcription factor. These updated images have replaced the original images in **Fig. 1a**.

Importantly, the specificity of the TBX20 antibody was validated using skin tissue from $TBX20^{fl/fl}$;K14-Cre mice (**Fig. 3b, bottom panels**) and TG tissue from $TBX20^{fl/fl}$;Avil-Cre mice (**Fig. 9a, bottom panels**), in which TBX20 signal was absent compared with $TBX20^{fl/fl}$ control mice, confirming antibody specificity.

Second, because we were unable to validate the specificity of the NPR1 antibody due to the lack of appropriate knockout tissue, and in response to reviewers' suggestions, we performed RNAscope *in situ* hybridization for *TBX20* mRNA and *NPR1* mRNA to assess cellular identity and spatial co-localization at the transcript level (current **Fig. 1c**). This RNA-based approach avoids concerns regarding antibody specificity and enables single-cell resolution analysis. RNAscope revealed a significantly increased number of *TBX20*$^+$ and *NPR1*$^+$ cells in lesional AD skin compared with healthy controls. Importantly, a substantial proportion of *TBX20*$^+$ keratinocytes co-expressed *NPR1* transcripts and these co-expression level is higher in LAD than HC (**Fig. 1d**).

For all staining experiments, tissues from at least three independent human donors (or three animals per group for mouse studies) were analysed. Quantification was performed from multiple randomly selected fields per sample.

Accordingly, we have revised our manuscript as follows. In the main text, the revisions are highlighted in yellow.

(P21L540-550) "Confocal imaging revealed markedly elevated TBX20 protein expression in the basal K14^{+}-keratinocytes of lesional AD (LAD) skin compared with healthy controls (HC), with partial nuclear localization (**Fig. 1a**). Quantification showed a 2-fold increase in TBX20 signal in randomly selected K14^{+} keratinocytes (**Fig. 1b**). RNAscope detected *TBX20* and *NPR1* transcripts predominantly in the epidermis of both LAD and HC samples (**Fig. 1c**). Assay quality was validated using positive and negative control probes (**Supplementary Fig. 1**). Quantitative analysis revealed that the numbers of puncta for both *TBX20* and *NPR1* transcripts were elevated in LAD epidermis compared with HC (**Fig. 1d, top**). Additionally, keratinocytes co-expressing *TBX20* and *NPR1* transcripts were also increased (**Fig. 1d, bottom**), suggesting coordinated epidermal upregulation of these genes in LAD."

5). For Figure 3C and 3E, please provide the references validating the marker panel used. How were these markers selected? Also, in AD context it is known that KC release important itch-related cytokines and chemokines such as IL-33, IL-25, TSLP, IL-1, CCL5, CCL27 etc. Have the authors tried to analyze them? Additionally, for Figure 3E please revise the groups: according to the text, the groups are 'TBX20fl/fl + BNP', and 'TBX20fl/fl; K14-cre + BNP', otherwise it is confusing.

Response: Thank you for the comments. As suggested, we have now included the references for the markers selected. In the main text, the revisions are highlighted in yellow.

(P24L645-651) "In TBX20$^{fl/fl}$ keratinocytes, BNP treatment upregulated multiple inflammatory and itch-associated mediators, including IL-10 (T$_{H}$2 immunity and eosinophilic inflammation in allergic dermatitis[20]), MMP-3 (elevated in AD and promotes barrier disruption and inflammation[5]), VEGF (vascular endothelial growth factor driving vascular changes in AD lesions [42]), CXCL9 (T-cell recruitment and associated with chronic AD lesions [54]), IGFBP-3 (insulin-like growth factor-binding protein 3, associated with allergic disease[56]), and LDLR (low-density lipoprotein receptor regulating lipid metabolism and inflammatory homeostasis[11]) (**Fig. 3c**)."

Moreover, these were presented because they are encompassed within the cytokine antibody array assay scope defined by the company that was used in our study.

To address the question about alterations of itch-related cytokines and chemokines, we reanalysed our RNAseq data for transcriptions of *Il33, Il25, Tslp, Ccl5, Ccl27, Il1α* and *Il1β*. Among these cytokines, the FPKM values for *Il1α* and *Il1β* were reanalysed. The original data is deposited on https://www.ncbi.nlm.nih.gov/geo/query/acc.cgi?acc=GSE281758 and enter the token: adsreiewldqrpsd. Please refer to the current **Supplementary Fig. 4b**.

Accordingly, we have revised our manuscript as follows. In the main text, the revisions are highlighted in yellow.

(P25L669-675) "In addition, several itch and inflammation-associated genes, including *Osm*, *Lcn2*, *Il4*, *Mcpt8*, *Il17a*, *Il19*, *Il20*, *Il24*, *Il27*, also significantly downregulated in knockout mice (**Fig. 3e**). Additionally, RNAseq analysis indicated reduced *Il1α* and *Il1β* expression by FPKM analysis but not by count-based analysis, *whereas Il33*, *Il25*, *Tslp (Thymic Stromal Lymphopoietin)*, *Ccl5*, *Ccl27a* and *Ccl27b* were unchanged based on both count-based analysis and FPKM comparisons (**Supplementary Fig. 4b**)."

We also appreciate the reviewer's careful attention. We have corrected the group labels in revised **Fig. 3e** to 'TBX20$^{fl/fl}$ + BNP', and 'TBX20$^{fl/fl}$; K14-cre + BNP'.

6). Could the authors clarify the rationale for focusing on macrophages and basophils in Figures 4D? Were other relevant populations (e.g., TH2, TH17, TH1, Treg, dendritic cells, mast cells, neutrophils) analyzed, and if so, what were the results? If not, please justify their exclusion. Additionally, please justify how the markers were defined in Figure 4E. Have the authors tried to analyze IL-13 and IL-31 (which are also critical mediator of itch)?

Response: We thank the reviewer for this insightful comment. In the revised version, previous **Fig. 4d** is current **Fig. 6a**. In the MC903 model, we focused on macrophages and basophils because these two populations showed the most prominent transcriptional and functional changes in our preliminary analyses and are known to play key roles in MC903-induced dermatitis. Macrophages are a major inflammatory population in this model, and basophils are important sources of type 2 cytokines and pruritogenic mediators, making them directly relevant to our mechanistic questions. In the MC903-induced dermatitis model, CD68, CD11b, and MCPT8 were prioritized as markers because they capture key pathogenic immune populations. CD68 marks macrophages, which contribute to cytokine production and tissue inflammation. CD11b identifies a broad range of myeloid cells, including monocytes and neutrophils, reflecting the innate immune infiltration. MCPT8 specifically labels basophils, which are central drivers of MC903-induced skin inflammation. We have edit the text to clarify this reason.

Accordingly, we have revised our manuscript as follows. In the main text, the revisions are highlighted in yellow.

(P27L747-751) "These markers were prioritized to capture key pathogenic immune populations. CD68 labels macrophages that contribute to cytokine production and tissue inflammation; CD11b identifies a broad range of myeloid cells, including monocytes and neutrophils, reflecting the innate immune infiltration; and MCPT8 specifically labels basophils, which are central drivers of MC903-induced skin inflammation[67]"

While other subsets such as T_H1, T_H2, T_H17, and Tregs are also relevant, their accurate identification requires more complex intracellular or multi-parameter approaches. However, T_H1, T_H2, T_H17 are functional subsets within the $CD4^+$ T-cell population, staining for CD4 is essential to identify and analyze these subsets.

Additionally, most classical regulatory T cells (Tregs) are $CD4^+$. Thus, we performed the immunofluorescence staining using a CD4 antibody. This analysis revealed a downregulation of $CD4^+$ cells in MC903-induced skin of $TBX20^{fl/fl}$;K14-Cre mice compared with $TBX20^{fl/fl}$ controls (**Supplementary Fig. 6a**).

Accordingly, we have revised our manuscript as follows. In the main text, the revisions are highlighted in yellow.

(P28L752-755) "In addition, infiltration of CD4+ T cells was also decreased (**Supplementary Fig. 6a**). Because T_H1, T_H2, and T_H17 cells are functional subsets within the $CD4^+$ T-cell compartment, and most classical regulatory T cells (Tregs) are also $CD4^+$, CD4 serves as a foundational marker for assessing these populations[68]"

Considering that neutrophils can be partially captured using the pan-myeloid marker CD11b, which was already included in our study, and given that neutrophils primarily contribute during the early/acute stage of atopic dermatitis, we did not perform additional experiments specifically identifying neutrophils.

In response to the reviewer's question about biomarkers selection, the reason that we analysed *Npr1*, *Mcpt8*, *Lcn2*, *Il4*, and *Il19* is because that these transcripts were downregulated in BNP-induced itch model in $TBX20^{fl/fl}$;K14-Cre mice compared with $TBX20^{fl/fl}$ controls (**Fig. 3e**). To determine whether a similar pattern occurs in MC903-induced itch, we performed the same analysis in the MC903 model and found that these genes were similarly downregulated, whereas Lcn-2 exhibited only a downward trend (**Fig. 6e**).

Regarding IL-13 and IL-31, in response to the reviewer's suggestion, we performed additional RT-qPCR analysis to assess their transcription levels in the skin. We found that *Il13* transcription level was significantly reduced in the MC903-treated skin from $TBX20^{fl/fl}$;K14-Cre mice compared with $TBX20^{fl/fl}$ controls, whereas *Il31* transcription showed a declining trend but did not reach statistical significance. In the revised version, we also analysed *Periostin* and *Tslp*, and observed that *Periostin* transcription was significantly reduced in $TBX20^{fl/fl}$;K14-Cre mice. These newly analyzed markers were presented in **Supplementary Fig. 6b**.

Accordingly, we have revised our manuscript as follows. In the main text, the revisions are highlighted in yellow.

(P28L758-761) "Expression of *Mcpt8*, *Il4*, *Il19* (**Fig. 6e**) as well as *Il13* and *Periostin* (**Supplementary Fig. 6b**) was also reduced. Although *Lcn2, Tslp, and Il31* expression trended downward (**Fig. 6e** and **Supplementary Fig. 6b**), this change did not reach statistical significance".

7). For Figure 5D – please provide the references for the marker panel. How were these markers selected?

Response: Thank you for this comment. In response, we have added references supporting the selection of these genes in the revised version. These genes were

selected based on their function as key mediators of type 2 inflammation, neuroimmune signaling, and microbial sensing pathways implicated in AD.

Please refer to the updated references in the text corresponding to previous **Fig. 5d** (current **Fig. 9d**):

(P31L857-862) "These included Il13ra1 and Il13ra2, key receptors mediating IL-13–driven inflammation in AD[59]; *Nppb*, a critical neuroimmune mediator of itch and inflammation, known to be upregulated in AD lesion [30]; *Tlr1*, a microbial sensor that contributes to skin inflammation responses and is upregulated in lesions with high house dust mite sensitization[17]; and *Il31ra*, which mediates IL-31-driven itch and type 2 inflammation[30] (**Fig. 9d**)."

8). For Figure S1A, the KC marker was not used to support the statement that 'Lcn-2 was specifically increased in epidermal KC'.

Response: Thank you for this comment. In response, we performed co-staining of the LCN-2 antibody with the basal keratinocyte marker K14 on skin sections from human lesional AD and healthy controls, followed by confocal imaging. This new result showed that LCN-2 predominantly localized in $K14^+$-keratinocytes. In the revised manuscript, we have replaced the original **Supplementary Fig. 1a** (current **Fig. 4a**) with the new representative confocal images.

Accordingly, we have revised our manuscript as follows. In the main text, the revisions are highlighted in yellow.

(P26L693-695) "Supporting this hypothesis, immunohistochemical co-staining confocal images revealed a pronounced increase in Lcn-2 expression in basal $K14^+$-keratinocytes of human LAD skin compared with HC (**Fig. 4a**)."

9). In line 553, it was stated that knock-out of TBX20 in KC led to a significant downregulation of Lcn-2 transcription compared to other genes. Please provide statistical results for the other genes to support this claim. In Supplementary Fig. S1B, Saa3 and Ms4a4c also appear decreased—do these changes reach statistical significance? Additionally, have the authors tried to analyze Lcn-2 target cells?

Response: We are grateful for the valuable suggestions and have now analysed the statistical significance by count-based analysis and by FPKM values, incorporating the results into the manuscript. Specifically, *Hp*, *Sprr2a3*, *Clec4e*, *Ms4a4c*, *Saa2*, and *Serpina3m* were significantly downregulated based on both the count-based analysis and FPKM in the skin of BNP-injected $TBX20^{fl/fl}$;K14-Cre mice compared with $TBX20^{fl/fl}$ controls.

Please refer to the current **Fig. 4c** and current **Supplementary Fig. 4b** for these results.

Accordingly, we have revised our manuscript as follows. In the main text, the revisions are highlighted in yellow.

(P26L695-705) "In BNP-injected mouse cheek skin, keratinocyte-specific deletion of TBX20 significantly downregulated *Lcn-2* (Log2fc = -1.6; q-value =0.0028) (**Fig. 4b**). Several inflammatory and stress-associated genes were also identified by count-based analysis, including *Hp* (log□FC = −1.2; q = 0.0008), *Sprr2a3* (log□FC = −1.8; q = 0.006), *Clec4e* (log□FC = −1.7; q = 0.02), *Ms4a4c* (log□FC = −1.6; q = 0.01), *Saa2* (log□FC = −4.4; q = 0.0004), and *Serpina3m* (log□FC = −1.4; q = 0.01). *Saa3* (log□FC = −2.58; q = 0.006) and *GM21104* (log□FC = −21.73; q = 2.65 × 10^{-6}) were also decreased based on count-based analysis; however, their corresponding FPKM values did not show statistically significant differences (**Fig. 4c** and **Supplementary Fig. 4b**). In contrast, *Saa1* (log□FC = −2.67; q = 0.07) was not significantly altered in the count-based analysis, although FPKM values indicated significant downregulation."

In response to reviewer's comment about Lcn-2 target cells, we analysed the effect of LCN-2 on murine TGNs sensory neurons using live-cell calcium imaging analysis and observed that a small fraction of neurons responded to LCN-2 stimulation (1 µg/ml) (see current **Fig. 4f**). This finding is consistent with the presence of SLC22A17, one of the LCN-2 receptors, in sensory neurons (https://journals.lww.com/itch/fulltext/2024/04010/possible_involvement_of_satellite_glial.2.aspx)

Accordingly, we have revised our manuscript as follows. In the main text, the revisions are highlighted in yellow.

(**P26L718-720**) " To determine whether Lcn-2 can directly activate sensory neurons, we applied Lcn-2 to cultured murine TGNs, which elicited calcium influx in a distinct subset of neurons (~4.74%) (**Fig. 4f**)."

10). According to 'GSE281758_core_table_gene', acute-phase protein serum amyloid genes (Saa1, Saa2, Saa3) are also significantly upregulated along with Il1b. The relationship between SAA proteins and IL-1β has already been investigated in psoriatic (IMQ-induced) skin inflammation (Yu N et al. Clin Exp Immunol. 2015). Have the authors considered to analyze this axis in relation to dysregulation of TBX20/NPR1 in AD and ACD context? Could the authors explain the preference of Lcn2 over Saa and IL-1β genes?

Response: We appreciate reviewer's insightful feedback. Our initial focus centred on chronic itch. Based on the literature, Lcn-2 has been implicated in maintaining the itch-scratch cycle in chronic itch, although its function in the peripheral nervous system remains unexplored (https://www.jacionline.org/article/S0091-6749(19)31365-X/fulltext). Serum Lcn-2 levels also correlate with itch severity in psoriasis, but this relationship has not yet been investigated in atopic dermatitis (https://onlinelibrary.wiley.com/doi/10.1155/2019/8171373). Furthermore, Lcn-2 and BNP have been shown to correlate functionally across several disease contexts, including sepsis-induced myocardial injury, where elevated Lcn-2 levels are associated with increased pro-B-type natriuretic peptide (pro-BNP) (https://doi.org/10.3389/fcvm.2022.1009726). Collectively, these findings motivated our focus on Lcn-2 in the current study.

Accordingly, we have updated the text to justify the choice of Lcn-2. In the main text, the revisions are highlighted in yellow.

(P26L710-714) "Lcn-2 has been implicated in the chronic itch-scratch cycle[19] and correlates with itch severity in psoriasis[1]; however, its role in the periphery and in AD remains poorly defined. Notably, functional interactions between Lcn-2 and BNP have been described in other pathological settings, such as sepsis-induced myocardial injury[23], further supporting the existence of a TBX20-BNP-NPR1 axis in the regulation of itch."

In response to the reviewer's question, we assessed the potential relationship of Saa1, Saa2, Saa3, and Il1b to BNP-elicited itch following TBX20 depletion in keratinocytes by analyzing their expression levels. We found that *Saa2* and *Saa3* were also significantly downregulated based on both count-based analysis (current **Fig. 4c** and **Supplementary Fig. 4b**). Although these genes are not known to directly mediate itch, we do not exclude the possibility of indirect contributions. We agree that integrating this gene into future chronic itch research will be important for advancing our understanding of downstream components of the TBX20/NPR1 axis. To reflect this, we have added a discussion of their potential involvement in the revised manuscript as follows. In the main text, the revisions are highlighted in yellow.

(P26 L705-709) "*Hp*, *Saa2*, *Saa3, Clec4e*, *Ms4a4c*, and *Sprr2a3*, and *serpina3m* have been reported to reflect innate immune activation, acute-phase inflammatory signalling, and compensatory epidermal barrier responses in skin disease; however, their roles in AD remain unclear [13,27,51,62,65]. In our dataset, these genes were downregulated, suggesting reduced innate immune and acute-phase responses."

11). For Figures S1E and S1F, please explain the difference in duration of experiments as it can affect the data interpretation. Please clarify whether the skin dissection time point was kept constant across all experiments. Additionally, does the deficiency of Lcn-2 affect Mcpt8+ population?

Response: Thank you for the comment. For the MC903 model, we dissected the skin after day 7 (original Supplementary **Fig. 1e**, current **Fig. 5a**), when the model is fully established. Because the DNFB-induced ACD model consists of two phases—sensitization and challenge, we presented only the challenge phase in the graph (original Supplementary **Fig. 1f**, current **Fig. 5e**), beginning on day 1 of the challenge, which corresponds to day 5 post-sensitization. This model becomes fully established after 5 days, at which point we performed skin dissection. Therefore, the dissection time points for both models were selected to coincide with their fully developed inflammatory states. In addition, we harvested all skin samples five hours for both models after the final recording to allow transcriptional changes to occur.

Accordingly, we have revised our manuscript as follows. In the main text, the revisions are highlighted in yellow.

(P27L732-733) "Dissection time points for both models were selected to coincide with their fully developed inflammatory states [18,21,34]".

Throughout the whole paper, MC903 model was terminated after 7-8 days of MC903 application, when the AD phenotype was established (**Fig. 5a, Fig. 6a, Fig. 9b**). The DNFB model was terminated at day 5-7 of the DNFB challenge phase, when the ACD phenotype was established (**Fig. 5e** and **Fig. 8a**).

In response to the reviewer's suggestion, we have additionally analysed the Mcpt8$^+$ population and included these results in right panels of **Fig. 5d** (for MC903) and **Fig. 5g** (for DNFB). Notably, infiltration of Mcpt8$^+$ cells was significantly reduced in Lcn-2 KO mice in the DNFB model, while the MC903 model showed a decline that did not reach statistical significance.

Accordingly, we have revised our manuscript as follows. In the main text, the revisions are highlighted in yellow.

(P27L726-732) "Lcn-2 deficiency further reduced MC903-induced infiltration of CD11b$^+$ and CD68$^+$ cells, with a nonsignificant reduction in Mcpt8$^+$ basophils (**Fig. 5d**).

Similarly, Lcn-2 knockout significantly suppressed DNFB-induced itch compared with WT mice, without affecting scratching in vehicle-treated controls (**Fig. 5e**). Baseline scratching monitored one hour prior to DNFB application remained minimal (**Fig. 5f**). In this model, Lcn-2 deficiency did not significantly reduce CD11b$^+$ and CD68$^+$ cell infiltration but significantly decreased basophils numbers (**Fig. 5g**)."

12). In Figure 7B – the graph is not clear - total scratching bouts on day 13?

Response: Thank you for bringing this to our attention. The total scratching was analysed on day 11 for comparison, as this time point showed the most significant difference (current **Fig. 7d**). To improve the clarity, we have labelled day 11 on the graph (current **Fig. 7c**).

Accordingly, we have revised our manuscript as follows. In the main text, the revisions are highlighted in yellow.

(P28L782-784) "Scratching bouts were significantly increased in the AAV-TBX20 group compared with the AAV-control group. This increase was evident on day 11 (**Fig. 7c**) and remained significantly elevated on day 13 (**Fig. 7d**)."

13). Figure 7H – the data for Mcpt8+ cells is confusing – in comparison with Figure 6A and Figure 4D, the number of Mcpt8+ cells in Figure 7H vehicle group is sharply decreased – how can it be explained? Does the genetic background of mice (WT vs TBX20fl/fl) affect the result?

Response: Thank you for the comment. This difference may be attributable to variations in genetic background and experimental procedures. Original **Fig. 7h** (current **Fig. 7i**) used WT mice to evaluate pharmacological inhibition by NRG1, in which mice received three intraperitoneal injections of either NRG1 or vehicle. In contrast, original **Fig. 6a** (current **Fig. 10a**) and original **Fig. 4d** (current **Fig. 6d**) used mice on a transgenic background (TBX20$^{fl/fl}$) that did not receive injections.

Therefore, differences in Mcpt8^{+} cell numbers may reflect both genetic background effects and the repeated injection procedures.

14). Figure 7A – have the authors tried to use AAV-mediated Tbx20 rescue in sensory neurons? If not, please explain why rescue was restricted to keratinocytes?

Response: Thank you for the comment. We did not perform AAV-mediated Tbx20 rescue experiments in sensory neurons in the current study, as achieving specific delivery to sensory neurons via local injection remains technically challenging. Efficient transduction of sensory ganglia usually requires different delivery routes (intraganglionic or relay on retrograde transport). Those routes are surgically more invasive, and can introduce confounding effects. However, we agree this is an interesting question and represents a potential direction for future work.

15). According to RT-qPCR results, IL-13 significantly upregulated Tbx20 and Npr1 transcription in NHEK highlighting the importance of interaction between IL-13 and KC – however, IL13 and IL-13-producing cells-related data is lacking in this study. I would suggest to investigate the change of this cytokine and related cells in the context of TBX20 deficiency.

Response: Thank you for this important comment. In response, we performed RT-qPCR analysis of IL-13 expression in skin samples from MC903-induced TBX20$^{fl/fl}$; K14-Cre mice and compared the results with TBX20$^{fl/fl}$ controls. As shown in **Supplementary Fig. 6b**, IL-13 expression was significantly reduced in the knockout mice. We also conducted IHC using a CD4 antibody to label CD4^{+} T helper lineage cells, a major source of type 2 cytokines including IL-13. Consistent with the RT-qPCR results, CD4^{+} cell infiltration was significantly reduced in the TBX20$^{fl/fl}$;K14-Cre mice (**Supplementary Fig. 6a**).

Accordingly, we have revised our manuscript as follows. In the main text, the revisions are highlighted in yellow.

(P28L757-761) "RT-qPCR analysis further confirmed downregulation of *Npr1* transcripts in MC903-treated ear skin of TBX20$^{fl/fl}$;K14-Cre mice. Expression of *Mcpt8*, *Il4*, *Il19* (**Fig. 6e**) as well as *Il13* and *Periostin* (**Supplementary Fig. 6b)** was also reduced. Although *Lcn2, Tslp, and Il31* expression trended downward (**Fig. 6e and Supplementary Fig. 6b**), this change did not reach statistical significance."

And,

(P28L752-755) "In addition, infiltration of CD4+ T cells was also decreased (**Supplementary Fig. 6a**). Because T_H1, T_H2, and T_H17 cells are functional subsets within the CD4^{+} T-cell compartment, and most classical regulatory T cells (Tregs) are also CD4^{+}; CD4 serves as a foundational marker for assessing these populations[68]"

16). In Discussion part, the authors did not mention basophils (Mcpt8+ cells), although the Mcpt8 data was provided across the study – please discuss the importance of basophils in the context of TBX20-NPR1 axis underlying chronic itch.

Response: As rightly suggested, and in response, we have added the following text discussing the basophil relevance in the context of TBX20-NPR1 axis in the revised manuscript as follows. In the main text, the revisions are highlighted in yellow.

(P34L953-957) "The reduction in Mcpt8^{+} cells following TBX20 knockout in keratinocytes and sensory neurons suggests that TBX20 regulates basophil recruitment to the skin. Given that basophils amplify T_H2-driven inflammation and itch through T_H2 promotion and release of IL-4, IL-13, and the pruritogen LTC_4 (Leukotriene C4), these findings link TBX20–NPR1 signalling to itch [67]."

Minor comments:
1. What serotype of AAV was used in your experiments? Please indicate it.

Response: Thank you for the comment. The serotype of AAV is AAV2/9 in our study. This is now indicated in the method and highlighted in yellow in the revision.

(P11L280) "Adeno-associated virus (AAV2/9) carrying TBX20 for overexpression"

2. For Figure 3A and B and the corresponding paragraph (lines 509-520), I suggest you to exclude the TBX20fl/fl-Avil-cre and insert it to another Figure which contains TBX20fl/fl-Avil-cre data (Figure 7) as it is confusing.

Response: Thank you for bringing this issue to our attention. As requested, we have removed TBX20$^{fl/fl-}$Avil-cre from **Fig. 3a** and **3b** and the corresponding paragraph. The relevant information has been added to the text for current **Fig. 9a**.

In the main text, the revisions are highlighted in yellow.

(P30L836-837) "To explore this link, we generated sensory neuron-specific TBX20 cKO mice (TBX20$^{fl/fl}$;Avil-Cre), by crossing TBX20$^{fl/fl}$ mice with Advillin (Avil)-Cre mice, using a strategy similar to that employed for producing TBX20$^{fl/fl}$;K14-Cre mice (c.f. **Fig. 3a** and **Fig. 9a**)."

3. Line 549 – please paraphrase the subtitle (check the grammar)

Response: Thank you for pointing out this mistake. In response, we edited it to the subtitle as follows. In the main text, the revisions are highlighted in yellow.

(P26L689-690) " LCN-2 acts downstream of TBX20-NPR1, and its knockout results in a deficit in MC903- and DNFB-induced itch response".

4. For line 559 – i.d. injection of Lcn-2 to WT mice? Please specify it.

Response: Thank you for the comment. We have now specified to the text as follows. In the main text, the revisions are highlighted in yellow.

(P26L715-718) "We next investigated whether Lcn-2 directly contributes to peripheral itch. Intradermal injection of recombinant Lcn-2 into the mouse cheek

induced a significant increase in itch-like behaviour compared with vehicle controls (**Fig. 4d**), with scratching peaking approximately 10 min post-injection (**Fig. 4e**).".

5. For Figure S1E and S1F, I would suggest to add 'MC903' and 'DNFB' accordingly for easier understanding.

Response: Thank you for this comment. This has been added on the corresponding panels (please refer to the current **Fig. 5**).

6. Line 620 – the sentence is confusing – immunostaining of skin or TG? Please revise it.

Response: Thank you for the comment. We have now revised it to the text as follows. In the main text, the revisions are highlighted in yellow.

(P30L839-840) "Immunohistochemical staining of trigeminal ganglion (TG) from MC903-treated $TBX20^{fl/fl}$ mice revealed TBX20 expression in a small subset of ipsilateral TG neurons, marked by PGP9.5".

Reviewer #2 (Remarks on code availability):

When trying to reach the code/raw data, the following message appears on SRA Run Selector: 'nothing has been found for accession'. The only file available for downloading is supplementary file in txt format. The supplementary file was reviewed and assessed accordingly.

Response: Thank you for bringing this issue to our attention. We have attempted to resolve this issue with GEO regarding access to SRA data. We received a response stating that "GEO passes the raw data for sequence submissions to SRA, and SRA does not support access to private read data. The GEO reviewer token only provides access to only the GEO metadata and processed data."
We subsequently contacted the SRA help desk (sra@ncbi.nlm.nih.gov) to request a private link to the SRA submission metadata for our associated BioProject accession (PRJNA1185658). However, we have not yet received notification of a private link that we could provide to the reviewers. Once our paper is accepted for publication, we will request that the raw data be made fully publicly available.

For evidence that we submitted our request to SRA help desk, please see below.

Case CAS-1602985-T7Y1H8- National Library of Medicine Customer Service confirmation TRACKING:000435001703710

NLM Support 详情

Thank you for contacting the U.S. National Library of Medicine (NLM). The details of your message are listed below. You can expect to hear from us within 2 to 5 business days. For additional information about NLM products and services, please visit https://support.nlm.nih.gov.

Case Information:
Case #: CAS-1602985-T7Y1H8
Summary: For our submission GEO (Accession: gse281758)
Details:
Could you please provide us a privite link in order to allow reviewers to assess our paper submission in relation to the PRJNA1185658 BioProject?
Thank you for your assistance.

翻译

We deeply appreciate the time and effort the Reviewer took in reviewing our manuscript.

COMMENTS FROM REVIEWER #3
Reviewer #3 (Remarks to the Author):

This study investigates the expression and functional role of TBX20 in lesional and non-lesional skin of atopic dermatitis (AD) patients, particularly focusing on its role in itch. While the observation is potentially interesting, the manuscript suffers from poor organization, unclear data presentation, and methodological inconsistencies, raising concerns about the quality and reliability of the conclusions. Significant improvements in organization, data interpretation, and methodological rigor are needed.

Response: We greatly appreciate the reviewer's favourable comments and valuable suggestions to improve our manuscript. According to the Reviewer's suggestions, we performed additional experiments and revised the manuscript.

Specific Comments
1). Figure 1, Antibody staining: The TBX20 antibody staining does not appear specific. As a transcription factor, TBX20 should be localized in the nucleus, but in Figure 1A it appears cytoplasmic. Antibody specificity should be tested in knockouts and controls. In situ hybridization (ISH): The ISH signal is very weak, making it difficult to interpret. Using RNAscope or another sensitive method could improve signal detection. Figure 1F: If the antibody works, why not consistently use it across all panels instead of mixing detection methods?

Response: Thank you for raising this important point. To further improve image quality in **Fig.1a**, we co-stained human skin samples with a TBX20 antibody and the basal keratinocyte marker K14 and acquired all images using confocal microscopy. Consistent with our previous observations, healthy skin exhibited minimal to low levels of TBX20 signal, whereas lesional AD skin showed robust TBX20 expression in $K14^{+}$ keratinocytes.

We thank the reviewer for the thoughtful consideration of the subcellular localization of TBX20. In our confocal imaging analysis, TBX20 did not exhibit exclusively nuclear localization. Although perinuclear and nuclear signals were observed in a subset of cells, this distribution was not uniform across all keratinocyte populations. Given dynamic regulation of transcription factors, the nuclear localization of TBX20 may reflect a context-dependent or transient activation state during disease progression, rather than constitutive nuclear residency.

To address the concern regarding antibody specificity, we co-stained a TBX20 antibody with basal keratinocyte marker K14 on skin samples from $TBX20^{fl/fl}$;K14-Cre mice and compared them with $TBX20^{fl/fl}$ controls and imaged them using confocal microscope. As shown in our **Fig. 3b, lower panels**, TBX20 immunoreactivity was absent in $K14^{+}$ keratinocytes from $TBX20^{fl/fl}$;K14-Cre mice, whereas clear TBX20 signals were detected in $TBX20^{fl/fl}$ controls. These findings confirmed the specificity of the TBX20 antibody.

Accordingly, we have revised our manuscript as follows. In the main text, the revisions are highlighted in yellow.

(P21L540-544) "Confocal imaging revealed markedly elevated TBX20 protein expression in the basal K14⁺-keratinocytes of lesional AD (LAD) skin compared with healthy controls (HC), with partial nuclear localization (**Fig. 1a**). Quantification showed a 2-fold increase in TBX20 signal in randomly selected K14⁺ keratinocytes (**Fig. 1b**)."

To address the reviewer's concern regarding weak signal of ISH experiments and mixed methodologies of ISH and IHC for **Fig. 1f**, we performed RNAscope experiments on skin sections of human LAD vs HC. The specific probes detected a clear *NPR1* mRNA expression in LAD skin vs. HC, and the levels are increased in LAD skin; additionally their cooccurrence with *TBX20* mRNA in epidermis were increased. These results are now included in the revised **Fig. 1c, d** to replace the original **Fig. 1f**.

Accordingly, we have revised our manuscript as follows. In the main text, the revisions are highlighted in yellow.

(P21L544-550) "RNAscope detected *TBX20* and *NPR1* transcripts predominantly in the epidermis of both LAD and HC samples (**Fig. 1c**). Assay quality was validated using positive and negative control probes (**Supplementary Fig. 1**). Quantitative analysis revealed that the numbers of puncta for both *TBX20* and *NPR1* transcripts were elevated in LAD epidermis compared with HC (**Fig. 1d**, **top**). Additionally, keratinocytes co-expressing *TBX20* and *NPR1* transcripts were also increased HC (**Fig. 1d**, **bottom**), suggesting coordinated epidermal upregulation of these genes in LAD."

2). Figure 2, The rationale for selecting H3K27ac-negative loci is unclear. The relationship to other histone modifications should be explained.

Response: Thank you for this comment. We analysed TBX2 binding at this site due to its proximity to NPR1 and its enrichment for active chromatin marks (H3K4me1, H3K4me2, and H3K4me3), together with moderate evolutionary conservation based on PhyloP and PhastCons scores, suggesting potential regulatory activity.

Accordingly, we have revised our manuscript as follows. In the main text, the revisions are highlighted in yellow.

(P22L571-574) "We prioritized analysis of an H3K27ac-marked regulatory region proximal to the *NPR1* locus based on its potential regulatory relevance. This region showed enrichment for enhancer- and promoter-associated histone modifications (H3K4me1, H3K4me2, and H3K4me3) and exhibited moderate evolutionary conservation based on Phylop and Phast scores."

3). To support the functional relevance of the NRP1 promoter/enhancer targeted by TBX20, additional experiments are needed, such as reporter assays with TBX20 overexpression or knockdown.

Response: Thank you for raising this important comment. To address this question, we performed Dual-Luciferase Reporter Assay.

Firstly, binding sites of *hTBX20* and *hNPR1* gene were predicted using the JASPAR database based on *hTBX20* matrix profile MA0689.1. The potential interaction sites identified on *hNPR1* promoter include 1333-1343bp, 1433-1443bp, 1625-1635bp, and 1854-1864bp. We then synthesized both wild-type (Wt) and mutant (Mt) promoter fragments, in which the Mt contained all four mutations on these identified potential interaction sites, and the dual-luciferase reporter vector system was used to assess promoter activity and promoter–transcription factor interactions. The pGL3-Basic plasmid was cloned with Wt or Mt *hNPR1* promoter fragment upstream of the firefly luciferase (Fluc) gene (current **Supplementary Fig. 2**); the pcDNA3.1 plasmid was used for *hTBX20* CDS region (NM001077653.2) overexpression; the pRL-TK plasmid was used for expressing Renilla luciferase (Rluc) as an internal control.

For luciferase assays, we co-transfected all three plasmids into the human keratinocyte cell line HaCaT and HEK293 cells. After 48 h of incubation, cells were treated with 1 μM BNP vs vehicle (PBS) for 24 hours, lysed and Firefly and Renilla luciferase activities were measured using the Dual-Luciferase® Reporter Assay System, with promoter activity evaluated as the Fluc/Rluc ratio.

Dual-luciferase assays showed that TBX20 significantly increased NPR1 promoter activity following BNP stimulation compared to vehicle treatment in both HaCaT cells and HEK293 cells, whereas mutation of the predicted binding sites abolished this increment, indicating a specific transcriptional interaction between TBX20 and the NPR1 promoter. This result has been included in the revised manuscript as current **Fig. 2c** and current **Supplementary Fig. 3a**.

Accordingly, we have revised our manuscript as follows. In the main text, the revisions are highlighted in yellow.

(P23L597-616) "To investigate whether TBX20 directly regulates *NPR1* transcription, a dual-luciferase reporter assay was performed. The human NPR1 promoter region (−2000 to +200 bp relative to the transcription start site [TSS]; NC_000001:153676688–153678887) was cloned upstream of the firefly luciferase gene in the pGL3-Basic vector to generate the wild-type reporter construct (**Supplementary Fig. 2**).

Four putative TBX20-binding sites within the NPR1 promoter were predicted using JASPAR (MA0689.1; relative score > 0.8, score > 8), located at positions 1333–1343 bp (0.94; 14.7), 1433–1443 bp (0.87; 10.5), 1625–1635 bp (0.85; 9.6), and 1854–1864 bp (0.81; 7.4). To assess the functional relevance of these sites, a mutant NPR1 promoter fragment harbouring simultaneous mutations at all four predicted TBX20-binding sites was synthesized (**Supplementary Fig. 2**) and cloned into the same reporter vector.

HaCaT cells were co-transfected with either the wild-type or mutant NPR1 promoter construct, a TBX20 overexpression plasmid (pcDNA3.1-TBX20), and the Renilla luciferase plasmid pRL-TK as an internal control. Promoter activity was determined by calculating the ratio of firefly to Renilla luciferase activity (Fluc/Rluc).

Dual-luciferase assays showed that TBX20 significantly enhanced *NPR1* promoter activity following BNP stimulation in HaCaT cells transfected with the wild-type

construct. In contrast, the synthesized mutant promoter construct abolished this activation, indicating that TBX20 regulates NPR1 transcription through these specific binding sites (**Fig. 2c**). Similar results were observed in HEK293 cells (**Supplementary Fig. 3a**)."

4). The authors show that TBX20 and NRP1 expression are correlated, but only test whether TBX20 regulates NRP1. They should also consider testing whether NRP1 regulates TBX20.

Response: We thank the reviewer for this constructive suggestion. To determine whether NPR1 regulates TBX20 expression, the human keratinocyte cell line HaCaT was treated with the NPR1 antagonist A71915 (1 µM for 24 h; dose selected based on DOI: 10.1038/s41598-018-25292-0, corresponding to the reported IC_{50} for human NPR1 [DOI: 10.1126/scitranslmed.aav5464]) or vehicle control, in the presence of BNP (1 µM). Total RNA was subsequently extracted, and TBX20 expression was quantified by RT–qPCR. This experiment demonstrated that BNP treatment significantly increased TBX20 expression, and this effect was attenuated by A71915, supporting the conclusion that BNP-mediated activation of NPR1 regulates TBX20 transcription in human keratinocytes. These data are now included in **Supplementary Fig. 3b**.

Accordingly, we have revised our manuscript as follows. In the main text, the revisions are highlighted in yellow.

(P24L620-626) "To determine whether NPR1 regulates TBX20 expression, HaCaT cells were treated with the NPR1 antagonist A71915 (1 µM for 24 h; dose selected based on previous study [12], and corresponding to the reported IC□□ for human NPR1 [45]) or vehicle control, in the presence of BNP (1 µM). TBX20 expression was quantified by RT–qPCR. BNP significantly increased TBX20 expression, and this effect was attenuated by A71915 (**Supplementary Fig. 3b**), indicating that BNP-mediated NPR1 activation regulates TBX20 transcription in human keratinocytes."

5. ChIP vs. RT-qPCR, Page 20, lines 497–502: The text suggests the authors performed ChIP-seq to identify TBX20 binding on the NRP1 promoter. However, Figure B appears to show gene expression (RT-qPCR), not ChIP-qPCR. The authors may have mixed gene expression and ChIP experiments, or there is a misunderstanding of the techniques.

Response: Thank you for bringing this issue to our attention. We apologize for the mistake in labelling the previous figure of the manuscript. We have now revised the Y-axis of **Fig. 2b** and the corresponding text. Specifically, the "*NPR1* Fold enrichment" was used to label the Y-axis and the main text has been updated as follows.

In the main text revised Method section, the revisions are highlighted in yellow.

(P20L487-488) "The purified DNA obtained after cross-link reversal was subsequently analysed by SYBR green qPCR".

6). Figure 3B: Primer locations should be clearly labeled to indicate PCR products. Figure legends (page 30, lines 808–810) are poorly written and difficult to understand. Additional experiments such as qPCR and IHC would help confirm gene deletion in conditional knockout (CKO) mice.

Response: Thank you for bringing this issue to our attention. In response to the reviewer's comments, we have labelled the PCR products in the **Fig. 3b**, and added the detailed genotyping method and primer information in the revised Method as follows.

In the main text, the revisions are highlighted in yellow.

(P9L218-231) "Genotyping of TBX20$^{fl/fl}$;K14-Cre and TBX20$^{fl/fl}$;Avil-Cre mice was performed by PCR using primer pairs specific for the *Tbx20* floxed allele and the respective *Cre* transgene. For detection of the *Tbx20* floxed allele, primers *Tbx20*-F (5′-CTTCTCTATTTCTGTGGTGCTCTG-3′) and *Tbx20*-R (5′-TACCAACATCCTGAAACACTTTTGG-3′) were used with an annealing temperature of 60 °C, generating a 223 bp band for the floxed allele and a 149 bp band for the wild-type allele, while heterozygous mice displayed both bands.

For identification of the *K14-Cre* transgene, primers *K14*-F (5′-CGATGGGAAAGTGTAGCCTGCA-3′) and *K14*-R (5′-TCCAGGTATGCTCAGAAAACGCC-3′) were used at an annealing temperature of 60 °C, producing a 450 bp *Cre*-specific amplicon.

For *Avil-Cre* genotyping, primers *Avil*-F (5′-GTGGTGGTGGCATGGTGATA-3′) and *Avil*-R (5′-GCACACAGACAGGAGCATCT-3′) were used under the same PCR conditions, generating a 327 bp Cre-specific amplicon. Combined analysis of these PCR products allowed reliable identification of TBX20$^{fl/fl}$;K14-Cre and TBX20$^{fl/fl}$;Avil-Cre mice.".

Additionally, we have revised the **Fig. 3b** and current **Fig. 9a** legend by stating the follows. In the legend, the revisions are highlighted in yellow.

(P36L1022-1028) "Genotyping of TBX20$^{fl/fl}$;K14-Cre mice was performed by PCR to detect the *Tbx20* floxed allele and the K14-Cre transgene. Amplification of the *Tbx20* locus yielded a 223 bp product for the floxed allele and a 149 bp product for the WT allele, while amplification of the K14-Cre transgene produced a 450 bp amplicon.",

(P40L1149-1152) "**a**. TBX20$^{fl/fl}$;Avil-Cre mice were genotyped (223 bp floxed, 149 bp WT; Avil-Cre 327 bp), with heterozygotes showing both the floxed and WT *Tbx20* bands. Representative immunohistochemical showing co-staining of TBX20 and PGP9.5 in trigeminal ganglia (TG) of TBX20$^{fl/fl}$;Avil-Cre mice and TBX20$^{fl/fl}$ mice."

And, in the main text,

(P24L636-638) "TBX20 floxed alleles yielded 223 bp (floxed), 149 bp (wild-type), or both (heterozygotes). The K14-Cre transgene produced a 450 bp amplicon, allowing reliable identification of TBX20$^{fl/fl}$;K14-Cre mice (**Fig. 3b**)."

In response to reviewer's comments, we have also added the representative images of IHC to confirm gene deletion in conditional knockout mice. Please refer to revised **Fig. 3b, lower panels** and **Fig. 9a, lower panels.** Accordingly, we have revised our manuscript as follows. In the main text, the revisions are highlighted in yellow.

(P24L639-640) "TBX20 ablation in these mice was subsequently confirmed by immunofluorescence staining (**Fig. 3b**)."

And,

(P30L840-842) "In contrast, TBX20 expression was absent in the TG of TBX20$^{fl/fl}$;Avil-Cre mice (**Fig. 9a**), confirming efficient depletion of TBX20 from sensory neurons."

7). LCN2 Focus, Page 21, line 549: The functional analysis of LCN2 is introduced without justification. Among multiple genes that are altered, it is unclear why the authors focus only on LCN2.

Response: Thank you for this comment. Our initial focus centred on chronic itch. Previous studies have implicated LCN-2 in maintaining the itch-scratch cycle during chronic itch; however, its role within the peripheral nervous system remains largely unexplored(https://www.jacionline.org/article/S0091-6749(19)31365-X/fulltext). Serum LCN-2 levels have also been reported to correlate with itch severity in psoriasis, although this relationship has not yet been examined in atopic dermatitis (https://onlinelibrary.wiley.com/doi/10.1155/2019/8171373). Moreover, functional associations between LCN-2 and BNP have been described across several disease contexts, including sepsis-induced myocardial injury, where elevated LCN-2 levels are associated with increased pro-B-type natriuretic peptide (pro-BNP) (https://doi.org/10.3389/fcvm.2022.1009726).

In our BNP-induced cheek itch model, LCN-2 transcription was significantly reduced in TBX20$^{fl/fl}$;K14-Cre mice (**Fig. 3e**), supporting a potential link between BNP signaling and LCN-2 regulation. Collectively, these findings motivated our focus on LCN2 in the current study.

Accordingly, we have revised our manuscript as follows. In the main text, the revisions are highlighted in yellow.

(P26L710-714) "LCN-2 has been implicated in the chronic itch-scratch cycle[19] and correlates with itch severity in psoriasis[1]; however, its role in the periphery and in AD remains poorly defined. Notably, functional interactions between LCN-2 and BNP have been described in other pathological settings, such as sepsis-induced myocardial injury[23], further supporting the existence of a TBX20-BNP-NPR1 axis in the regulation of itch."

8). AAV Experiments, Details on the AAV vector are missing. The authors should test efficiency and confirm that TBX20 is successfully overexpressed, ideally using IHC to show tissue specificity. Control data should be shown.

Response: Thank you for bringing this issue to our attention. We have added the AAV vector information as follows. In the main text, the revisions are highlighted in yellow.

(P11L281-283) "Adeno-associated virus (AAV2/9) carrying TBX20 for overexpression (pAAV-CMV-mCherry-P2A-Tbx20-3xFLAG-WPRE) and an equal amount of control virus (pAAV-CMV-mCherry-P2A-3xFLAG-WPRE), both from OBiO, Shanghai"

To address the concern of TBX20 is successfully overexpressed, we examined TBX20 expression in keratinocytes of the MC903-treated skin from $TBX20^{fl/fl}$;K14-Cre mice following AAV delivery using IHC. Cheek skin collected on day 13 showed that AAV-TBX20 group restored TBX20 expression in skin compared with the AAV-control group. This result has been included in the revised **Fig. 7b**.

Accordingly, we have revised our manuscript as follows. In the main text, the revisions are highlighted in yellow.

(P28L781-782) "Immunofluorescence staining confirmed the expression of TBX20 in cheek skin of AAV-TBX20 group (**Fig. 7b**)."

9). NRG1 Treatment, Page 24, line 661: The authors state, "we employed pharmacological inhibition of TBX20 using Neuregulin-1 (NRG1)," but there is no evidence or reference supporting that NRG1 inhibits TBX20. This claim is unsupported and requires clarification.

Response: We apologize for the earlier lack of clarity regarding reference 50. In the previous version, the citation was included without explaining its relevance to our use of NRG1. We have revised the main text to explicitly state that NRG1 has been shown to suppress Tbx20 expression in a dose-dependent manner[50], thereby clarifying the rationale for using NRG1 as a pharmacological inhibitor of TBX20. Reference 50 (Stennard F. A., et al. Development 2005. 132, 2451–2462) demonstrated that Tbx20 expression is significantly repressed by NRG1 in a dose-dependent manner.

Accordingly, we have revised our manuscript as follows. In the main text, the revisions are highlighted in yellow.

(P29L789-792) "To further investigate the role of TBX20 in AD-associated itch, we employed pharmacological inhibition of TBX20 using Neuregulin-1 (NRG1), a member of the paracrine epidermal growth factor family previously shown to suppress Tbx20 expression in a dose-dependent manner[50]."

And,

(P34L971-972) "Conversely, in WT mice, intraperitoneal injection of NRG1, a cardioactive growth factor released from endothelial cells[50], suppressed TBX20 expression, alleviated MC903-induced itch, and reduced $CD68^+$ and $CD11b^+$ cell infiltration."

10). The organization of the results, The manuscript mixes neural TBX20 knockout mice (TBX20^fl/fl;Avil-Cre) and skin-specific knockout mice (TBX20^fl/fl;K14-Cre). Figures 5 and 6: TBX20^fl/fl;Avil-Cre (sensory neurons). Figures 3, 4, 7: TBX20^fl/fl;K14-Cre (skin). This inconsistency makes it difficult to follow the experimental logic and interpret the results. Clear separation of the two models is required.

Response: We thank the reviewer for pointing out this issue. We apologise that mixing data from the two TBX20 conditional knockout models ($TBX20^{fl/fl}$;Avil-Cre and $TBX20^{fl/fl}$;K14-Cre) in the previous version may have caused confusion. To address this, we have now completely reorganized the Results section and updated the Figures' presentation so that:

All the skin-specific TBX20 knockout data ($TBX20^{fl/fl}$;K14-Cre) are presented together in **current Fig. 3, 6, 7, 8**.

All the chronic model of neuronal TBX20 knockout data ($TBX20^{fl/fl}$;Avil-Cre) are presented together in **current Fig 9 and 10**.

We appreciate the time and effort the Reviewer took in reviewing our manuscript.

COMMENTS FROM REVIEWER #4

Reviewer #4 (Remarks to the Author):

I co-reviewed this manuscript with one of the reviewers who provided the listed reports. This is part of the Nature Communications initiative to facilitate training in peer review and to provide appropriate recognition for Early Career Researchers who co-review manuscripts.

We appreciate the time and effort the Reviewer took in reviewing our manuscript.

We are submitting the revised manuscript (NCOMMS-25-60793A), entitled "Misregulation of T-box transcription factors drives BNP–NPR1 signalling underlying chronic itch" upon Reviewers' thoughtful suggestions. We have revised the manuscript based upon the comments. According to the Reviewer's suggestions, we performed additional experiments and revised the manuscript. Detailed point-to-point responses are described below. With these revisions (highlighted in yellow in the marked copy of the revision), we hope this manuscript is now acceptable for Nature Communications.

REVIEWER COMMENTS

Reviewer #1 (Remarks to the Author):

While the authors have made significant progress in addressing the previous concerns, several critical points remain. First, the Npr1 expression profile in the sensory ganglia has not been sufficiently characterized. It is essential to clarify whether the reported expression and functional data were derived from naïve (normal) mice or atopic dermatitis models as well as the data is not convincingly demonstrated the expression of Npr1 in the sensory neurons. Additionally, to strengthen the mechanistic link, the authors should include expression data for Nppb and Npr1 in ear skin within Figure 5. Finally, regarding Figure 3C, the inclusion of siRNA-mediated knockdown as a control is necessary to validate the specificity of the various cytokine releases.

Response: We greatly appreciate the reviewer's comments and valuable suggestions to further improve our manuscript.

We would like to clarify that all mouse expression and functional data in relation to the Npr1 expression profile in the sensory ganglia presented in this study, including all RNAscope experiments, were derived exclusively from the MC903-induced atopic dermatitis model, and not from naïve (untreated) mice. This is already specified in the manuscript (**P31, L875–882**), where the experiments are described.

With respect to NPR1 expression in sensory neurons, our RNAscope data demonstrate that Npr1 transcripts are present within the trigeminal ganglia from MC903-treated mice, with Npr1 transcripts are present in $Nppb^{+}$ neurons significantly reduced signal in $TBX20^{fl/fl}$;Avil-Cre mice (**Fig. 9c**), supporting neuronal expression and TBX20-dependent regulation. In addition, we provide RNAscope data from normal human DRG sections, which also show NPR1 expression in sensory neurons.

To avoid any misunderstanding, we reiterate that all mouse RNAscope experiments were performed under MC903-induced atopic dermatitis conditions, and no naïve mouse data were generated in this study.

Regarding baseline expression in naïve mice, previous studies have reported Npr1/Npr1-related signaling components in DRG neurons (e.g., PMID: 25420068; PMID: 26635247). Our study does not aim to re-establish baseline expression, but rather to investigate the regulation of this pathway in the context of atopic dermatitis.

For example, Usoskin et al. performed single-cell RNA-seq of naïve mouse DRG neurons and detected Npr1 expression in a small subset of sensory neurons, including

NP, NF, and PEP subtypes (Usoskin D. et al., Nat Neurosci 2015, PMID: 25420068). Based on their dataset, our UMAP plot of Npr1 expression across the single cells is shown below. Npr1 expression is restricted to a small subset of neurons, with modest enrichment in specific pruriceptor populations and minimal expression in other sensory neuron types.

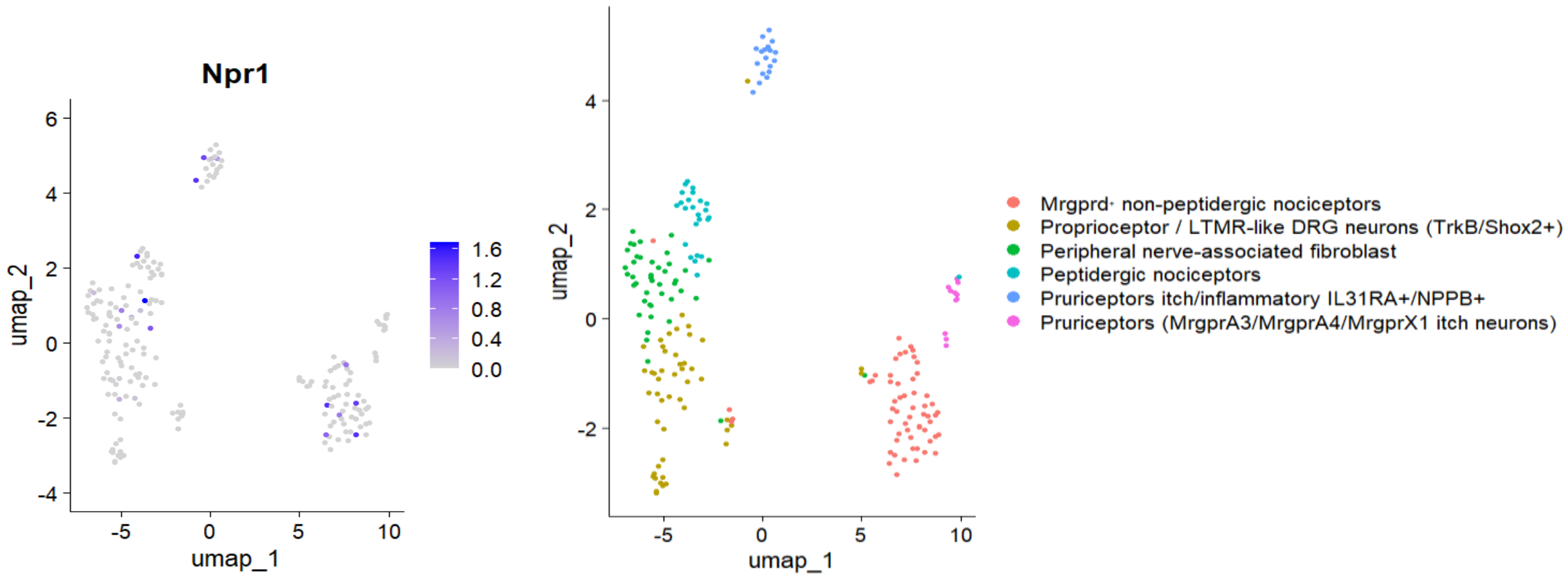

Similarly, Li et al. also reported Npr1 expression in a subset of DRG neurons using high-coverage single-cell RNA-seq (Cell Res. 2016. PMID: 26635247). These datasets consistently indicate that Npr1 expression in naïve DRG neurons. Based on their dataset, out UMAP plot of Npr1 expression across the single cells is shown below.

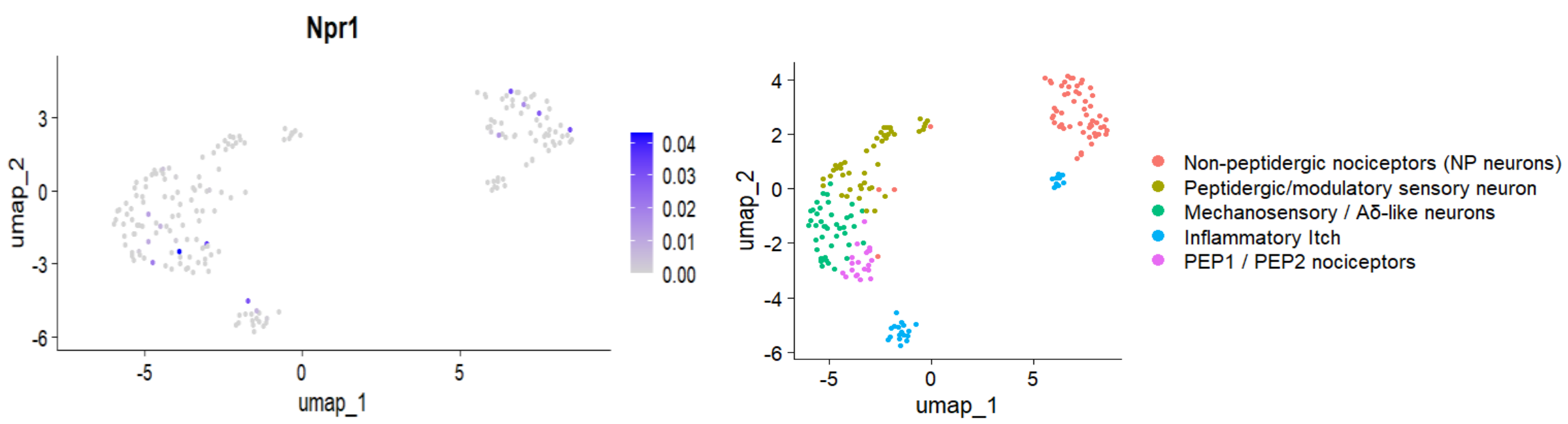

Our findings in **Fig. 9c** in MC903 model extended previous baseline expression reports by demonstrating disease-associated regulation of Npr1 signaling in sensory neurons is regulated by TBX20.

We appreciate the reviewer's insightful thoughts regarding further examining the expression of Nppb and Npr1 in ear skin in **Fig. 5**. We previously showed that transcription of both Nppb and Npr1 in the left ear skin from MC903 ear model was reduced by Lcn-2 knockout (**Fig. 5c**). In the current revision, we further investigated whether Lcn-2 deletion affects DNFB-induced Nppb and Npr1 expression compared with WT mice. Using RT-qPCR, we found that Nppb transcription in the left ear in DNFB model was significantly reduced, while Npr1 showed a decreasing trend. This new results have been added to the new **Fig. 5g**.

The text has been updated as follows, and are highlighted in yellow:

(**P28L761-762**) "Similarly, Lcn-2 knockout significantly suppressed DNFB-induced itch compared with WT mice, without affecting scratching in vehicle-treated controls (**Fig. 5e**). Baseline scratching monitored one hour prior to DNFB application remained minimal (**Fig. 5f**). *Nppb* transcription in the left ear was significantly reduced, whereas *Npr1* exhibited a downward trend (**Fig. 5g**)."

We thank the reviewer for the valuable recommendation regarding the suggestion to include siRNA-mediated knockdown as a control in **Fig. 3c** to validate the specificity of the cytokine releases. Due to the low transfection efficiency of siRNA in primary cells, we initially attempted lentiviral shRNA-mediated knockdown TBX20 in primary murine keratinocytes. However, we were unable to achieve efficient knockdown in our primary murine keratinocytes isolated from mice.

To address the reviewer's concern, we then performed lentiviral shRNA–mediated knockdown of TBX20 in cultured normal human epidermal keratinocytes (NHEK; purchased from Lonza), confirmed the knockdown by western blotting. Following stimulation with BNP (1 μM) for 24 hours, cytokine release in the NHEK supernatant was assessed and compared with NHEK infected with non-targeting control lentiviral particles. TBX20 knockdown resulted in a significant reduction in BNP-induced release of multiple cytokines. Although TBX20 knockdown could not be achieved in primary murine keratinocytes, the NHEK data nonetheless provide additional support for a role of TBX20 in BNP-induced cytokine signalling.

These new results have been added to new **Supplementary Fig. 4.** The related text has been updated as follows and are highlighted in yellow:

(**P25L673–680)** "To verify that TBX20 mediates BNP-induced cytokine release from human keratinocytes, NHEK cells were cultured and infected with lentiviral TBX20 shRNA or non-targeted control viral particles prior to analysis of BNP-induced cytokines in the culture supernatant using Proteome Profiler Human XL Cytokine Array kit. Although the detection profiles differed from those observed in mouse samples, TBX20 knockdown, verified by immunoblot (**Supplementary Fig. 4a**), reduced multiple cytokines, IL-19, IL-4, IL-24, IL-3 and IL-5 (**Supplementary Fig. 4b**), which are known to contribute to TH2-driven immune activation and keratinocyte-mediated cutaneous inflammation."

We deeply appreciate the time and effort the Reviewer took in reviewing our manuscript.

Reviewer #2 (Remarks to the Author):

The authors have conducted comprehensive revision of the previous version and adequately addressed all the questions and comments made in the 1st round of review. The current version is well-organized, presented in a clear and accessible style for readers.

Response: We deeply appreciate the time and effort the Reviewer took in reviewing our manuscript.

Reviewer #3 (Remarks to the Author):

Reviewers have nicely addressed my previous concerns

Response: We deeply appreciate the time and effort the Reviewer took in reviewing our manuscript.

Reviewer #4 (Remarks to the Author):

I co-reviewed this manuscript with one of the reviewers who provided the listed reports. This is part of the Nature Communications initiative to facilitate training in peer review and to provide appropriate recognition for Early Career Researchers who co-review manuscripts.

Response: We deeply appreciate the time and effort the Reviewer took in reviewing our manuscript.
