# Supplementary material for: Misregulation of T-box transcription factors drives BNP–NPR1 signalling underlying chronic itch
Source: Nat Commun. 2026 Jul 17;17:8754. doi: 10.1038/s41467-026-75726-x (PMC13493844; doi:10.1038/s41467-026-75726-x)
Supplement: Supplementary file 3 [file 41467_2026_75726_MOESM3_ESM.pdf]

nature portfolio

Corresponding author(s): Jianghui Meng

Last updated by author(s): Jun 16, 2026

Reporting Summary

Nature Portfolio wishes to improve the reproducibility of the work that we publish. This form provides structure for consistency and transparency in reporting. For further information on Nature Portfolio policies, see our Editorial Policies and the Editorial Policy Checklist.

Statistics

For all statistical analyses, confirm that the following items are present in the figure legend, table legend, main text, or Methods section.

n/a	Confirmed	
☐	☒	The exact sample size (*n*) for each experimental group/condition, given as a discrete number and unit of measurement
☐	☒	A statement on whether measurements were taken from distinct samples or whether the same sample was measured repeatedly
☐	☒	The statistical test(s) used AND whether they are one- or two-sided<br>*Only common tests should be described solely by name; describe more complex techniques in the Methods section.*
☒	☐	A description of all covariates tested
☐	☒	A description of any assumptions or corrections, such as tests of normality and adjustment for multiple comparisons
☐	☒	A full description of the statistical parameters including central tendency (e.g. means) or other basic estimates (e.g. regression coefficient) AND variation (e.g. standard deviation) or associated estimates of uncertainty (e.g. confidence intervals)
☐	☒	For null hypothesis testing, the test statistic (e.g. F, t, r) with confidence intervals, effect sizes, degrees of freedom and P value noted<br>*Give P values as exact values whenever suitable.*
☒	☐	For Bayesian analysis, information on the choice of priors and Markov chain Monte Carlo settings
☒	☐	For hierarchical and complex designs, identification of the appropriate level for tests and full reporting of outcomes
☒	☐	Estimates of effect sizes (e.g. Cohen's *d*, Pearson's *r*), indicating how they were calculated

Our web collection on statistics for biologists contains articles on many of the points above.

Software and code

Policy information about availability of computer code

Data collection	The Real-Time PCR Detection System (ABI, Thermo Fisher Scientific, USA) was used for qRT-PCR; the G-Box Syngene system (UK) was used for western blotting and cytokine array; Olympus cellsens standard 2.0 IX73 (Japan) was used for in situ tissue and cell imaging; animal behaviours are recorded by Noldus Ethovision XT 14 (Netherlands); Illumina pipeline for the HiSeq 2500 was used for RNA-sequencing; the Infinite M200 Fluorescence Microplate Reader (Tecan, Männendorf, Switzerland) was used for transient dual-luciferase reporter assay.
Data analysis	Dr.Tom&GENEAN were used for RNA-seq gene expression abundance analysis and Heatmap analysis (BGI, CHINA); All data analysis was performed in R-studio (v 4.4.1). The immunofluorescence data and cytokine array were analyzed and compared using ImageJ software. The number of immune cells and the thickness of the tissue epidermal layer were measured using CellSens Dimension software. Prism v.8.0 (GraphPad) software was used in the two-sided paired Student's t-test and ANOVA test; GraphPad Prism 8.0 were used for the graphical drawing.

For manuscripts utilizing custom algorithms or software that are central to the research but not yet described in published literature, software must be made available to editors and reviewers. We strongly encourage code deposition in a community repository (e.g. GitHub). See the Nature Portfolio guidelines for submitting code & software for further information.

Data

Policy information about availability of data

All manuscripts must include a data availability statement. This statement should provide the following information, where applicable:

- Accession codes, unique identifiers, or web links for publicly available datasets
- A description of any restrictions on data availability
- For clinical datasets or third party data, please ensure that the statement adheres to our policy

Anonymized dataset used for this study is made available publicly through a data repository. All data decribed in this study are included in the manuscript and the accompanying Supporting Information. All RNA-seq datasets associated with this article have been deposited in the Gene Expression Omnibus (GEO) under accession code GSE281758 [https://www.ncbi.nlm.nih.gov/gds/]. All relevant raw data from figures generated in this study have been provided in the Source Data file. Source Data are provided with this paper.
This study did not involve the development of custom code. Accordingly, no custom code was generated or deposited in a public repository. Code used for the identification of TBX20 consensus sequences in ChIP peak calling related to Fig. 3a is provided in the Source Data file. These analyses were performed using established, publicly available software and packages.

Research involving human participants, their data, or biological material

Policy information about studies with human participants or human data. See also policy information about sex, gender (identity/presentation), and sexual orientation and race, ethnicity and racism.

Reporting on sex and gender	No reporting on sex and gender was referenced in this manuscript.
Reporting on race, ethnicity, or other socially relevant groupings	No Reporting on race, ethnicity, or other socially relevant groupings.
Population characteristics	No Reporting on population characteristics..
Recruitment	No human participant recruitment or compensation was performed by the authors.
Ethics oversight	The human skin tissues were purchased from Tissue Solutions (Glasgow, Scotland); thus, obtaining additional Institutional Review Board approval and written informed consent was not necessary at as ethical approval and written informed consent was obtained from Tissue Solutions for the sample procurement.

Note that full information on the approval of the study protocol must also be provided in the manuscript.

Field-specific reporting

Please select the one below that is the best fit for your research. If you are not sure, read the appropriate sections before making your selection.

☒ Life sciences ☐ Behavioural & social sciences ☐ Ecological, evolutionary & environmental sciences

For a reference copy of the document with all sections, see nature.com/documents/nr-reporting-summary-flat.pdf

Life sciences study design

All studies must disclose on these points even when the disclosure is negative.

Sample size	Biological replicates were used for gene expression analyses and animal behaviour assays. Phenotypic analyses and physiological measurements were performed using three or more independent biological replicates. Sample sizes were selected based on established practice in the field and/or previous studies.<br>Cytokine profiling was performed in a single experiment without independent biological replicates; therefore, the results are presented as descriptive, exploratory data only, and no statistical analyses were performed.
Data exclusions	No data were excluded.
Replication	All samples were randomly allocated into experimental groups and there is no intentional choice.
Randomization	All samples were randomly allocated into experimental groups and there is no intentional choice.
Blinding	Blinding was used for behavior study.

Reporting for specific materials, systems and methods

We require information from authors about some types of materials, experimental systems and methods used in many studies. Here, indicate whether each material, system or method listed is relevant to your study. If you are not sure if a list item applies to your research, read the appropriate section before selecting a response.

Materials & experimental systems

n/a	Involved in the study
☐	☒ Antibodies
☐	☒ Eukaryotic cell lines
☒	☐ Palaeontology and archaeology
☐	☒ Animals and other organisms
☒	☐ Clinical data
☒	☐ Dual use research of concern
☒	☐ Plants

Methods

n/a	Involved in the study
☐	☒ ChIP-seq
☒	☐ Flow cytometry
☒	☐ MRI-based neuroimaging

Antibodies

Antibodies used

Anti-FLG2 (NBP1-91901 (1:500), Novus);
Anti- LOR(905103 (1:500), Biolegend);
Anti- PGP9.5(AB108186 (1:500), Abcam);
Anti- CD11b(AB13357 (1:500), Abcam);
Anti- MCPT8(647401 (1:500), Biolegend);
Anti- CD68(AB283654 (1:100). Abcam)
Anti-PGP9.5(AB8189 (1:500), Abcam)
Anti- IL-31RA(AF2107 (1:200), R&D)
Anti- TBX20(AV33177 (1:100), Sigma-Aldrich)
Anti- CD4 (AB183685 (1:200), Abcam)
Anti- K14(NBP2-34355(1:500), Novus)
Anti- LCN-2(PA5-116987 (1:250), Thermo Fisher)
Goat Anti-Rat 488(AB150165 (1:500), Abcam)
Goat Anti-Rabbit 488(AB150077 (1:500), Abcam)
Goat Anti-Rat 594(AB150160 (1:500), Abcam)
Goat Anti-Mouse 594(AB150111 (1:500), Abcam)
Goat Anti-Mouse 488(AB150133 (1:500), Abcam)

Validation

Anti-FLG2 (NBP1-91901 (1:500), Novus) validation could be found in the website: https://www.novusbio.com/products/filaggrin-family-member-2-antibody_nbp1-91901?srsltid=AfmBOooaa3F58quqekip8OZeSl1qCGEJibFRrUYlxXRY34aO8khqrluN;
Anti- LOR(905103 (1:500), Biolegend) validation could be found in the website: https://www.biolegend.com/nl-nl/products/purified-anti-loricrin-antibody-13325
Anti- PGP9.5(AB108186 (1:500), Abcam) validation could be found in the website: https://www.abcam.com/en-us/products/primary-antibodies/pgp95-antibody-epr4118-neuronal-marker-ab108986?srsltid=AfmBOopHqthAsq8iB9ZN4ZqOpEqxGHqkvsTNKmBZoDJ7Sm7Uz7CWUfLg
Anti- CD11b(AB133357 (1:500), Abcam) validation could be found in the website: https://www.abcam.com/en-us/products/primary-antibodies/cd11b-antibody-epr1344-ab133357?srsltid=AfmBOopkA6djys3MDMw6q62HOaV3o7gFfhFbt3ytAaK7KMXxPOSmAqx_
Anti- MCPT8(647401 (1:500), Biolegend) validation could be found in the website: https://www.biolegend.com/de-de/products/purified-anti-mmcp-8-antibody-6266
Anti- CD68(AB283654 (1:100). Abcam) validation could be found in the website: https://www.abcam.com/en-us/products/primary-antibodies/cd68-antibody-epr23917-164-ab283654
Anti-PGP9.5(AB8189 (1:500), Abcam) validation could be found in the website: https://www.abcam.com/en-us/products/primary-antibodies/pgp95-antibody-13c4-i3c4-neuronal-marker-ab8189
Anti- IL-31RA(AF2107 (1:200), R&D) validation could be found in the website: https://www.rndsystems.com/products/mouse-il-31ra-antibody_af2107
Anti- TBX20(AV33177 (1:100), Sigma-Aldrich) validation could be found in the website: https://www.sigmaaldrich.com/IE/en/product/sigma/av33177?srsltid=AfmBOooYDLEX8hbYZo7dz6HRjvYsNZ9zyaWg0O1ZS5JCsffCZx5_jwZS
Anti- K14(NBP2-34355(1:500), Novus) validation could be found in the website: https://www.novusbio.com/products/cytokeratin-14-antibody-spm263_nbp2-34355
Anti- CD4 (AB183685 (1:200), Abcam) validation could be found in the website: https://www.abcam.com/en-us/products/primary-antibodies/cd4-antibody-epr19514-ab183685
Anti- LCN-2(PA5-116987 (1:250), Thermo Fisher) validation could be found in the website: https://www.thermofisher.com/antibody/product/NGAL-Antibody-Polyclonal/PA5-116987
Goat Anti-Rat 488(AB150165 (1:500), Abcam) validation could be found in the website: https://www.abcam.com/en-us/products/secondary-antibodies/goat-rat-igg-h-l-alexa-fluor-488-preadsorbed-ab150165?srsltid=AfmBOorWLBbjlbn6fNSGQROOu--gEL_gW7YVqJ6-XOOK57deNrh6LXMR
Goat Anti-Rabbit 488(AB150077 (1:500), Abcam) validation could be found in the website: https://www.abcam.com/en-us/products/secondary-antibodies/goat-rabbit-igg-h-l-alexa-fluor-488-ab150077
Goat Anti-Rat 594(AB150160 (1:500), Abcam) validation could be found in the website: https://www.abcam.com/en-us/products/secondary-antibodies/goat-rat-igg-h-l-alexa-fluor-594-ab150160
Goat Anti-Mouse 594(AB150116 (1:500), Abcam) validation could be found in the website: https://www.abcam.com/en-us/search?productSorting=relevance&resourceSorting=relevance&keywords=goat+anti-mouse+594&productcode=ab150116&view=quickview
Goat Anti-Mouse 488(AB150133 (1:500), Abcam) validation could be found in the website: https://www.abcam.com/en-us/products/

secondary-antibodies/donkey-goat-igg-h-l-alexa-fluor-488-preadsorbed-ab150133

Eukaryotic cell lines

Policy information about cell lines and Sex and Gender in Research

Cell line source(s)	NHEK cells were obtained from Lonza Inc.<br>HaCaT cells were obtained from iCell Bioscience Inc.<br>HEK293 cells were from iCell Bioscience Inc.
Authentication	HaCaT and HEK293 cell lines were authenticated by STR profiling prior to use. Primary NHEKs were obtained from Lonza and used according to the manufacturer's specifications.
Mycoplasma contamination	The cells were tested negatively for mycoplasma contamination
Commonly misidentified lines (See ICLAC register)	No commonly misidentified lines were used in this study.

Animals and other research organisms

Policy information about studies involving animals; ARRIVE guidelines recommended for reporting animal research, and Sex and Gender in Research

Laboratory animals	6-8 weeks old mice (BW: 16-18g) were used for behaviour-scoring purposes; C57BL/6 (Mus musculus) breeder mice were obtained from SPF (Beijing) Biotechnology Co. Ltd. which we bred in-house. Cell-specific TBX20 knockout mice (cKO) we crossed K14-Cre transgenic mice (genetic background C57BL/6N, Cyagen) or Avil-Cre-transgenic mice (Shanghai Model Organisms Center, Inc) with "floxed" TBX20 (TBX20fl/fl) mice (genetic background C57BL/6J). Progeny were genotyped by PCR as described. cKO TBX20fl/fl;K14-Cre and TBX20fl/fl;Avil-Cre were specifically deficient in keratinocytes and sensory neurons respectively, and only female mice were tested.<br>Whole body LCN-2 knockout mouse strain C57BL/6JCya-Lcn2em1/Cya (S-KO-02865) was purchased from Cyagen (genetic background C57BL/6J). Only male LCN-2 knockout transgenic mice and WT mice were used.
Wild animals	The study did not involve wild animals.
Reporting on sex	The study was conducted with female TBX20 transgenic mice and male LCN-2 transgenic mice.
Field-collected samples	The study did not involve field-collected samples.
Ethics oversight	All animal procedures were performed in accordance with the Guidelines for Care and Use of Laboratory Animals of Henan University and approved by the Animal Ethics Committee of Henan University.

Note that full information on the approval of the study protocol must also be provided in the manuscript.

Plants

Seed stocks	n/a
Novel plant genotypes	n/a
Authentication	n/a

ChIP-seq

Data deposition

☒ Confirm that both raw and final processed data have been deposited in a public database such as GEO.

☐ Confirm that you have deposited or provided access to graph files (e.g. BED files) for the called peaks.

Data access links<br>*May remain private before publication.*	This study did not involve the development of custom code. Accordingly, no custom code was generated or deposited in a public repository. Code used to identify TBX20 consensus sequences in ChIP-seq peak analyses related to Fig. 3a is included in the Source Data file and is reproduced below. These analyses were performed using established, publicly available software and packages.

ENCODE Data
H3K4me 1 foreskin keratinocyte (Homo sapiens) Accession #: ENCSR780KNM H3K4me2 foreskin keratinocyte (Homo sapiens) Accession #: ENCSR926BCX H3K4me3   keratinocyte male from donor(s) Accession #: ENCSR113UDS H3K27ac female keratinocyte (Homo sapiens) Accession #: ENCSR000ALK

Reference Genome Used: Ensembl Hg19 region 153,621,113 to 153,666,468

Files in database submission

The authors did not generate raw data for ChIP-seq.

Genome browser session
(e.g. UCSC)

Previously published datasets were used for ChIP-seq anslysis. These are from the following references:
References
1) Adam Siepel, Gill Berejano, Jakob S. Pedersen, Angie S. Hinrichs, Minmei Hou, Kate Rosenbloom, Hiram Clawson, John Spieth, LaDeana W. Hillier, Stephen Richards, George M. Weinstock, Richard K. Wilson, Richard A. Gibbs, W. James Kent, Webb Miller, David Haussler (2005). "Evolutionarily conserved elements in vertebrate, insect, worm, and yeast genomes." _Genome Research_, *15*, 1034-1050. doi:10.1101/gr.3715005

2) Katherine S. Pollard, Melissa J. Hubisz, Kate R. Rosenbloom, Adam Siepel (2010). "Detection of nonneutral substitution rates on mammalian phylogenies." _Genome Research_, *20*, 110-121. doi:10.1101/gr.097857.109

Methodology

Replicates	N/A
Sequencing depth	N/A
Antibodies	N/A
Peak calling parameters	N/A
Data quality	N/A
Software	N/A
